# Tripartite ER-mitochondria-lipid droplets contact sites control adipocyte metabolic flexibility

**DOI:** 10.1038/s44318-026-00876-z

**Published:** 2026-07-22

**Authors:** Marie Palard, Gilliane Chadeuf, Maxime Carpentier, Clotilde C N Renaud, Samuel Frey, Mikael Croyal, Rola Shaaban, Samy Hadjadj, Chloe Cloteau, Naima El Khallouki, Perrine Bomme, Soazig Le Lay, Manuel Majrouh, Perrine Paul-Gilloteaux, Alexandre Humbert, Christine Durand, Nicolas Bertocchini, Laurence Dubreil, Yoann Combot, Antria Antreou, Cedric Le May, Simon Ducheix, Bertrand Cariou, Francesca Giordano, Jennifer Rieusset, Abdou Rachid Thiam, Xavier Prieur

**Affiliations:** 1 Université de Nantes, CNRS, INSERM, l’institut du thorax, 44000 Nantes, France; 2 Laboratoire de Physique de l’École normale supérieure, ENS, Université PSL, CNRS, Sorbonne Université, Université Paris Cité, F-75005 Paris, France; 3 Nantes Université, CNRS, CHU Nantes, INSERM, l’institut du thorax, F-44000 Nantes, France; 4 Nantes Université, CHU Nantes, INSERM, CNRS, SFR Santé, Inserm UMS 016, Nantes, France; 5 Université Paris-Saclay, CEA, CNRS, Institute for Integrative Biology of the Cell (I2BC), 91198 Gif-sur-Yvette, France; 6 Inserm U1280, Gif-sur-Yvette, cedex 91198 France; 7 Institut Pasteur, Université Paris Cité, Ultrastructural Bioimaging Unit, 75015 Paris, France; 8 Univ Angers, SFR ICAT, 49000 Angers, France; 9 Nantes Université, CHU Nantes, CNRS, Inserm, BioCore, US16, SFR Bonamy, Nantes, France; 10 Laboratoire CarMeN, UMR INSERM U1060/INRA U1397, Université Claude Bernard Lyon1, Pierre-Bénite, France; 11 Oniris, INRAE, PAnTher, Nantes, 44300 France

**Keywords:** Membranes & Trafficking, Metabolism, Organelles

## Abstract

Adipocyte dysfunction is a major driver of obesity-associated cardiometabolic disease, underscoring the need to understand how lipid storage and mobilization are regulated and disrupted. The ER-anchored protein Seipin governs lipid droplet (LD) biogenesis and ER-LD and ER–mitochondria (MAM) contacts, and its loss impairs calcium transfer and causes lipodystrophy. Here, we investigated whether Seipin coordinates MAM and ER-LD remodeling during adipocyte lipid handling. In subcutaneous adipose tissue from inducible Seipin-knockout mice, electron microscopy and proximity ligation assays revealed that feeding reduces MAMs while increasing ER-LD and mitochondria-LD contacts, a remodeling abolished by Seipin deficiency. Lipid loading elevated tripartite MAM-LD contacts in controls but not knockouts. Fluorescence recovery after photobleaching showed that impaired triglyceride transfer to LDs in Seipin-deficient cells was rescued by the MAM-LD-stabilizing peptide ‘Linker-ER-Mi’, in a calcium-dependent manner. During adipogenesis and lipid loading, MAM-LD contacts increased, whereas MAM-cytosolic mitochondria contacts declined; however, obesity blunted this remodeling. Furthermore, disrupting membrane contact sites impaired lipid flux, lipolysis, and insulin signaling. Taken together, these findings identify MAM-LD as regulators of adipocyte metabolic flexibility.

## Synopsis

Adipocytes switch rapidly between lipid storage and lipid mobilization, yet how membrane contact sites adapt to support such metabolic transitions remains poorly understood. This study shows that dynamic remodelling of tripartite ER–mitochondria–lipid droplet contact sites is required for efficient lipid remobilisation.

- Lipid loading remodels adipocyte membrane contact sites by increasing tripartite ER–mitochondria–lipid droplet contacts while reducing ER–mitochondria contacts distant from lipid droplets.
- Seipin deficiency abolishes this nutrient-induced remodeling and impairs triglyceride transfer from the ER to lipid droplets.
- Reinforcing ER–mitochondria contacts with a synthetic linker restores ER-to-lipid droplet triglyceride flux in Seipin-deficient adipocytes through a calcium-dependent mechanism.
- Obesity is associated with loss of nutrient-dependent membrane contact site remodeling in adipose tissue.
- Genetic disruption of membrane contact sites impairs lipid transfer to lipid droplets, lipolysis, and insulin signaling in adipocytes.

## Abstract (web summary)

Adipocytes rewire organelle contacts during metabolic transitions.

## Graphical abstract

**Figure:**
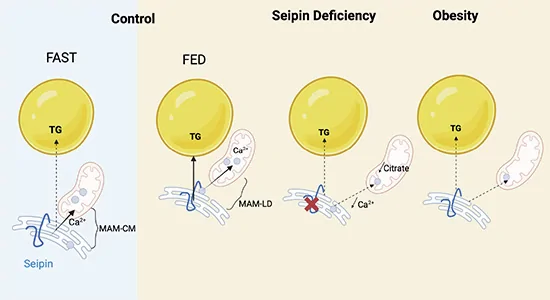

**Figure:**
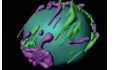

## Introduction

The increasing prevalence of obesity is driving a rise in cardiometabolic morbidities, with adipocyte dysfunction playing a central role (Virtue and Vidal-Puig, 2010). In obese individuals, adipose tissue (AT) exhibits significant impairments in both lipid storage and mobilization. Stable isotope tracer studies show that, under normal conditions, lean individuals efficiently store lipids postprandially and release them during fasting. In contrast, these processes are markedly impaired in individuals with obesity, reflecting a profound metabolic inflexibility (McQuaid et al, 2011). Similarly, the use of atmospheric ^14^C incorporation into lipids reveals decreased lipid turnover in obese subjects (Arner et al, 2011). These findings suggest that in obesity, AT enters a state of metabolic inertia, reducing its capacity to store excess lipids and promoting ectopic lipid accumulation in non-adipose tissues—thereby contributing to cardiometabolic complications (Klein et al, 2022). Furthermore, recent studies indicate that even after weight loss, AT retains features of dysfunction that predispose to weight regain and exacerbate metabolic complications (Hinte et al, 2024). This highlights the urgent need to better understand the mechanisms governing lipid storage and mobilization in adipocytes and how these processes are dysregulated in obesity.

Congenital generalized lipodystrophy (CGL) represents the most severe form of primary adipocyte dysfunction, characterized by a near-complete absence of AT and severe metabolic complications (Hussain and Garg, 2016). Biallelic mutations in the *BSCL2* gene, which encodes the endoplasmic reticulum (ER)-anchored protein Seipin, are the most common cause of CGL (Magre et al, 2001). Seipin-deficient mice display severe lipoatrophy and extreme insulin resistance (Magre and Prieur, 2022). Furthermore, inducible Seipin deficiency leads to rapid AT loss (Zhou et al, 2015). Given the severity of the phenotype associated with Seipin deficiency, we and others hypothesize that elucidating Seipin’s function could reveal novel pathways that regulate adipocyte homeostasis (Magre and Prieur, 2022). Early studies in yeast demonstrated that Seipin deficiency leads to abnormal lipid droplet (LD) morphology (Fei et al, 2008; Szymanski et al, 2007). Seipin is essential for both the initiation of LD biogenesis (Cartwright et al, 2015) and the transition to mature LDs (Wang et al, 2016). Seipin is enriched at ER–LD contact sites, and in fibroblasts derived from patients with Seipin deficiency, these contacts appear disorganized (Salo et al, 2016). In addition, we have previously shown that, in adipocytes, Seipin is enriched at ER–mitochondria contact sites (also known as mitochondria-associated membranes, or MAMs), and that Seipin loss disrupted ER-to-mitochondria calcium flux (Combot et al, 2022). Importantly, in fed conditions, Seipin is enriched in ER–LD contacts, whereas starvation promotes its relocalization to MAMs. Whether Seipin recruitment to MAMs and ER–LD contact sites is mutually exclusive remains unknown. Additionally, the function of Seipin at MAMs is not well understood, and it is unclear whether Seipin localized at MAMs contributes to its central role in regulating lipid storage in adipocytes.

In the past decade, membrane contact sites (MCS) have emerged as central regulators of cellular metabolic flexibility, i.e., the capacity to adapt fuel utilization to nutritional and hormonal states (Goodpaster and Sparks, 2017). Among these, MAMs serve as key hubs for lipid and calcium exchange. By integrating nutrient and hormonal cues, MAMs modulate both insulin sensitivity and metabolic flexibility (Rieusset, 2018; Theurey and Rieusset, 2017). In the liver, disruption of MAMs promotes insulin resistance and metabolic dysfunction such as MASLD, whereas reinforcement of these contacts protects high-fat diet (HFD)-fed mice from metabolic complications (Beaulant et al, 2022). ER–LD contact sites are also critical for lipid storage, and their perturbation has been linked to various metabolic diseases (Herker et al, 2021). Indeed, LDs originate from the ER (Chen and Goodman, 2017) and remains physically contiguous with it to exchange proteins and lipids and can also establish non-contiguous contacts with the ER throughout their lifetime. More recently, mitochondria–LD (Mi–LD) contact sites have emerged as essential for lipid homeostasis in both anabolic and catabolic processes. In brown AT, two distinct mitochondrial subpopulations have been described: cytosolic mitochondria (CM) and peri-droplet mitochondria (PDM) (Benador et al, 2018). In the liver, PDM are thought to promote lipogenesis, while CM primarily support catabolic processes (Najt et al, 2023). This subcellular heterogeneity also complexifies our understanding of MAMs: MAMs involving CM (MAM-CM) would be the “classical” MAMs, known to support catabolic processes during fasting (Theurey and Rieusset, 2017), while those involving PDM (referred to as MAM–LDs) are enriched in lipid synthesis enzymes (Najt et al, 2023). To date, very few reports have focused on MAM-LDs, and to our knowledge, none have examined these in adipocytes.

In this study, we seek to verify the hypothesis that alterations in organelle contact sites could contribute to adipocyte dysfunction and thus represent a new molecular mechanism underlying cardiometabolic complications associated with obesity and lipodystrophies. We firstly characterized the MCS in Seipin-deficient adipocytes, and we assessed their functional involvement in lipid handling. Then, we characterize adipocyte’s MCS in the most common form of adipocyte dysfunction by analysing the AT from obese mice. Finally, in vitro, we used several genetic interventions to specifically assess the consequences of MCS disturbances on adipocyte homeostasis.

## Results

### Seipin deficiency alters MAM-LD contacts and abolishes the nutrient-driven organelle remodeling in vivo in mice

**Figure EV1:**
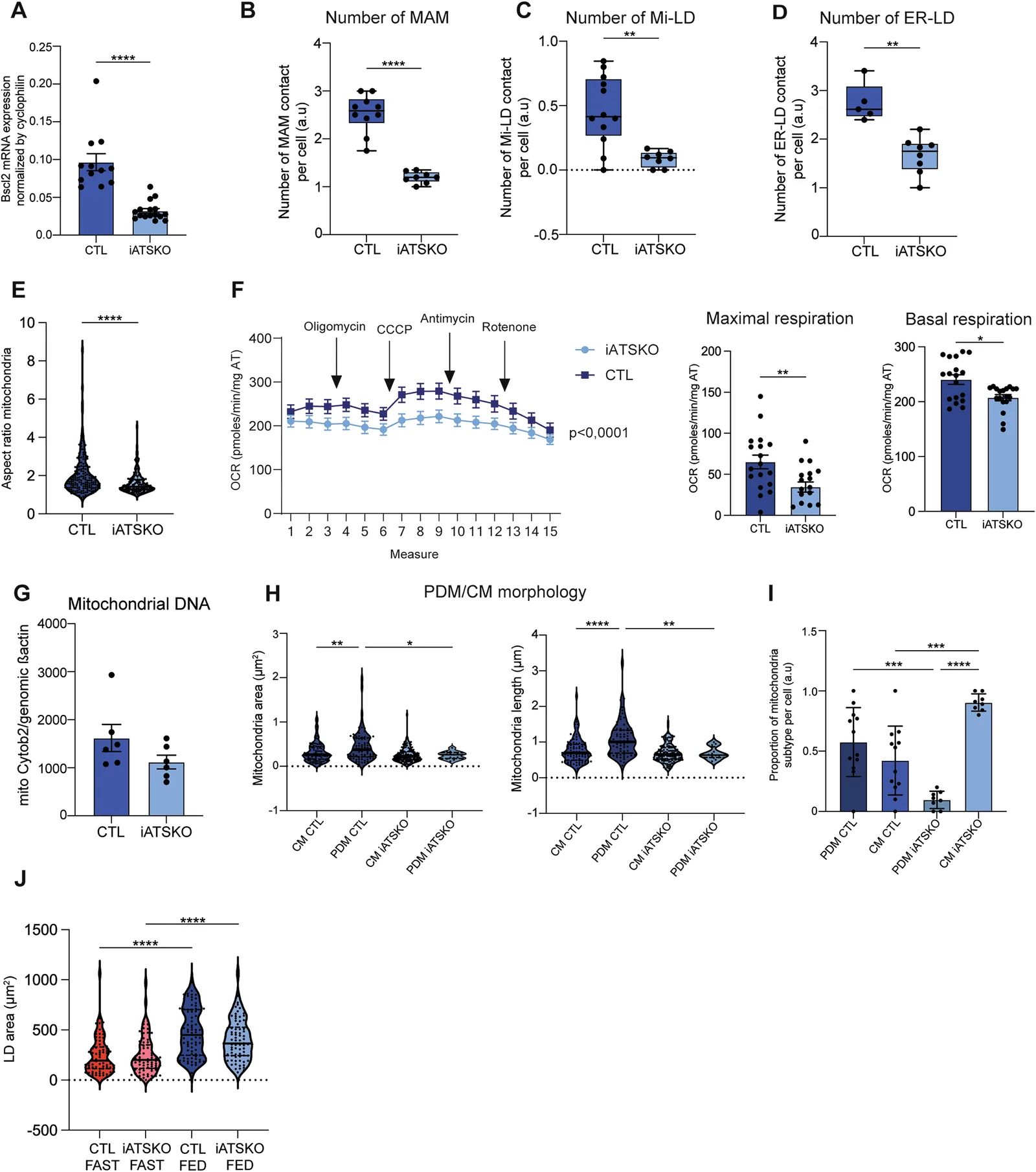
Seipin deficiency alters adipose tissue organelle organization and mitochondrial function. (**A**) *Bscl2* mRNA expression was quantified by RT-qPCR in inguinal adipose tissue from *Bscl2*^*lox/lox*^ (Control) and *Bscl2*^*lox/lox*^*ERT2-Adipoq-Cre* (iATSKO) mice collected in the fed state, 14 days after tamoxifen injection (100 mg/kg). (**B**–**D**) TEM analyses were performed to quantify the number of MAMs (**B**), Mi–LD contacts (**C**), and ER–LD contacts (**D**) per cell. (**E**) Mitochondrial aspect ratio was quantified using Fiji. (**F**) Seahorse analysis was performed on 2 mg explants of subcutaneous adipose tissue. (**G**) Mitochondrial DNA content was quantified by measuring mitochondrial cytochrome *b* DNA normalized to genomic β-actin DNA. (**H**,** I**) Mitochondrial area and length were quantified according to mitochondrial subtype, together with their relative distribution (**I**). (**J**) Lipid droplet surface area was quantified using PLIN1 fluorescence signal. Data information: (**A**) Bars represent mean ± SEM; *n* > 12 mice per genotype. (**B**–**D**) Box plots show the median (center line), the 25th and 75th percentiles (box), the minimum and maximum values (whiskers), and individual data points; measurements were obtained from two mice per genotype, with five cells analyzed per mouse. (**F**) Seahorse analysis was performed using three biopsies per mouse; *n* = 6 mice per genotype. (**G**) *n* = 6 mice per genotype. (**H**) Violin plots show the distribution of quantified mitochondria, with each dot representing one individual mitochondrion; median indicated by the central line; measurements were obtained from two mice per genotype, with five cells analyzed per mouse. (**I**) Bars: mean ± SD, with each dot representing a cell. Measurements were obtained from two mice per genotype, with five cells analysed per mouse. *p* < 0.05, *p* < 0.001, *p* < 0.0001, One-way ANOVA test. (**J**) Violin plots show the distribution of lipid droplet surface area, with each dot representing one lipid droplet; median indicated by the central line; measurements were obtained from three mice per genotype, with 25 cells analyzed per mouse. Statistical analysis: Mann–Whitney test (**A**–**H**); one-way ANOVA (**I**, **J**). Exact *P* values from two-tailed Mann–Whitney tests: *P* = 0.0027 (**C**) and *P* = 0.0016 (**D**). Exact adjusted *P* values from Tukey’s multiple comparisons test: contact area, PDM CTL vs. PDM iATSKO, *P* = 0.0168; contact length, PDM CTL vs. PDM iATSKO, *P* = 0.0020 (**H**). All other significant comparisons, *P* < 0.001. *p < 0.05, **p < 0.01, ***p < 0.001, ****p < 0.0001.

We previously demonstrated that Seipin localizes at the MAM (Combot et al, 2022; Guyard et al, 2022), prompting us to investigate here whether Seipin deficiency affects MAMs in AT. We analyzed subcutaneous AT from inducible adipocyte-specific Seipin-knockout mice (iATSKO) 15 days after tamoxifen injection, resulting in a 70% reduction in *Bscl2* mRNA levels (Fig. EV1A). At this stage, AT displays only mild dysfunction, allowing us to capture the early mechanisms underlying the 80% adipose mass loss observed in 3-month-old iATSKO mice.

**Figure 1:**
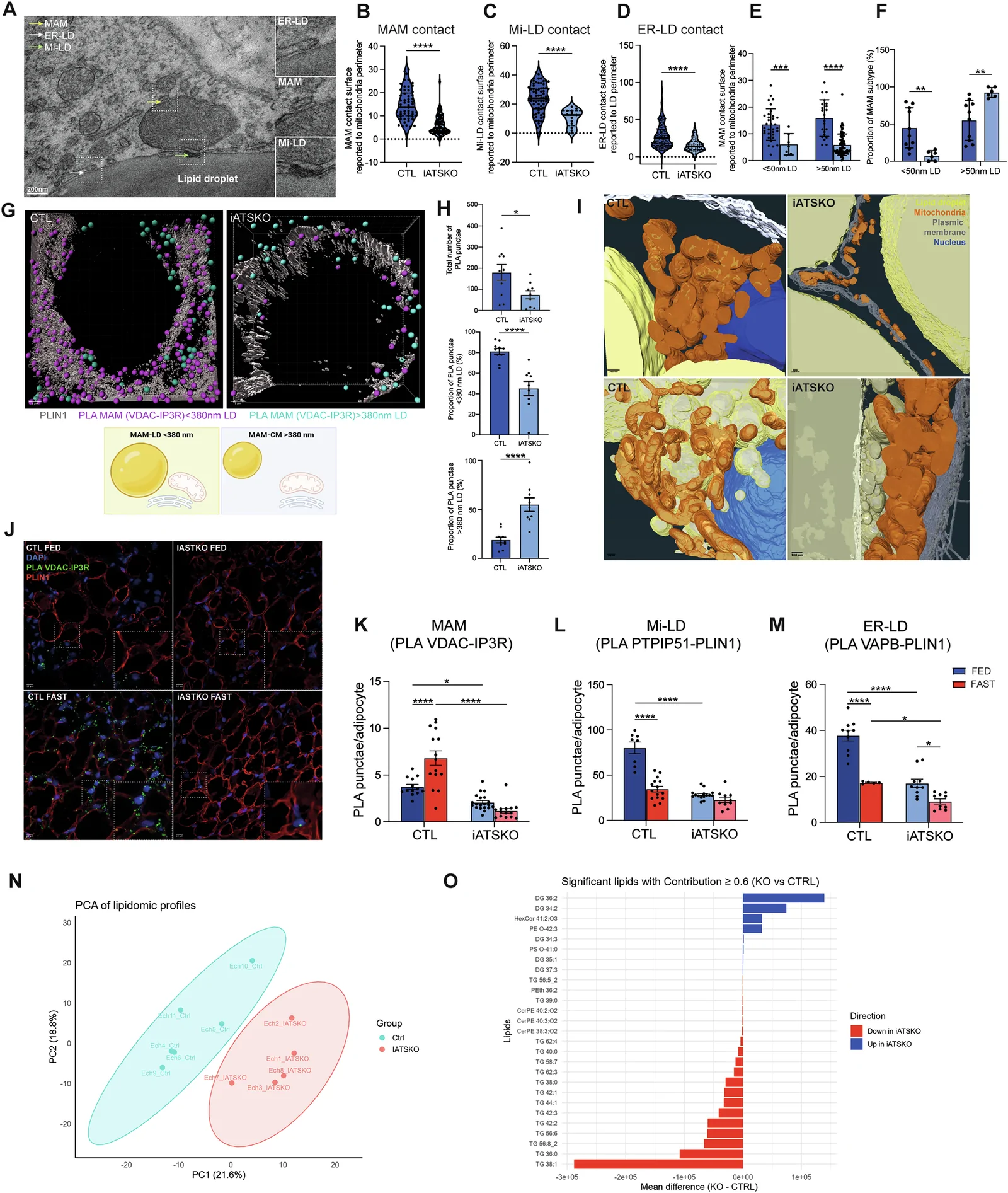
MAM–LD architecture is strongly altered in the inguinal adipose tissue of Seipin-deficient mice. (**A**–**D**) Inguinal adipose tissue was collected from *Bscl2*^*lox/lox*^ (Control) and *Bscl2*^*lox/lox*^*ERT2-Adipoq-Cre* (iATSKO) mice in the random-fed state, 14 days after tamoxifen injection (100 mg/kg). Transmission electron microscopy (TEM) images were analyzed, and the contact surface areas of mitochondria-associated membranes (MAMs), mitochondria–lipid droplet (Mi–LD), and endoplasmic reticulum–lipid droplet (ER–LD) interfaces were quantified using Fiji, as described in the Materials and Methods. (**E**–**F**) MAM subpopulations were quantified according to their proximity to lipid droplets: MAM–LD (<50 nm from LDs) and MAM–CM (>50 nm from LDs). (**G**,** H**) MAM abundance was quantified using proximity ligation assay (PLA) for IP3R and VDAC, combined with PLIN1 immunofluorescence and structured illumination microscopy (SIM). Representative images show PLIN1 (gray), MAM–LD (magenta), and MAM–CM (pink) (**G**). Total PLA signals were quantified and classified according to their distance from lipid droplets: MAM–LD (<380 nm from LDs) and MAM–CM ( > 380 nm from LDs) (**H**). (**I**) Representative focused ion beam-scanning electron microscopy (FIB-SEM) images of adipose tissue sections from Control and iATSKO mice. Organelle segmentation shows mitochondria (orange), lipid droplets (yellow), nucleus (blue), and plasma membrane (white). (**J**–**M**) Cryopreserved subcutaneous adipose tissue from Control and iATSKO mice in the fed or 18 h fasted state was analyzed by PLIN1 immunofluorescence (**J**). PLA was used to quantify MAMs (VDAC/IP3R; **K**), Mi–LD contacts (PTPIP51/PLIN1; **L**), and ER–LD contacts (VAPB/PLIN1; **M**). (**N**,** O**) Lipid droplets were isolated from Control and iATSKO mice and subjected to lipidomic analysis. Principal component analysis (PCA) is shown in (**N**), and the lipid species contributing most strongly to group separation are shown in (**O**). Data information: (**B**–**D**) Violin plots show the distribution of all quantified contacts, with each dot representing one individual contact and the median indicated by the central line. (**A**–**F**) Measurements were obtained from two mice per genotype, with five cells analyzed per mouse. (**E**, **F**) Bars represent mean ± SD. (**H**) Bars represent mean ± SEM; *n* = 5 mice per genotype. (**K**–**M**) Bars represent mean ± SEM; *n* = 5 mice per group, >100 cells per mouse. Statistical analysis: Mann–Whitney test (**B**–**D**, **H**); two-way ANOVA (**E**, **F**; **K**–**M**). For the distribution of lipid droplet diameters, the percentage of lipid droplets >50 nm was significantly different (*P* = 0.0055; two-way ANOVA, **F**). Exact adjusted *P* values for pairwise comparisons: (**H**) *P* = 0.0335; (**K**) CTL fed vs. iATSKO fed, *P* = 0.0344; (**M**) CTL fasted vs. iATSKO fasted, *P* = 0.0416; iATSKO fed vs. iATSKO fasted, *P* = 0.0121. All other significant comparisons, *P* < 0.001. *p < 0.05, **p < 0.01, ***p < 0.001, ****p < 0.0001. Source data are available online for this figure.

We used transmission electron microscopy (TEM) to quantify changes in inter-organelle contacts (Fig. 1A). Throughout this manuscript, we quantified organelle contact sites using TEM by analyzing ER–mitochondria contacts (i.e., MAMs), ER–lipid droplet (ER–LD) contacts, and mitochondria–LD (Mi–LD) contacts. Two parameters were measured: (1) the contact number, defined as the occurrence of contacts where the distance between organelles is <50 nm, and (2) contact surface, defined as the length of the contact normalized to the mitochondrial perimeter for MAMs and Mi–LD contacts, or to the LD perimeter for ER–LD contacts (as indicated in the axes and figure legends; see Appendix Fig. S1 and Methods section). Because this parameter is normalized to the mitochondrial or LD perimeter, it does not simply reflect changes in organelle size or morphology, but rather specifically reports the structural properties of the contact sites. Our analysis revealed a decrease in MAM, Mi–LD, and ER–LD contact surfaces, with Mi–LD contacts showing the most pronounced reduction (~70%) in Seipin-deficient AT compared with control (Fig. 1A–D). The number of the three contact types was similarly decreased (Fig. EV1B–D).

We then focused on mitochondrial properties and found a reduced length-to-width ratio (aspect ratio; Fig. EV1E) and impaired oxygen consumption in Seipin-deficient AT (Fig. EV1F). Of note, these changes occurred despite unchanged mitochondrial DNA content in Seipin-deficient mice (Fig. EV1G). In control mice, mitochondria engaged with LD (peri-droplet mitochondria; PDM) (Benador et al, 2018) were consistently longer and broader than cytosolic mitochondria (CM), a distinction that was lost in iATSKO adipocytes (Fig. EV1H). Additionally, the proportion of PDM and CM was roughly equal in control mice, whereas CM represented 90% of mitochondria in Seipin-deficient adipocytes (Fig. EV1I). Previous reports have revealed distinct properties of ER–mitochondria contacts involving peri-droplet mitochondria (PDMs), which are closely associated with LDs (<50 nm distance) and have been termed MAM-PDM (Najt et al, 2023) or MAM–LD (Guyard et al, 2022), compared with contacts involving cytosolic mitochondria (CM), referred to here as MAM–CM (Najt et al, 2023). Because our analytical methods do not allow us to distinguish between the ER–Mi–LD (proper MAM-PDM) and Mi–ER–LD configurations, we used the term MAM–LD, which appears more appropriate and less restrictive. Seipin deficiency reduced the surface area of both MAM–CM and MAM–LD contacts (Fig. 1E). However, whereas control mice displayed comparable proportions of MAM–CM and MAM–LD contacts, iATSKO mice exhibited a marked predominance of MAM–CM contacts (~90%), with MAM–LD contacts accounting for only ~10% of total MAMs (Fig. 1F).

To further analyze MAM-LD interactions in three dimensions, we next combined in situ proximity ligation assays (PLA) with immunofluorescence and structured illumination microscopy (SIM) to segment signals by LD proximity. As previously validated, IP3R–VDAC PLA was used to quantify MAMs (Tubbs and Rieusset, 2016). Then, following a validated protocol (Monteiro-Cardoso et al, 2023), we classified ER-Mi contacts as MAM-LD when their distance to LD was below 380 nm; otherwise, they were considered MAM-CM. This threshold was selected based on the estimated size of the PLA amplicon. According to the manufacturer, the full PLA signal complex (comprising the primary antibodies, secondary antibodies, and the amplification products generated) has an approximate diameter of 380 nm. Seipin-deficient AT showed a marked reduction in the proportion of MAM-LD, while MAM-CM percentage was increased (Fig. 1G,H). We used FIB-SEM (focused ion beam-scanning electron microscopy) to visualize the three-dimensional organization of the organelles. In samples from control mice, LD and mitochondria are closely apposed, indicating a tight spatial association. By contrast, in Seipin-deficient mice, mitochondria appear detached from LD and display a more disorganized arrangement. Due to the limited resolution, ER segmentation was not feasible in these datasets. Qualitatively, these observations support the notion that Seipin loss leads to a disruption of organelle spatial organization. (Fig. 1I; Movies EV1 and EV2).

Next, we investigated the impact of the nutritional state on contact remodeling. To this end, we used PLA to quantify contact numbers, as this approach provides a reliable, robust, and rapid method for assessing organelle proximity. IP3R–VDAC PLA was used to quantify MAMs (Tubbs and Rieusset, 2016), while Mi–LD and ER–LD contacts were assessed using PTPIP51–PLIN1 and VAPB–PLIN1 PLA, respectively. Throughout the following analyses, PLA is used to assess proximity between selected protein pairs, providing a proxy for either established contact sites (IP3R–VDAC) or close apposition between organelles (PTPIP51–PLIN1 and VAPB–PLIN1). However, in initial optimization experiments using oleate stimulation in 3T3-L1 differentiated adipocytes, we validated our approach by using two distinct PLA pairs for each contact type. These independent measurements yielded consistent results, which were further supported by TEM analyses (Appendix Fig. S2). In the fed state, all three contact types were reduced in seipin-deficient mice compared with controls (Fig. 1J–M; Appendix Fig. S3A,B), consistent with the TEM data obtained under the same nutritional conditions (Fig. 1A–D). Comparison between fasted and fed states revealed that feeding reduced total MAMs (IP3R–VDAC) but increased ER–LD (VAPB–PLIN1) and Mi–LD (PTPIP51–PLIN1) contacts in control mice. This nutrient-driven remodeling of contact sites was abolished in iATSKO mice, except for ER–LDs, although the extent of the decrease was smaller than in controls (Fig. 1M). Of note, we assessed LD area and found that, while feeding increased LD area, it was not affected by Seipin knockout at this stage (15 days post deletion), as determined by PLIN1 fluorescence signal quantification (Fig. EV1J).

To assess LD property changes, we performed a gentle LD isolation (3000 g) to preserve PDM and MAM-LD together with LD, in the lipid-floating fraction. Proteomic analysis detected mitochondrial markers (such as PTPIP51/Rmdn3) but no genotype-dependent proteome changes (Appendix Fig. S3C). Lipidomic analysis, however, showed strong iATSKO/control separation (Fig. 1N) driven by decreased triglycerides (TG) and increased diglycerides (DG), suggesting defective TG synthesis/targeting (Fig. 1O). Predictive analysis confirmed TG decrease and DG increase as predictors of genotype (Appendix Fig. S3D), while LD area remained unchanged (Fig. EV1J).

Collectively, these results show that Seipin deficiency impairs nutritional remodeling of MCS, particularly MAM-LD structure, and is associated with reduced LD TG storage and DG accumulation.

### Seipin deficiency alters MAM–LD contacts and disrupts TG flux toward lipid droplets

**Figure EV2:**
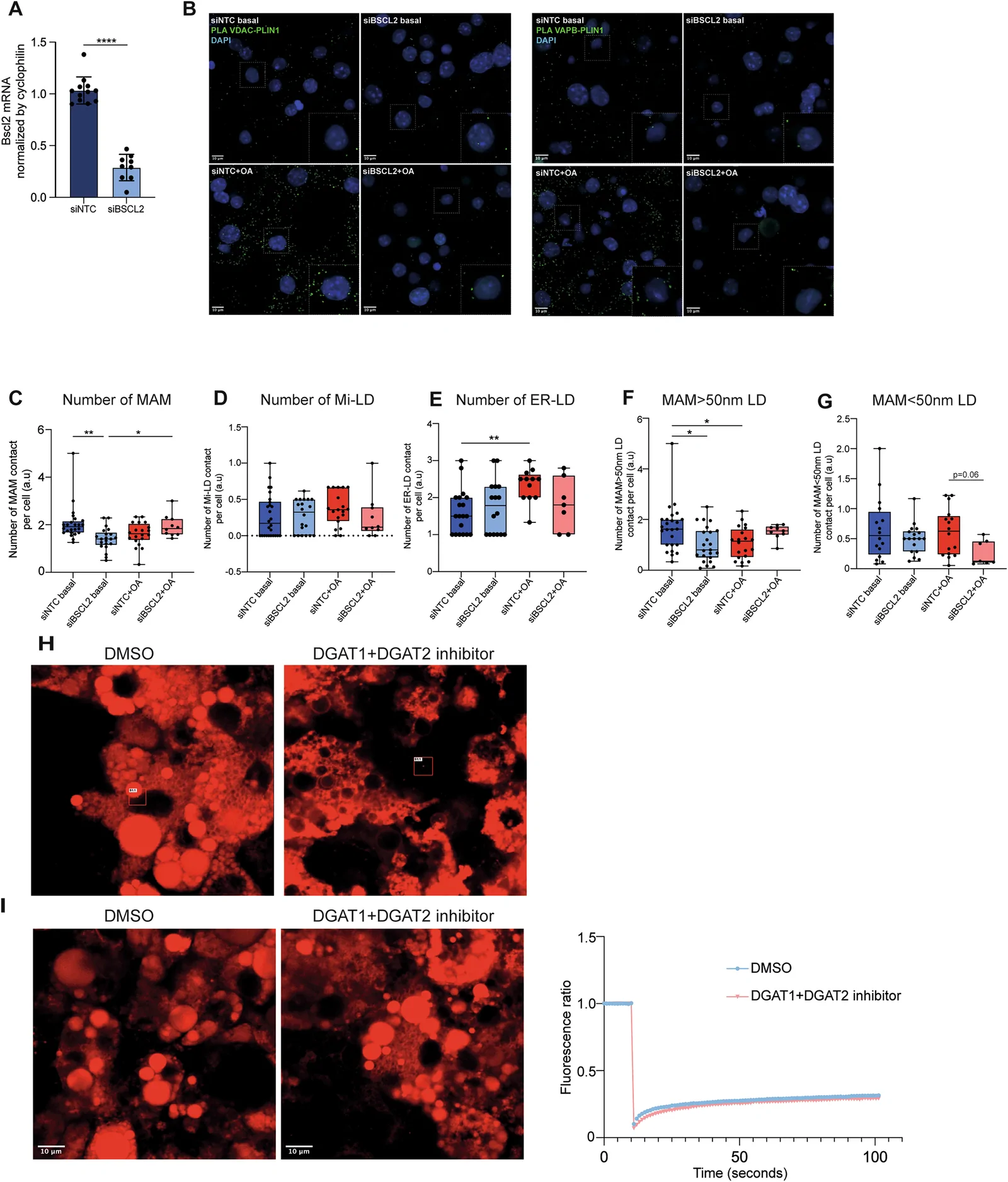
Seipin depletion alters organelle contacts and lipid flux in adipocytes. (**A**) *Bscl2* mRNA expression was quantified by RT-qPCR in 3T3-L1 adipocytes transfected with control (siNTC) or *BSCL2* siRNA (siBSCL2). (**B**) Representative PLA images of VDAC/PLIN1 and VAPB/PLIN1 in control and *BSCL2*-depleted adipocytes under basal or oleate-loaded conditions. (**C**–**G**) TEM analyses were performed to quantify the number of MAMs (**C**), Mi–LD contacts (**D**), ER–LD contacts (**E**), MAM–CM (>50 nm from LDs) (**F**), and MAM–LD (<50 nm from LDs) (**G**). (**H**) 3T3-L1 adipocytes were incubated for 6 h with BODIPY-C12 and treated with DMSO or DGAT1/2 inhibitors. (**I**) FRAP analysis was performed in adipocytes treated with DMSO or DGAT1/2 inhibitors added immediately before acquisition. Data information: (**A**) Bars represent mean ± SEM; triplicate measurements from three independent experiments. (**C**–**G**) Box plots show the median (center line), the 25th and 75th percentiles (box), the minimum and maximum values (whiskers), and individual data points; measurements were obtained from >10 cell profiles per condition. Statistical analysis: Mann–Whitney test (**A**); two-way ANOVA (**C**–**G**). Exact adjusted *P* values from multiple comparisons test: siNTC basal vs. siBSCL2 basal, *P* = 0.0057; siBSCL2 basal vs. siBSCL2 + OA, *P* = 0.0436 (**C**). siNTC basal vs. siNTC + OA, *P* = 0.0145 (**E**). siNTC basal vs. siBSCL2 basal, *P* = 0.0209; siNTC basal vs. siNTC + OA, *P* = 0.0447 (**F**). All other significant comparisons, *P* < 0.001. *p < 0.05, **p < 0.01, ***p < 0.001, ****p < 0.0001.

**Figure 2:**
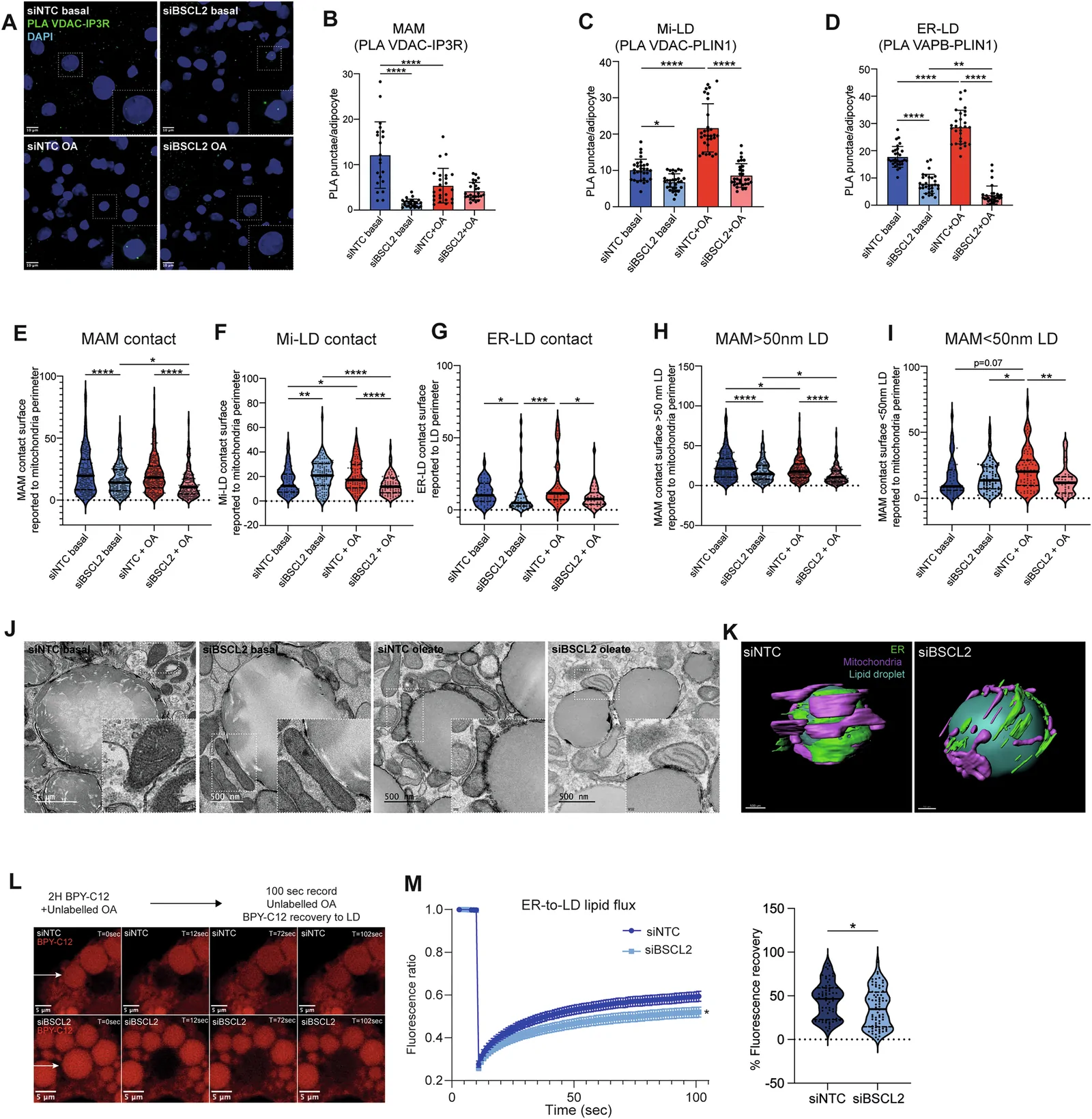
Seipin deficiency alters ER–lipid droplet lipid flux in adipocytes. (**A**–**D**) 3T3-L1 adipocytes differentiated for 7 days were reverse-transfected with the indicated siRNAs. After 72 h, cells were incubated in low-glucose medium with or without oleic acid (OA) for 6 h. PLA was performed to quantify MAMs (VDAC/IP3R; **A**, **B**), Mi–LD contacts (VDAC/PLIN1; **C**), and ER–LD contacts (VAPB/PLIN1; **D**). (**E**–**J**) TEM was used to quantify the contact surface areas of MAMs, Mi–LD, and ER–LD interfaces (**E**–**G**). MAM subpopulations were quantified according to their proximity to LDs: MAM–LD (<50 nm from LDs) and MAM–CM (>50 nm from LDs) (**H**, **I**). Representative TEM images are shown (**J**). (**K**) Representative 3D FIB-SEM reconstructions of differentiated 3T3-L1 adipocytes transfected with the indicated siRNAs, showing segmentation of ER (green), mitochondria (magenta), and LDs (cyan). (**L**,** M**) Fluorescence recovery after photobleaching (FRAP) analysis was performed to assess lipid droplet fluorescence recovery. Representative FRAP images are shown in (**L**), and fluorescence recovery kinetics with quantification are shown in (**M**). Data information: (**B**–**D**) Bars represent mean ± SD; two independent biological experiments, with 15 fields acquired per condition per experiment and ~50 cells analyzed per field (~1500 cells per condition). (**E**–**I**) Violin plots show the distribution of quantified contacts, with each dot representing one individual contact and the median indicated by the central line. Measurements were obtained from >10 cell profiles per condition. (**M**) Violin plots show fluorescence recovery (%), with each dot representing one cell; median and quartiles are indicated. *n* > 80 cells per condition from three independent experiments. Statistical analysis: two-way ANOVA (**A**–**I**); Mann–Whitney test (**M**). Exact adjusted *P* values for two-way ANOVA: siNTC basal vs. siBSCL2 basal, *P* = 0.013 (**C**); siBSCL2 basal vs. siBSCL2 + OA, *P* = 0.3409 (**D**). Data were analyzed by one-way ANOVA followed by Tukey’s multiple comparisons test. siBSCL2 basal vs. siNTC +  OA, *P* = 0.0073; siBSCL2 basal vs. siBSCL2 + OA, *P* = 0.0436 (**E**). siNTC basal vs. siBSCL2 basal, *P* = 0.0074; siNTC basal vs. siNTC + OA, *P* = 0.0277 (**F**). siNTC basal vs. siBSCL2 basal, *P* = 0.0208; siNTC + OA vs. siBSCL2 + OA, *P* = 0.0202 (**G**). siNTC basal vs. siNTC + OA, *P* = 0.0125; siBSCL2 basal vs. siBSCL2 + OA, *P* = 0.0227 (**H**). siBSCL2 basal vs. siNTC + OA, *P* = 0.0290; siNTC + OA vs. siBSCL2 + OA, *P* = 0.0050 (**I**). Two-way ANOVA: interaction (time × group), *P* = 0.0301 (**M**) Exact *P* value from a two-tailed Mann–Whitney test: *P* = 0.0212 (**M**). All other significant comparisons, *P* < 0.001. *P* < 0.0001.*p < 0.05, **p < 0.01, ***p < 0.001, ****p < 0.0001. Source data are available online for this figure.

Seipin prevents TG from escaping LDs (Salo et al, 2019) and localizes at MAMs. Thus, the reduction in organelle contacts in Seipin-deficient conditions, particularly MAM–LD contacts in adipocytes, led us to investigate whether Seipin, or Seipin together with MAM-LD, is required for efficient lipid storage into LDs. To address this, we used 3T3-L1 adipocytes transfected with either non-targeting control siRNA (siNTC) or BSCL2 siRNA (siBSCL2), resulting in a 75% Bscl2 mRNA decrease in 72 h (Fig. EV2A). PLA analysis confirmed that Seipin knockdown (siBSCL2) reproduced the MCS defects observed in iATSKO mice, showing decreased ER–Mi, ER–LD, and Mi–LD contacts in basal conditions, and no adaptation under oleic acid (OA) loading (Figs. 2A–D and EV2B). Importantly, these changes in contact sites occur while the number and area of mitochondria, LDs, and ER remain unaffected by siBSCL2 (Appendix Fig. S4). TEM confirmed the same overall trend while providing a more detailed description of organelle organization. MAM surface (Fig. 2E) and MAM number (Fig. EV2C) were decreased in Seipin knockdown cells under basal conditions. Unexpectedly, OA treatment induced a slight increase in MAM number compared with the basal state, but only in Seipin-deficient cells. OA increased the Mi–LD contact surface in control cells, whereas in seipin-deficient cells, these contacts were already elevated under basal conditions but decreased upon lipid loading, revealing abnormal remodeling (Fig. 2F). The ER–LD contact surface was reduced under both basal and OA conditions in Seipin-deficient cells compared with control adipocytes (Fig. 2G), and OA failed to increase their number in Seipin-deficient cells (Fig. EV2E). In control cells, lipid loading reduced the surface area of the MAMs distant from the LD (i.e., MAM-CM; Fig. 2H), but concomitantly increased MAM-LD surface area, a remodeling response that was absent in siBSCL2-treated adipocytes (Fig. 2I,J). Similarly, the number of MAM-CM contacts decreased in control cells under OA treatment, but not in seipin-deficient cells (Fig. EV2F), while MAM-LD contacts showed a non-significant trend toward a decrease in seipin-deficient cells under OA treatment (Fig. EV2G). FIB-SEM imaging combined with organelle segmentation further revealed that, in control cells, the three organelles are tightly arranged, with ER–mitochondria–LD and mitochondria–ER–LD configurations being frequently observed, supporting the existence of stable tripartite contacts. In contrast, in Seipin-deficient cells, mitochondria and the ER no longer closely surround LDs, indicating a loss of tight spatial organization between these organelles (Fig. 2K; Movies EV3 and EV4).

Next, we evaluated neutral lipid incorporation into LDs using fluorescence recovery after photobleaching (FRAP). Cells were incubated with the fluorescent fatty acid BODIPY-C12 (BPY-C12) for two hours, during which it is metabolized and esterified into neutral lipids, predominantly TG, that accumulate in LDs. After 2 h, photobleaching was applied to LDs and fluorescence recovery was monitored. Seipin-deficient cells exhibited markedly reduced fluorescence recovery, indicating impaired TG transfer (Fig. 2L,M). Of note, inhibition of triglyceride synthesis enzymes (DGAT1 and DGAT2) during the BODIPY-C12 fluorescent fatty acid loading phase altered the cellular labeling pattern, preventing large and medium-sized LDs from becoming fluorescent. This confirms that incorporation of the probe depends on triglyceride synthesis during this step (Fig. EV2H). In contrast, inhibition of DGAT1/2 when applied only during the FRAP assay did not affect fluorescence recovery (Fig. EV2I). These findings indicate that the observed fluorescence recovery does not depend on ongoing triglyceride synthesis during the assay itself, but rather on prior incorporation of the fluorescent fatty acid into triglycerides during the loading phase.

Altogether, these results show that Seipin deficiency alters organelle remodeling under lipid conditions, specifically decreasing MAM–LD contacts, and that this structural impairment is associated with a functional alteration in ER-to-LD TG flux.

### Seipin regulates LD lipid storage by modulating MAM–LD contacts in a calcium-dependent manner

**Figure EV3:**
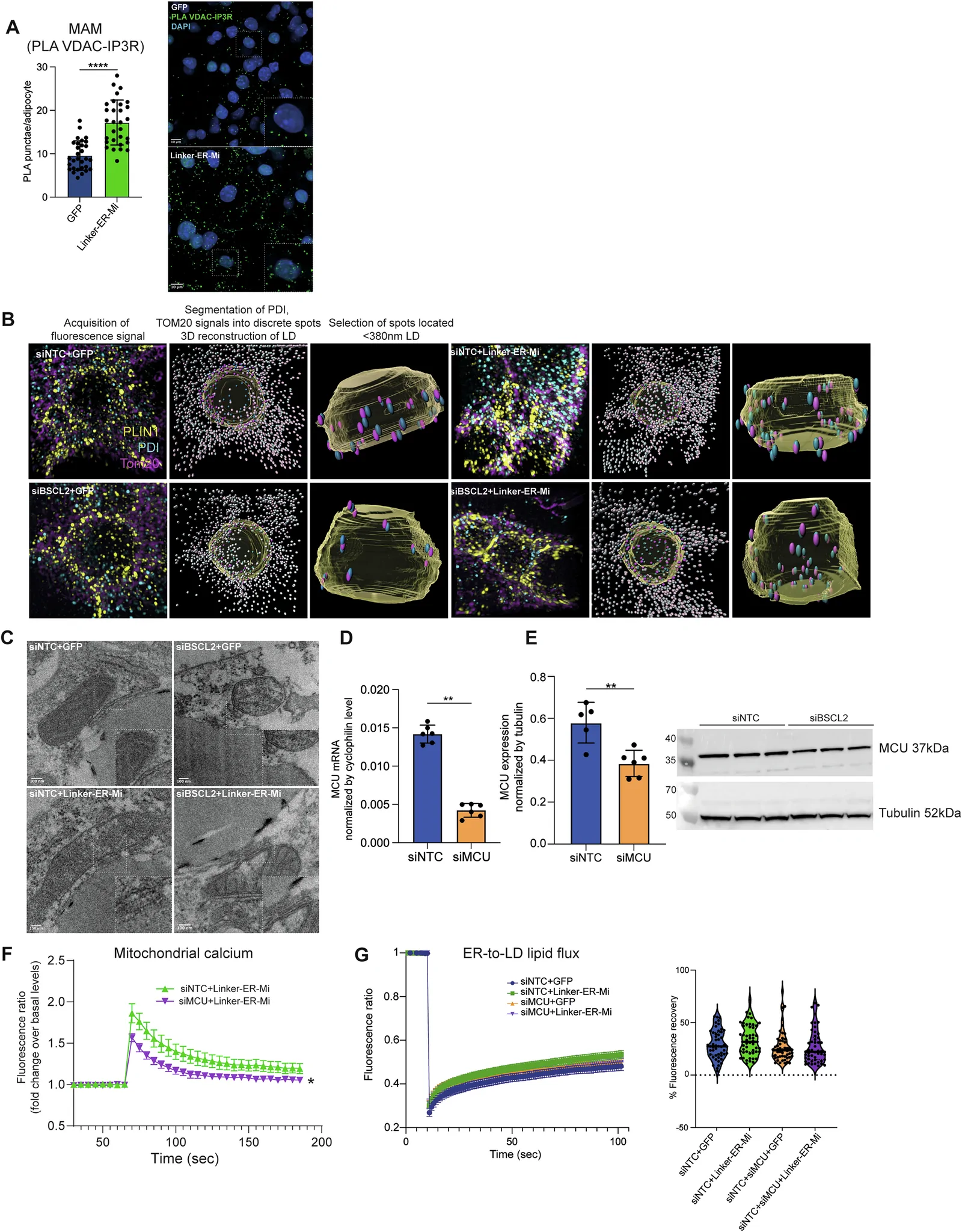
ER–mitochondria tethering and mitochondrial calcium handling in adipocytes. (**A**) 3T3-L1 adipocytes were infected with adenoviruses expressing GFP or Linker-ER-Mi, and ER–mitochondria contacts were quantified by VDAC/IP3R PLA. (**B**) Structured illumination microscopy (SIM) and Imaris analysis workflow used to quantify ER–mitochondria contacts in proximity to lipid droplets. Selection of spots representation are reuse of Fig. 3A. (**C**) Representative TEM images of 3T3-L1 adipocytes transfected with control or *BSCL2* siRNA and infected with GFP or Linker-ER-Mi adenoviruses. (**D**) *MCU* mRNA expression was quantified by RT-qPCR following siRNA-mediated depletion. (**E**) Representative immunoblots of MCU and actin with corresponding quantification. (**F**) Mitochondrial calcium uptake was monitored using caged IP₃ photorelease and Rhod-2 fluorescence. (**G**) FRAP analysis was performed to assess fluorescence recovery kinetics. Data information: (**A**) Bars represent mean ± SD; two independent biological experiments, with 15 fields acquired per condition per experiment and ~50 cells analyzed per field (~1500 cells per condition). (**D**) Bars represent mean ± SEM; triplicate measurements from two independent experiments. (**E**) Bars represent mean ± SD; triplicate measurements from two independent experiments. (**F**) *n* > 60 cells per condition from 3 independent experiments. (**G**) Violin plots show fluorescence recovery (%), with each dot representing one cell; median and quartiles are indicated; *n* > 45 cells per condition from three independent experiments. Statistical analysis: Mann–Whitney test (**A**, **D**, **E**); two-way ANOVA (**F**); one-way ANOVA (**G**). Exact *P* values from two-tailed Mann–Whitney tests: *P* = 0.0022 (**D**) and *P* = 0.0087 (**E**). All other significant comparisons, *P* < 0.001. *p < 0.05, **p < 0.01, ***p < 0.001, ****p < 0.0001.

**Figure 3:**
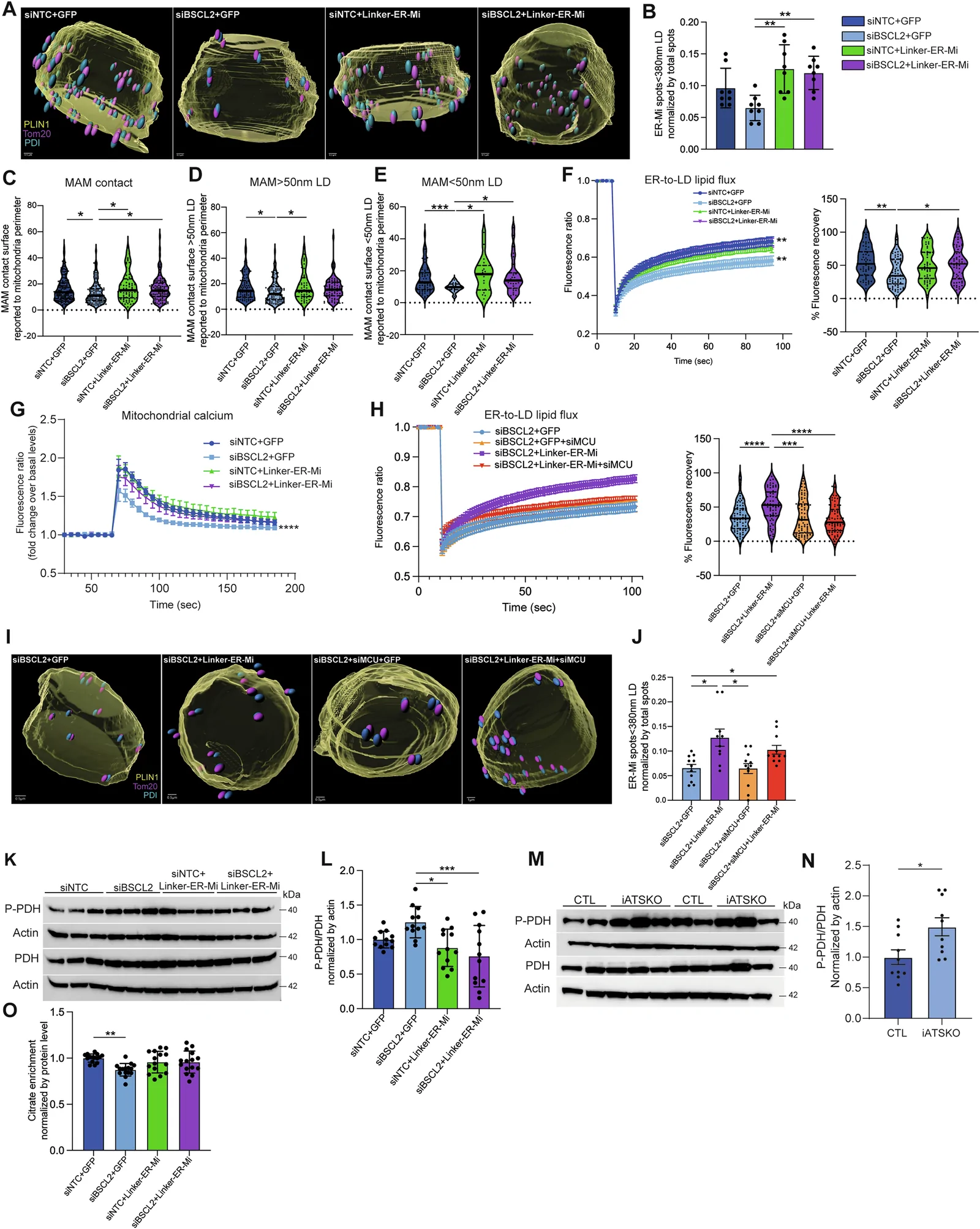
MAM–LD organization regulates lipid flux through calcium-dependent mechanisms. (**A**,** B**) 3T3-L1 adipocytes were reverse-transfected with the indicated siRNAs and infected with adenoviruses expressing GFP or Linker-ER-Mi. Structured illumination microscopy (SIM) was used to visualize mitochondria (Tom20), ER (PDI), and LDs (PLIN1). Representative segmented images are shown in (**A**). ER–mitochondria contacts located within 380 nm of LDs were quantified using the Imaris “Find Spots Close to Surface” plugin (**B**). (**C**–**E**) TEM was used to quantify total MAM contact surface area (**C**), MAM–CM (>50 nm from LDs) (**D**), and MAM–LD (<50 nm from LDs) (**E**). (**F**) FRAP fluorescence recovery kinetics. (**G**) Mitochondrial calcium uptake was monitored using caged IP₃ photorelease and Rhod-2 fluorescence. After 60 s of baseline acquisition, IP₃ release was induced by UV flash photolysis. Fluorescence is shown relative to baseline. (**H**) FRAP fluorescence recovery analysis in 3T3-L1 adipocytes transfected with the indicated siRNAs (siNTC, siBSCL2, and siMCU) and infected with GFP or Linker-ER-Mi. (**I**,** J**) SIM imaging and Imaris analysis of ER–mitochondria contacts in proximity to LDs. Representative segmented images are shown in (**I**), with quantification in (**J**). (**K**,** L**) Representative immunoblots of phospho-PDH (P-PDH), total PDH, and actin in 3T3-L1 adipocytes (**K**), with quantification shown in (**L**). (**M**,** N**) Representative immunoblots of phospho-PDH (P-PDH), total PDH, and actin from inguinal adipose tissue lysates of Control and iATSKO mice (**M**), with quantification shown in (**N**). (**O**) Fractional enrichment of [¹³C]-glucose-derived citrate analyzed by LC-HRMS. Data information: (**B**) Bars represent mean ± SD; *n* > 8 cells per condition from two independent biological replicates. (**C**–**E**) Violin plots show the distribution of quantified contacts, with each dot representing one individual contact and the median indicated by the central line. Measurements were obtained from >5 cell profiles per condition. (**F**, **H**) Violin plots show fluorescence recovery (%), with each dot representing one cell; median and quartiles are indicated. *n* > 80 cells per condition from three independent experiments. (**G**) *n* > 60 cells per condition from three independent biological replicates. (**J**) Bars represent mean ± SEM; *n* = 10 cells per condition from three independent experiments. (**L**) Mean ± SD; *n* = 3 replicates from four independent experiments. (**N**) Mean ± SEM; *n* = 8 mice per genotype. Samples were analyzed in two independent immunoblot experiments. (**O**) Bars represent mean ± SEM; triplicate measurements from three independent experiments. Statistical analysis: two-way ANOVA (**B**, **C**–**E**, **G**); one-way ANOVA (**F**, **H**, **J**, **L**, **O**); Mann–Whitney test (**N**). Exact adjusted *P* values from Dunn’s multiple comparisons test: siBSCL2 vs. siNTC + LINKER, *P* = 0.0042; siBSCL2 vs. siBSCL2 + LINKER, *P* = 0.0068 (**B**). siNTC + GFP vs. siBSCL2 + GFP, *P* = 0.0350; siBSCL2 + GFP vs. siNTC + Linker-ER-Mi, *P* = 0.0133; siBSCL2 + GFP vs. siBSCL2  + Linker-ER-Mi, *P* = 0.0224 (**C**). siNTC + GFP vs. siBSCL2 + GFP, *P* = 0.0478; siBSCL2 + GFP vs. siNTC + Linker-ER-Mi, *P* = 0.0410 (**D**). siBSCL2 + GFP vs. siNTC + Linker-ER-Mi, *P* = 0.0457; siBSCL2 + GFP vs. siBSCL2 + Linker-ER-Mi, *P* = 0.0163 (**E**). Two-way repeated-measures ANOVA: time × group interaction, *P* = 0.0021 (**F**, left) and *P* = 0.0172 (**G**). Exact adjusted *P* values from Tukey’s multiple comparisons test: siNTC + GFP vs. siBSCL2 + GFP, *P* = 0.0059; siBSCL2 + GFP vs. siBSCL2 + Linker-ER-Mi, *P* = 0.0241 (F, right); siBSCL2 + LINKER5 vs. siBSCL2 + MCU + GFP, *P* = 0.0001 (**H**); siBSCL2 vs. siBSCL2 + L5, *P* = 0.0376; siBSCL2 vs. siBSCL2 + MCU + L5, *P* = 0.0299; siBSCL2 + L5 vs. siBSCL2 + MCU, *P* = 0.0397 (**J**); siBSCL2 + GFP vs. siNTC + Linker-ER-Mi, *P* = 0.0152 (**L**). Two-tailed Mann–Whitney test: *P* = 0.0232 (**N**). Kruskal–Wallis test: *P* = 0.0037 (**O**). All other significant comparisons, *P* < 0.001. *p < 0.05, **p < 0.01, ***p < 0.001, ****p < 0.0001. Source data are available online for this figure.

The impairment of ER-to-LD TG flux in Seipin-deficient adipocytes is consistent with previous reports showing similar results in non-adipocyte cells (Salo et al, 2016; Wang et al, 2016). However, whether this defect depends on MAMs remains unknown. To test whether MAM–LD contacts directly mediate ER-to-LD lipid transfer, we genetically modulated contact formation by overexpressing Linker-ER-Mi, an artificial ER–mitochondria tether designed to promote MAM assembly (Csordas et al, 2006). First, we validated, using PLA, that expression of the Linker-ER-Mi increased MAM abundance (VDAC-IP3R) in 3T3-L1 adipocytes (Fig. EV3A). Next, we expressed the Linker-ER-Mi in Seipin-deficient adipocytes, and we used Structured illumination microscopy (SIM) to assess the effect on MAM-LD. SIM analysis with immunolabeling for mitochondria (Tom20), ER (PDI), and LDs (PLIN1), followed by 3D analysis using a 380 nm proximity threshold corresponding to an estimation of the spatial resolution limit of the technique (Guyard et al, 2022) (Fig. EV3B) showed that Linker-ER-Mi increases the spatial proximity between ER, mitochondria, and LDs, in the context of Seipin deficiency (Fig. 3A,B). TEM further confirmed that Linker-ER-Mi expression reestablished MAM structures, particularly MAM-LD contacts (Figs. 3C–E and EV3C). Repeating the FRAP assay under these conditions demonstrated that Linker-ER-Mi fully rescued the ER-to-LD lipid flux defect in siBSCL2-treated adipocytes (Fig. 3F). Of note, the Linker-ER-Mi has no effect on fluorescence recovery in siNTC-treated cells.

Given the known role of MAMs in ER-to-mitochondria calcium signaling, we next performed dynamic calcium imaging. In 3T3-L1 adipocytes, using Rhod-2 to monitor mitochondrial calcium uptake following IP3-induced ER calcium release, we found that Seipin deficiency significantly reduced the calcium peak, and this defect was reversed by Linker-ER-Mi expression (Fig. 3G). These findings raise the possibility that calcium exchange may contribute to the corrective effect of Linker-ER-Mi on ER-to-LD lipid transfer. To test whether the effect of Linker-ER-Mi on calcium transfer is required for its ability to rescue lipid flux in Seipin-deficient cells, we blocked mitochondrial calcium uptake by silencing the mitochondrial calcium uniporter (MCU) using siRNA. We confirmed that siMCU effectively reduced MCU mRNA and protein levels (Fig. EV3D,E) and abolished the Linker-ER-Mi-induced rescue in mitochondrial calcium import (Fig. EV3F). We then performed FRAP experiments and found that inhibition of mitochondrial calcium uptake by MCU knockdown abolished the rescue of ER-to-LD lipid transfer normally observed with Linker-ER-Mi (Fig. 3H). In control cells, MCU knockdown showed a mild trend toward reduced fluorescence recovery, although this did not reach statistical significance (Fig. EV3G). Importantly, MCU knockdown did not alter the structural proximity between the three organelles (ER, mitochondria, and LDs), as shown in Fig. 3I,J, highlighting the critical role of calcium in regulating ER-to-LD lipid flux. Given the importance of calcium in mitochondrial function—particularly in activating enzymes of the TCA cycle—we next assessed the phosphorylation state of pyruvate dehydrogenase (PDH), a key enzyme that converts pyruvate into citrate. While PDH phosphorylation, which inhibits its activity, is promoted under low-calcium conditions, we observed an elevation of PDH phosphorylation levels in Seipin-deficient adipocytes that returned to baseline upon Linker-ER-Mi expression (Fig. 3K,L; Appendix Fig. S5A). Here, PDH phosphorylation was used as an indirect readout of mitochondrial calcium levels, complementing our direct measurements (Fig. 3G). While direct measurements of mitochondrial calcium levels were technically not feasible in the adipose tissue of iATSKO mice, PDH phosphorylation levels were increased (Fig. 3M,N), supporting the hypothesis that impaired mitochondrial calcium import is a hallmark of Seipin deficiency. To ensure that variations in PDH levels were not due to differences in total mitochondrial mass, we also quantified VDAC1 expression as a mitochondrial marker and observed no differences (Appendix Fig. S5B). Finally, by tracing ^13^C-labeled glucose metabolism, we found that citrate production was impaired in Seipin-deficient cells and that this defect was corrected by Linker-ER-Mi expression (Fig. 3O).

Altogether, our findings show that MAM-LD reinforcement is sufficient to restore ER-to-LD TG transfer in Seipin-deficient cells, but that this corrective effect depends on ER to mitochondria-calcium-flux.

### Mapping MCS during adipogenesis and under distinct nutritional conditions

Our work on Seipin-deficient adipocytes demonstrates that alterations in MCS involving the ER, LDs, and mitochondria and especially the tripartite MAM-LD, are associated with severe adipocyte dysfunction. Because these contact sites have been poorly studied in adipocytes, we characterized these interactions during two key physiological states of adipocyte function: adipogenesis and the response to nutrient availability in differentiated cells.

**Figure 4:**
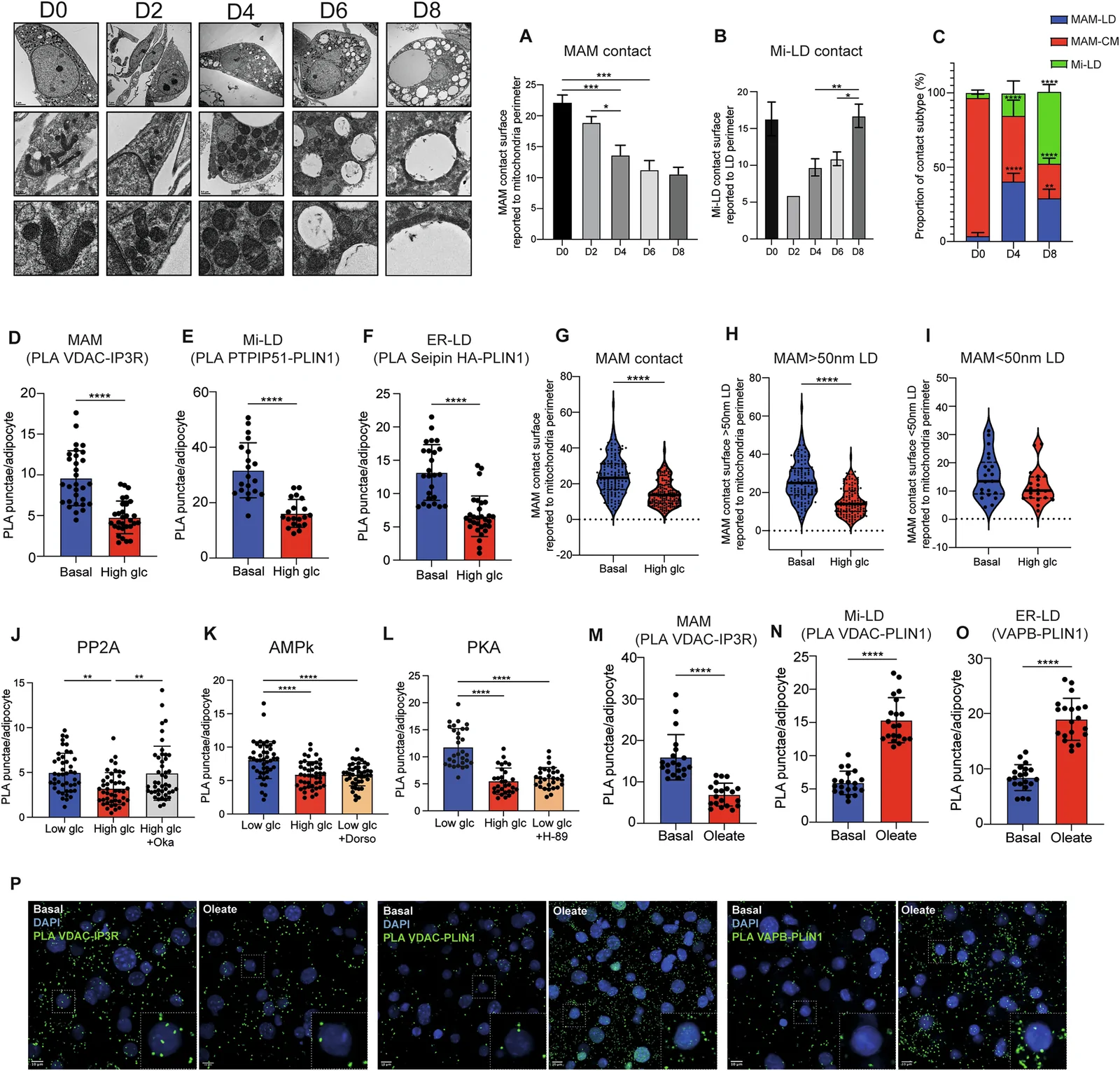
Mapping membrane contact-site dynamics during adipogenesis and under distinct nutritional conditions. (**A**–**C**) 3T3-L1 differentiation was initiated at day 0 by the addition of the adipogenic cocktail (see Materials and Methods). TEM analyses were performed at the indicated time points after induction of differentiation to quantify MAM and mitochondria–lipid droplet (Mi–LD) contact surfaces (**A**, **B**), as well as the relative distribution of MAM subpopulations and Mi–LD contacts (**C**). (**D**–**F**) 3T3-L1 adipocytes differentiated for 7 days were incubated for 6 h in low-glucose (5 mM) medium with or without high glucose (25 mM). PLA assays were performed to quantify MAMs (VDAC/IP3R; **D**), Mi–LD contacts (PTPIP51/PLIN1; **E**), and ER–LD contacts (Seipin-HA/PLIN1; **F**). (**G**–**I**) TEM analyses were performed to quantify total MAM contact surface (**G**), MAM–CM (>50 nm from LDs) (**H**), and MAM–LD (<50 nm from LDs) (**I**). (**J**–**L**) Cells were incubated in low- or high-glucose medium and treated with okadaic acid (Oka, 10 nM), dorsomorphin (Dorso, 5 µM), or H-89 (10 µM), and PLA assays were performed to quantify ER–mitochondria contacts (VDAC/IP3R; **J**–**L**). (**M**–**P**) 3T3-L1 adipocytes differentiated for 7 days were incubated for 6 h in low-glucose (5 mM) medium with or without oleic acid (700 µM). PLA assays were performed to quantify MAMs (VDAC/IP3R; **M**), Mi–LD contacts (PTPIP51/PLIN1; **N**), and ER–LD contacts (Seipin-HA/PLIN1; **O**). Representative PLA images are shown in (**P**). Data information: (**A**–**C**) Bars represent mean ± SEM; *n* = 3 cells per condition from independent biological replicates. (**D**–**F**, **M**–**O**) Bars represent mean ± SD; 2 or 3 independent biological experiments, with 15 fields acquired per condition per experiment and ~50 cells analyzed per field (~1500 cells per condition). (**G**–**I**) Violin plots show the distribution of quantified contacts, with each dot representing one individual contact and the median indicated by the central line; measurements were obtained from >5 cell profiles per condition. (**J**–**L**) Bars represent mean ± SEM; three independent biological experiments, with 15 fields acquired per condition per experiment and ~50 cells analyzed per field (~1500 cells per condition). Statistical analysis: one-way ANOVA (**A**–**C**, **D**–**F**, **J**–**L**, **M**–**O**); two-way ANOVA (**G**–**I**). Exact adjusted *P* values from. Tukey’s multiple comparisons test: D0 vs. D4, *P* = 0.0001; D2 vs. D4, *P* = 0.0488; D2 vs. D6, *P* = 0.0235 (**A**); D4 vs. D8, *P* = 0.0045; D6 vs. D8, *P* = 0.0172 (**B**). Exact adjusted *P* values from Tukey’s multiple comparisons test: low glucose vs. high glucose, *P* = 0.0017; high glucose vs. high glucose + okadaic acid, *P* = 0.0025. All other significant comparisons, *P* < 0.001 *p < 0.05, **p < 0.01, ***p < 0.001, ****p < 0.0001. Source data are available online for this figure.

To assess MCS during adipogenesis in 3T3-L1 cells, we performed transmission electron microscopy (TEM), which revealed that the appearance of LDs at day 4 post-induction of differentiation was accompanied by a redistribution of mitochondria. From this stage onward, the images showed PDM surrounding the LDs. Quantification of the MCSs showed an increase in Mi–LD contacts and a concomitant decrease in total-MAMs (Fig. 4A,B). To further clarify the specific implications of MAM-LD in this process, we segregated the quantification of MAM-CM, MAM-LD and Mi–LD. We confirmed a decrease of MAM-CM during adipocyte differentiation, while MAM-LD were increased at day 4 of induction, highlighting specific regulation of this MAM subtype during adipocyte differentiation (Fig. 4C).

**Figure EV4:**
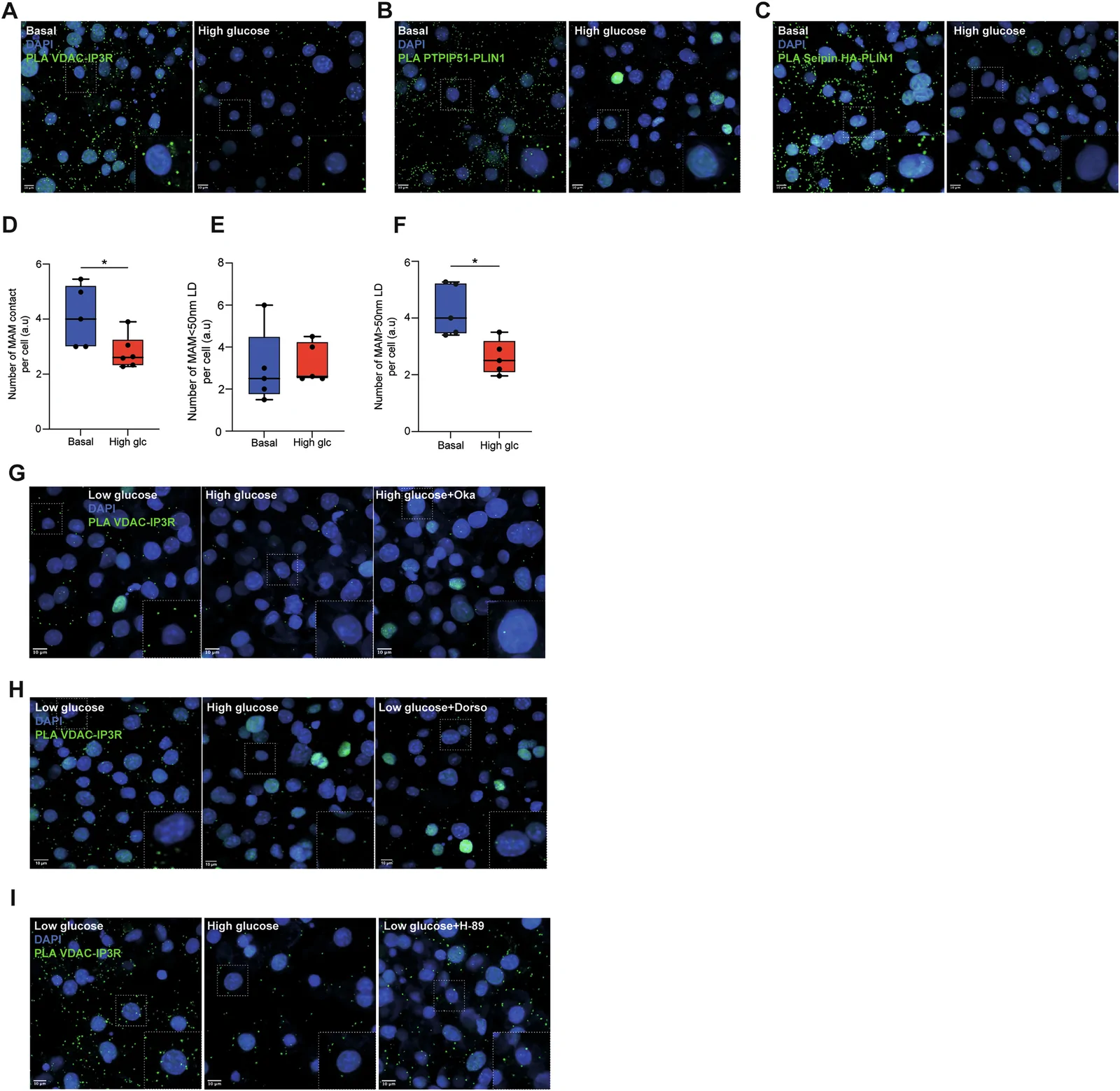
Nutrient availability regulates membrane contact-site organization in adipocytes. (**A**–**C**) Representative PLA images of ER–mitochondria contacts (VDAC/IP3R; **A**), Mi–LD contacts (PTPIP51/PLIN1; **B**), and ER–LD contacts (Seipin-HA/PLIN1; **C**) in adipocytes cultured under basal (6 h, 5 mM glucose) or high-glucose (6 h, 25 mM glucose) conditions. (**D**–**F**) TEM analyses were performed to quantify the number of MAMs (**D**), MAM–LD (<50 nm from LDs) (**E**), and MAM–CM (>50 nm from LDs) (**F**) per cell. (**G**–**I**) Representative PLA images of MAMs (VDAC/IP3R) in adipocytes cultured under low-glucose conditions alone or treated with dorsomorphin (5 µM) or H-89 (10 µM). Data information: (**D**–**F**) Box plots show the median (center line), the 25th and 75th percentiles (box), the minimum and maximum values (whiskers), and individual data points. Measurements were obtained from >5 cell profiles per condition. Statistical analysis: Mann–Whitney tests. Exact *P* values from two-tailed Mann–Whitney tests: *P* = 0.0455 (**D**) and *P* = 0.0238 (**F**). *p < 0.05.

Then, we intended to decipher the MCS remodeling in response to nutrient treatment. Using PLA, we quantified MAMs through IP3R–VDAC interactions, Mi–LD contacts via PTPIP51–PLIN1, and ER–LD contacts via Seipin-HA–PLIN1. High glucose (25 mM) exposure reduced all three types of contact sites (Figs. 4D–F and EV4A–C). TEM analysis revealed that only the decreases in MAM-CM number and surfaces reached statistical significance, whereas the reduction in MAM-LD did not (Figs. 4G–I and EV4D–F). The glucose-induced decrease in MAMs was abolished by treatment with okadaic acid (Oka), an inhibitor of protein phosphatase 2A (PP2A), which is known to be activated by xylulose-5-phosphate, a product of glucose metabolism (Fig. 4J). Conversely, the increase in MAMs observed under low-glucose (5 mM) conditions was blunted by dorsomorphin and H-89, inhibitors of AMPK and PKA, respectively—two signaling pathways typically activated in response to nutrient deprivation (Figs. 4K,L and EV4G,H). Interestingly, in fully differentiated 3T3-L1 adipocytes, treatment with oleic acid (OA) led to a reduction in MAMs, while ER–LD and Mi–LD contact sites were significantly increased (Fig. 4M–P). As we already demonstrated in Fig. 2, OA decreased MAM-CM while promoting MAM-LD formation (Fig. 2H,I), leading us to conclude that glucose and lipids differentially regulate MCS remodeling in adipocytes.

Together, these data strongly suggest that MCS remodeling is a key determinant of adipocytes’ metabolic flexibility and that MAM-LD plays a key role in lipid handling.

### MCS nutritional remodeling is abolished in the adipose tissue of diet-induced obese mice

**Figure 5:**
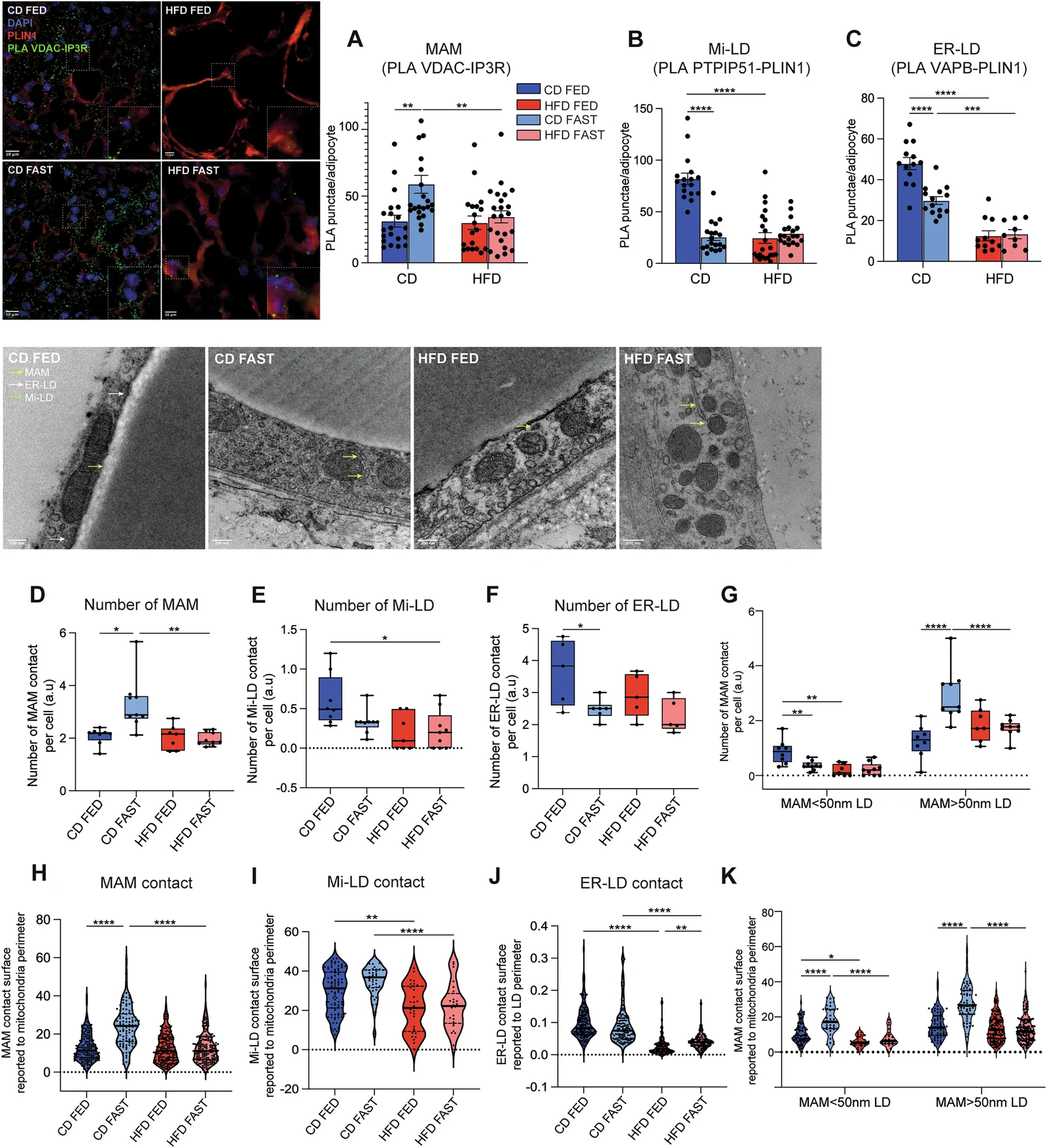
Membrane contact sites are altered in adipose tissue of obese mice. (**A**–**C**) Cryopreserved gonadal adipose tissue was collected from mice fed either a chow diet or a high-fat diet for 3 months, in the fed or 18 h fasted state. PLA assays were performed to quantify ER–mitochondria contacts (VDAC/IP3R; **A**), Mi–LD contacts (PTPIP51/PLIN1; **B**), and ER–LD contacts (VAPB/PLIN1; **C**). (**D**–**G**) Gonadal adipose tissue from the same experimental groups was analyzed by TEM to quantify the number of MAMs (**D**), Mi–LD contacts (**E**), ER–LD contacts (**F**), and MAM subpopulations according to lipid droplet proximity (**G**). (**H**–**K**) TEM analyses were used to quantify the contact surface areas of MAMs (**H**), Mi–LD contacts (**I**), ER–LD contacts (**J**), and MAM subpopulations according to lipid droplet proximity (**K**). Data information: (**A**–**C**) Bars represent mean ± SEM; *n* = 5 mice per group, >100 cells per mouse. (**D**–**G**) Box plots show the median (center line), the 25th and 75th percentiles (box), the minimum and maximum values (whiskers), and individual data points. (**H**–**K**) Violin plots show the distribution of quantified contacts, with each dot representing one individual contact and the median indicated by the central line. TEM measurements were obtained from three mice per group, with five cells analyzed per mouse. Statistical analysis: two-way ANOVA (**A**–**C**); one-way ANOVA (**D**–**K**). Exact adjusted *P* values from Tukey’s multiple comparisons test: CD fed vs. CD fasted, *P* = 0.0026; CD fasted vs. HFD fed, *P* = 0.0018; CD fasted vs. HFD fasted, *P* = 0.0070. Exact adjusted *P* values from Dunn’s multiple comparisons test: CD fed vs. HFD fed, *P* = 0.0052; CD fed vs. HFD fasted, *P* = 0.0325 (**D**); CD fed vs. HFD fasted, *P* = 0.0445 (**E**). Exact adjusted *P* values from Tukey’s multiple comparisons test: CD fed vs. CD fasted, *P* = 0.0483; CD fed vs. HFD fasted, *P* = 0.0245 (**F**); MAM-CM, CD fasted vs. HFD fed, *P* = 0.0007 (**G**). Exact adjusted *P* values from Šídák’s multiple comparisons test: MAM <50 nm LD, CD fed vs. HFD fed, *P* = 0.0413 (**I**). All other significant comparisons, *P* < 0.001 (**D**, **G**, **I**). *p < 0.05, **p < 0.01, ***p < 0.001, ****p < 0.0001. Source data are available online for this figure.

Seipin deficiency is a severe and primary state of adipocyte dysfunction. We next ask whether MCS abnormalities are also a hallmark of the most common form of obesity-related adipocyte dysfunction in mice. Obesity was induced by high-fat diet (HFD) feeding for 3 months, resulting in significant weight gain and systemic insulin resistance (Appendix Fig. S6A,B). Histological analysis confirmed adipocyte hypertrophy in the gonadal white AT (gWAT) of obese mice compared to control lean mice (Appendix Fig. S6C). Using PLA, we found that in lean mice, feeding decreased MAMs and increased contacts involving LDs, including ER–LD and Mi–LD contacts. In contrast, these adaptive changes were absent in obese mice: fasting did not increase MAMs, and the postprandial rise in ER–LD and Mi–LD contacts was also missing (Fig. 5A–C; Appendix Fig. S6E).

TEM analysis showed that fasting increased both the surface area and number of MAMs in control mice, whereas this metabolic adaptation was lost in obese mice (Fig. 5D,H). ER–LD contact number was increased by feeding in lean mice, but not in obese mice, in which ER–LD contact surface was markedly decreased (Fig. 5F,J). Similarly, mitochondrial–LD (Mi–LD) contact number and surface were reduced in obese mice (Fig. 5E,I). Feeding exerted opposite effects on the number of the two MAM subtypes in lean mice: MAM–LDs increased, while MAM–CMs decreased (Fig. 5G). Interestingly, the surface area of both MAM subtypes increased with fasting, indicating complex remodeling in response to nutritional status (Fig. 5K). In obese mice, the fasting-induced increase in MAM–CMs was abolished, and MAM–LD numbers did not rise after feeding, indicating a disruption in the remodeling of tripartite contact sites (Fig. 5G,K; Appendix Fig. S6D).

Altogether, these findings demonstrate that systemic insulin resistance and obesity are associated with a marked impairment in MCS remodeling in gonadal AT, highlighting a loss of metabolic flexibility at the level of organelle interactions.

### MCS disruption is a causal and reversible trigger of adipocyte dysfunction

**Figure 6:**
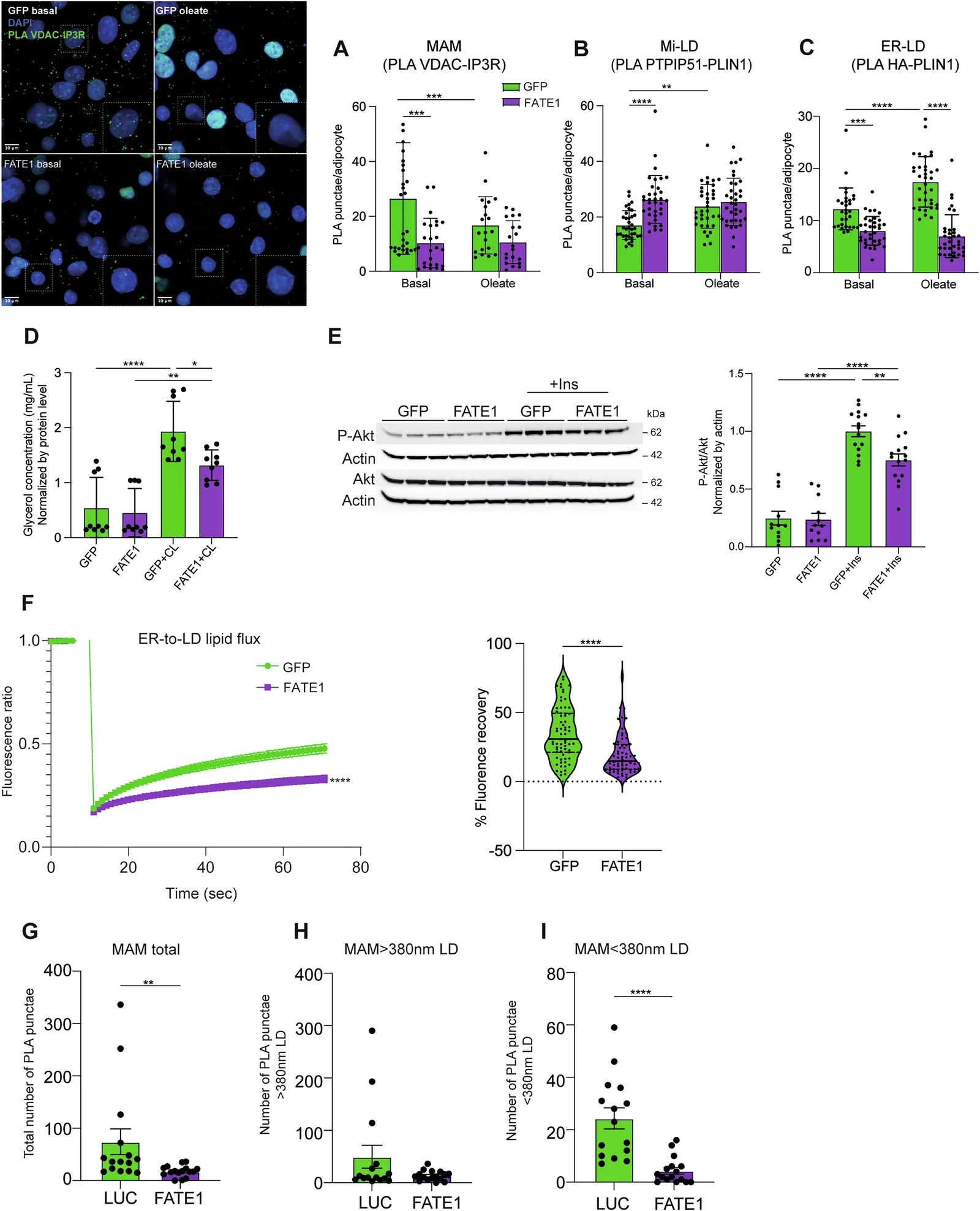
MAM disruption impairs metabolic flexibility and lipid storage in adipocytes. (**A**–**C**) 3T3-L1 adipocytes differentiated for 7 days were infected with adenoviruses expressing GFP or FATE1 and incubated for 6 h in low-glucose medium with or without OA. PLA assays were performed to quantify MAMs (VDAC/IP3R; **A**), Mi–LD contacts (PTPIP51/PLIN1; **B**), and ER–LD contacts (Seipin-HA/PLIN1; **C**). (**D**) Lipolysis was assessed by measuring glycerol release into the extracellular medium following 2 h incubation in the presence or absence of CL316-243. (**E**) Representative immunoblots of phospho-Akt, total Akt, and actin are shown, with corresponding quantification. (**F**) FRAP analysis was performed to assess fluorescence recovery kinetics and lipid droplet fluorescence recovery. (**G**–**I**) MAM abundance was quantified using PLA for VDAC/IP3R combined with PLIN1 immunofluorescence and structured illumination microscopy (SIM). Total PLA signals were quantified (**G**) and classified according to their proximity to lipid droplets as MAM–CM (>380 nm from LDs; **H**) or MAM–LD (<380 nm from LDs; **I**). Data information: (**A**–**C**) Bars represent mean ± SD; 2 or 3 independent biological experiments, with 15 fields acquired per condition per experiment and approximately 50 cells analyzed per field (~1500 cells per condition). (**D**) Bars represent mean ± SD; triplicate measurements from three independent experiments. (**E**) Bars represent mean ± SEM; triplicate measurements from four independent experiments. (**F**) Violin plots show fluorescence recovery (%), with each dot representing one cell; median and quartiles are indicated; *n* > 80 cells per condition from three independent experiments. (**G**–**I**) Bars represent mean ± SEM; *n* = 15 cells per condition from four independent experiments. Statistical analysis: two-way ANOVA (**A**–**D**); one-way ANOVA (**E**); Mann–Whitney test (**F**–**I**). Exact adjusted *P* values from Tukey’s multiple comparisons test: Basal + GFP vs. Oleate + GFP, *P* = 0.0018 (**B**). Exact adjusted *P* values from Tukey’s multiple comparisons test: GFP vs. FATE + CL, *P* = 0.0069; FATE1 vs. FATE + CL, *P* = 0.0024; GFP + CL vs. FATE + CL, *P* = 0.0424 (**D**); GFP + Ins vs. FATE1 + Ins, *P* = 0.0050 (**E**). Exact *P* value from a two-tailed Mann–Whitney test: *P* = 0.0014 (**G**). All other significant comparisons, *P* < 0.0001.*p < 0.05, **p < 0.01, ***p < 0.001, ****p < 0.0001. Source data are available online for this figure.

As in vivo, MCS abnormalities are associated with AT dysfunction and systemic metabolic inflexibility, we used FATE1 ectopic expression in adipocytes to assess the consequences of MAMs disruption. FATE1 is normally expressed in the testis but not in adipocytes, where it acts as a spacer between the ER and mitochondria, thereby decreasing MAM. We overexpressed FATE1 in adipocytes and assessed MAM and other MCS using PLA. FATE1 expression significantly reduced both MAM and ER–LD contact sites under basal and oleate-treated conditions, while Mi–LD increased in the basal state only (Fig. 6A–C; Appendix Fig. S7A). Despite its strong effect in decreasing MAM contacts, FATE1 expression did not modify general mitochondrial or ER parameters, nor did it alter LD properties (Appendix Fig. S8). To assess whether MAM disruption impairs metabolic flexibility, we measured glycerol release following lipolytic stimulation by the β-adrenergic agonist CL316,243. In control cells, glycerol release was robustly induced, while this response was significantly blunted in FATE1-expressing cells (Fig. 6D). Furthermore, FATE1 expression decreased insulin-stimulated Akt phosphorylation by 30%, indicating reduced insulin sensitivity (Fig. 6E; Appendix Fig. S7B). Given the significant alteration in MCS remodeling during lipid loading, we measured ER-to-LD lipid flux using FRAP. FATE1 overexpression decreased fluorescence recovery, highlighting lipid storage impairment (Fig. 6F). In order to better characterize MCS organization in this model, we quantified MAM, MAM-LD, and MAM-CM using PLA MAM (VDAC-IP3R), combined with IF for PLIN1 imaging by SIM. Total MAMs were strongly decreased in FATE1 overexpressing-adipocytes, and this decrease is mainly explained by the decrease of MAMs-LD, as MAMs-CM are not significantly affected (Fig. 6G–I).

**Figure EV5:**
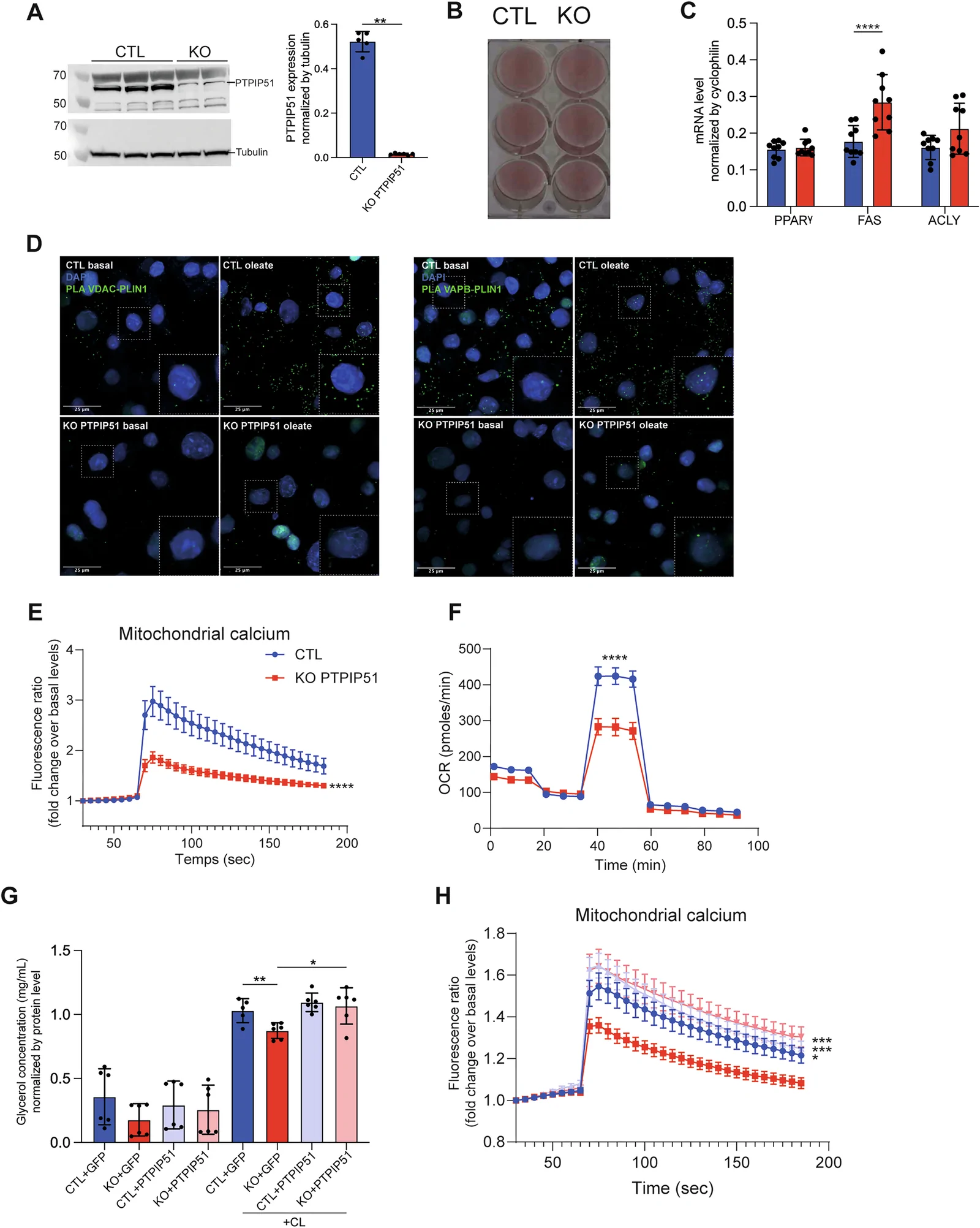
PTPIP51 depletion alters adipocyte differentiation and mitochondrial function. (**A**) Representative immunoblots of PTPIP51 and tubulin in control and PTPIP51 KO 3T3-L1 adipocytes, with corresponding quantification. (**B**) Oil Red O staining of control and PTPIP51 KO 3T3-L1 adipocytes. (**C**) *PPARγ*, *FAS*, and *ACLY* mRNA expression levels were quantified by RT-qPCR in control and PTPIP51 KO adipocytes. (**D**) Representative PLA images of Mi–LD contacts (VDAC/PLIN1) and ER–LD contacts (VAPB/PLIN1) under basal or oleate-loaded conditions in control and PTPIP51 KO adipocytes. (**E**) Mitochondrial calcium uptake was monitored using caged IP₃ photorelease and Rhod-2 fluorescence. (**F**) Oxygen consumption rate (OCR) was measured using Seahorse analysis in control and PTPIP51 KO adipocytes. (**G**) Lipolysis was assessed by measuring glycerol release into the extracellular medium following 2 h incubation in the presence or absence of CL316-243. (**H**) Mitochondrial calcium uptake was monitored as described above. Data information: (**A**) Bars represent mean ± SD; triplicate measurements from two independent experiments. (**C**) Bars represent mean ± SD; triplicate measurements from three independent experiments. (**E**, **F**,** H**) Bars represent mean ± SEM; *n* = 3 independent experiments. (**G**) Bars represent mean ± SD; *n* = 2 independent experiments. Statistical analysis: Mann–Whitney test (**A**); one-way ANOVA (**C**); two-way ANOVA (**E**, **G**, **H**). Exact *P* value from a two-tailed Mann–Whitney test: *P* = 0.0043 (**A**). Exact adjusted *P* values from Dunn’s multiple comparisons test (**G**): KO vs. CTL + CL, *P* = 0.0228; KO vs. CTL + PTPIP51 + CL, *P* = 0.0018; KO vs. KO + PTPIP51 + CL, *P* = 0.0018; CTL + PTPIP51 vs. CTL + PTPIP51 + CL, *P* = 0.0308; CTL + PTPIP51 vs. KO + PTPIP51 + CL, *P* = 0.0308; KO + PTPIP51 vs. CTL + PTPIP51 + CL, *P* = 0.0105; KO + PTPIP51 vs. KO + PTPIP51 + CL, *P* = 0.0105. Two-way repeated-measures ANOVA: time × group interaction, *P* = 0.0018; group effect, *P* = 0.0025 (**H**). *p < 0.05, **p < 0.01, ***p < 0.001, ****p < 0.001.

**Figure 7:**
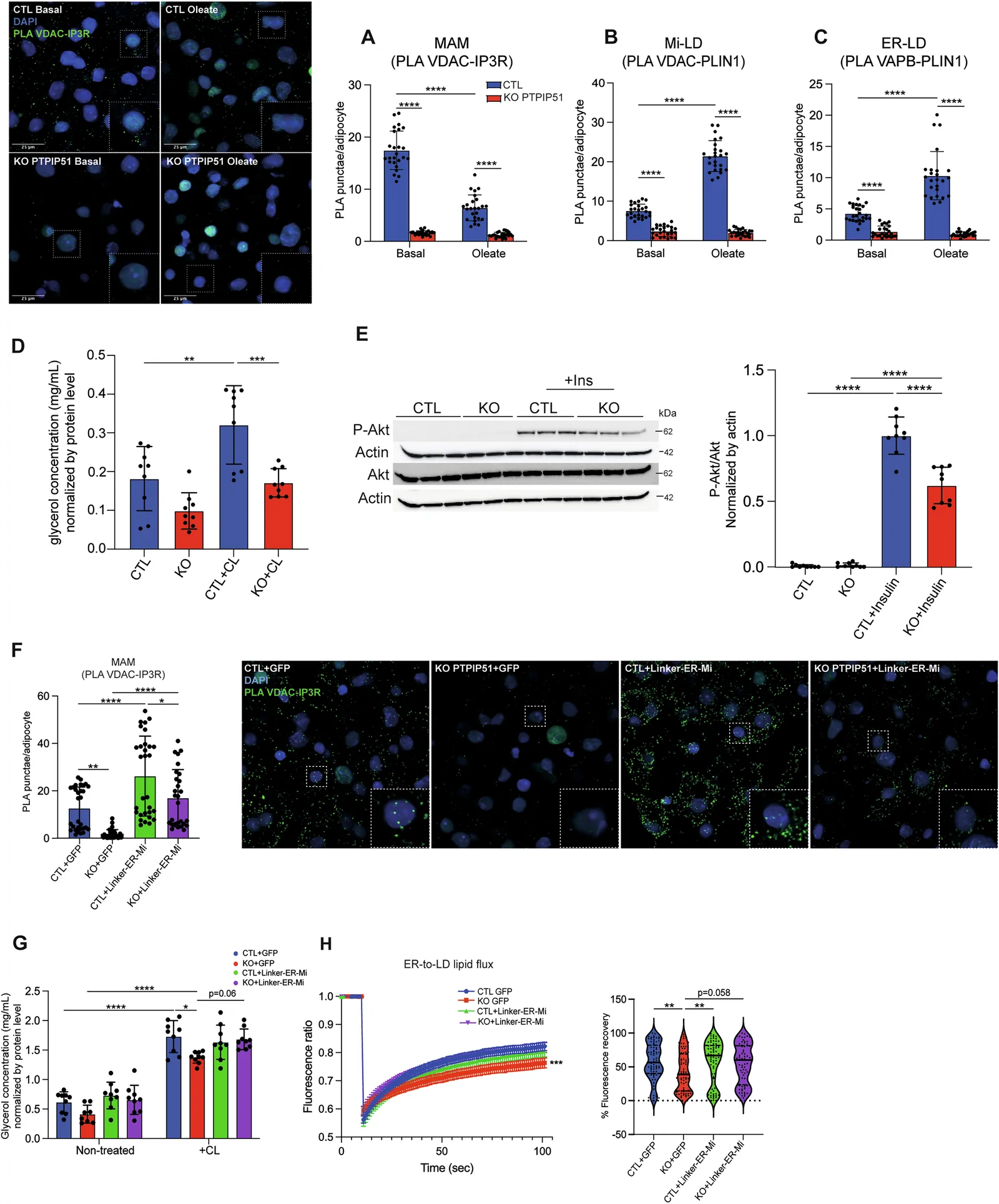
MAM reinforcement is sufficient to restore adipocyte properties. (**A**–**C**) Control and PTPIP51 knockout (KO) 3T3-L1 adipocytes were incubated for 6 h in low-glucose medium with or without oleic acid. PLA assays were performed to quantify ER–mitochondria contacts (VDAC/IP3R; **A**), Mi–LD contacts (VDAC/PLIN1; **B**), and ER–LD contacts (VAPB/PLIN1; **C**). (**D**) Lipolysis was assessed by measuring glycerol release into the extracellular medium following 2 h incubation in the presence or absence of CL316-243. (**E**) Representative immunoblots of phospho-Akt, total Akt, and actin are shown, with corresponding quantification. (**F**,** G**) Control and PTPIP51 KO 3T3-L1 adipocytes were infected with adenoviruses expressing GFP or Linker-ER-Mi. ER–mitochondria contacts were quantified by VDAC/IP3R PLA (**F**), and lipolysis was assessed by glycerol release following CL316-243 stimulation (**G**). (**H**) FRAP analysis was performed to assess fluorescence recovery kinetics and lipid droplet fluorescence recovery. Data information: (**A**–**C**, **F**) Bars represent mean ± SD; three independent biological experiments, with 15 fields acquired per condition per experiment and ~50 cells analyzed per field (~1500 cells per condition). (**E**) Bars represent mean ± SEM; triplicate measurements from three independent experiments. (**G**) Bars represent mean ± SD; triplicate measurements from three independent experiments. (**H**) Violin plots show fluorescence recovery (%), with each dot representing one cell; median and quartiles are indicated; *n* > 80 cells per condition from three independent experiments. Statistical analysis: two-way ANOVA (**A**–**D**, **G**); one-way ANOVA (**E**, **H**). Exact adjusted *P* values from Tukey’s multiple comparisons test: CTL vs. CTL + CL, *P* = 0.0014 (**D**); CTL vs. KO, *P* = 0.0016; CTL + LINKER vs. KO + LINKER, *P* = 0.0118 (**F**); CTL + CL vs. KO + CL, *P* = 0.0177 (**G**). Exact adjusted *P* values from Tukey’s multiple comparisons test: CTL vs. KO PTPIP51, *P* = 0.0092; KO PTPIP51 vs. CTL + LINKER5, *P* = 0.0335 (H). All other significant comparisons, *P* < 0.001.*p < 0.05, **p < 0.01, ***p < 0.001, ****p < 0.0001. Source data are available online for this figure.

To gain more specific insights into the role of MAM-LD in adipocyte physiology, we disrupted MCS organization by deleting PTPIP51, a mitochondrial tether protein localized to both MAMs and Mi–LD contact sites. Using CRISPR/Cas9, we generated PTPIP51 knockout (KO) 3T3-L1 cells. Knockout efficiency was validated by Western blot (Fig. EV5A), and both populations retained the ability to differentiate into adipocytes, as shown by Oil-Red-O staining, and expression of genes implicated in the adipocyte differentiation process (Fig. EV5B,C). PLA analysis revealed that oleic acid failed to induce the expected MCS remodeling in PTPIP51 KO cells (Figs. 7A–C; EV5D). PTPIP51 KO cells display a modest increase in LD area under OA loading, accompanied by a higher frequency of large lipid droplets (Appendix Fig. S9). In addition, OA induced a modest increase in mitochondrial and ER volume and area in both control and KO cells. As previously demonstrated in neurons (Gomez-Suaga et al, 2019), calcium signaling and oxygen consumption were altered in KO cells (Fig. EV5E,F). Lipolysis assays revealed a ~40% decrease in both basal and CL316,243-induced glycerol release in PTPIP51 KO cells (Fig. 7D). Insulin-induced Akt phosphorylation was also reduced by ~50% in KO cells compared to controls (Fig. 7E; Appendix Fig. S7). To ensure the specificity of our model, we overexpressed PTPIP51 in KO PTPIP51 cells and observed a rescue of both calcium flux and the lipolysis defect (Fig. EV5G,H).

Finally, to determine whether the functional defects observed in PTPIP51-deficient adipocytes were specifically due to impaired MAM-LD organization, we expressed Linker-ER-Mi that strongly increased MAMs contact, as demonstrated by PLA (Fig. 7F). To assess the functional relevance of MAM reinforcement, we performed lipolysis assays. In PTPIP51 KO cells, lipolytic capacity was impaired, but was fully rescued upon Linker-ER-Mi expression (Fig. 7G). Using FRAP analysis, we confirmed that ER-to-LD lipid transfer was reduced in PTPIP51 KO adipocytes. Strikingly, expression of Linker-ER-Mi fully restored fluorescence recovery kinetics, indicating a rescue of ER-to-LD lipid flux (Fig. 7H). This suggests that strengthening ER–mitochondria contacts, thus restoring MAM-LD architecture, is sufficient to restore key adipocyte functions disrupted by *PTPIP51* loss.

## Discussion

**Figure 8:**
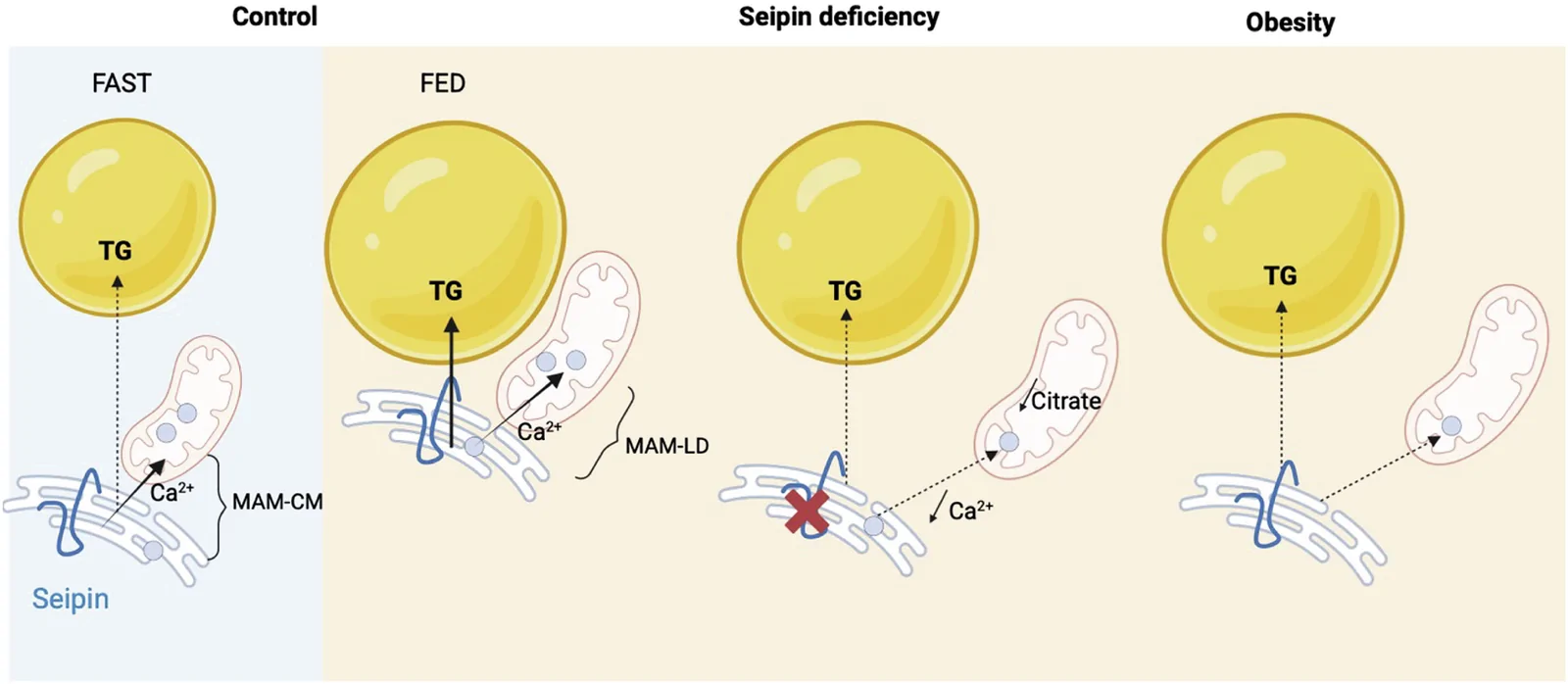
Tripartite ER–mitochondria–lipid droplet contact sites regulate adipocyte metabolic flexibility. Under physiological conditions, Seipin localizes at membrane contact sites between the endoplasmic reticulum (ER), mitochondria, and lipid droplets (LDs), thereby coordinating organelle communication in response to nutritional cues. During fasting, Seipin-enriched mitochondria-associated membranes preferentially support ER–mitochondria contacts (MAM–CM), promoting mitochondrial Ca²⁺ transfer and balanced triglyceride mobilization. In the fed state, Seipin facilitates interactions between MAMs and lipid droplets (MAM–LD), coupling Ca²⁺ signaling with triglyceride storage in expanding lipid droplets. Seipin deficiency disrupts ER–mitochondria–LD contact organization, alters Ca²⁺ flux and mitochondrial citrate metabolism, and impairs lipid storage. More broadly, altered membrane contact-site dynamics may contribute to the metabolic inflexibility associated with adipose tissue dysfunction in obesity.

Understanding how metabolic flexibility is regulated in adipocytes is crucial to pave the way for new therapeutic strategies to tackle the metabolic complications associated with obesity. Here, starting with the study of an extreme and rare case of primary adipocyte dysfunction, the CGL due to Seipin deficiency, we studied the role of MCS involving the ER, the mitochondria, and the LD in adipocyte homeostasis. Indeed, we highlight that the MAM-LD tripartite contact sites are promoted by lipid loading and that they regulate lipid flux from the ER to the LD in a calcium-dependent manner. Then, we established the landscape of MCS remodeling under nutritional stimulation and demonstrated that those adaptations are lost in the AT of obese mice. We further demonstrate that specific MCS disturbances are sufficient to induce lipolysis and insulin signaling alteration in adipocytes. Interestingly, the abnormalities associated with MCS disruption were corrected by the use of a synthetic linker, the Linker-ER-Mi, that reinforces MAMs. Altogether, we highlight the key role of MAM-LD in lipid handling and the importance of MCS involving the ER, the LD and the mitochondria, in metabolic flexibility. A working model summarizing our findings is presented in Fig. 8.

Seipin is a critical regulator of adipocyte homeostasis, since its deficiency leading to severe adipocyte dysfunction and lipoatrophy in both humans and mice. Seipin is known to be enriched at endoplasmic reticulum–lipid droplet (ER–LD) contact sites, and in fibroblasts derived from patients with Seipin deficiency, these contacts appear disorganized (Salo et al, 2016). Additionally, Seipin is enriched at MAMs, and its deficiency disrupts calcium homeostasis (Combot et al, 2022; Ding et al, 2018; Guyard et al, 2022). In this study, we demonstrate that Seipin deficiency reduces all three contact types involving the ER, mitochondria, and LD, with a particularly pronounced effect on MAM-LD contacts. Interestingly, under OA loading in vitro, Seipin deficiency reduces MAM surface area while increasing MAM number, suggesting a redistribution toward smaller but more numerous contacts. Lipidomic analysis of LDs isolated from iATSKO mouse AT revealed decreased TG and increased diacylglycerol DG content, suggesting a defect in TG synthesis or transport. Using FRAP analysis, we showed that Seipin deficiency impairs ER-to-LD lipid transfer in vitro, and that reinforcement of MAM-LD contacts can restore this lipid transfer. Although we lack specific tools to selectively reinforce MAM-LD contacts, we employed Linker-ER-Mi, which increases proximity between the ER and mitochondria. Using both SIM and TEM, we confirmed that Linker-ER-Mi primarily reinforces MAM-LD contacts in Seipin-deficient adipocytes. To further elucidate the mechanism of action, we knocked down MCU to prevent calcium entry into mitochondria. This intervention abolished the effect of Linker-ER-Mi, demonstrating that the restoration of lipid flux is calcium-dependent. Thus, beyond restoring the structural integrity of MAM-LD, recovering the calcium exchange is essential. Supporting this, studies in hepatocytes from yellow catfish have shown that phosphatidic acid loading promotes lipid deposition by recruiting Seipin to MAMs (Chi et al, 2024). In their model, Seipin deficiency did not alter MAM structure or number, but did prevent lipid accumulation in a calcium-dependent manner, further highlighting the crucial role of calcium signaling modulation in the effect of MAMs on lipid storage. The precise mechanisms by which calcium regulates lipid storage remain to be elucidated. It is plausible that calcium controls ATP production required for lipid transfer protein function, that some of these proteins are directly calcium-dependent, or that calcium regulates citrate production, the initial substrate for lipogenesis. Notably, we observed that citrate production is decreased in Seipin-deficient adipocytes and restored by Linker-ER-Mi expression. In addition, when DGAT inhibitors were used, lipid loading during FRAP was abolished, supporting that the observed phenomenon relies on de novo TG synthesis.

How can we integrate our findings with previous research on Seipin function? A substantial body of work demonstrates that Seipin forms oligomeric, donut-like complexes whose central hydrophobic helices bind and concentrate triacylglycerols (TAG), thereby facilitating LD formation (Prasanna et al, 2021; Sui et al, 2018; Yan et al, 2018; Zoni et al, 2021). Loss of Seipin disrupts ER membrane curvature, and Seipin preferentially localizes to tubular ER subdomains rather than sheet-like regions (Santinho et al, 2020). Since the reinforcement of MAM-LD contacts is sufficient to restore lipid flux even in the absence of Seipin, we propose that Seipin plays a central role in organizing MAM-LD contacts during lipid loading, thereby enabling efficient TG synthesis and storage in the LD. Notably, a recent study that thoroughly analyzed the protein composition of MAM-LD contacts demonstrated that these sites are enriched in enzymes involved in TG synthesis, such as GPAT, AGPAT2, LIPIN, and DGAT2 (Najt et al, 2023). Strikingly, Seipin has been shown to interact with GPAT3 (Pagac et al, 2016), LIPIN (Sim et al, 2012) and AGPAT2 (Talukder et al, 2015). However, our proteomic analysis did not reveal differential enrichment of these proteins in the LD environment of Seipin-deficient AT compared to controls. Therefore, we do not propose that Seipin directly controls their recruitment. Instead, we suggest that Seipin plays a central role in scaffolding the MAM-LD contacts, determining their structural properties to ensure their functional role in LD lipid storage. MAM-LD contacts have only recently been named and characterized (Guyard et al, 2022; Najt et al, 2023), and we discuss our findings in the context of emerging literature to better understand their role in lipid handling. Using TEM and PLA in both AT and 3T3-L1 adipocytes, we show that lipid loading increases contacts involving the LD, specifically ER/LD and mitochondria/LD (Mi/LD) contacts. However, lipid loading exerts opposite effects on MAM subtypes: oleic acid increases MAM-LD contacts while decreasing MAM-CM contacts. Consistently, in the liver, oleic acid has been reported to decrease MAM-CM and increase MAM-LD contacts (Najt et al, 2023). Therefore, we are proposing that the MAMs involving CM would be the “classical” MAMs, known to support catabolic processes during fasting (Theurey and Rieusset, 2017), while those involving PDM are required during lipid loading challenge. Beyond Seipin deficiency, we demonstrate that disruption of MAM-LD contacts by FATE1 overexpression, as well as global MCS defects in PTPIP51 knockout adipocytes, severely impairs lipid transfer from the ER to the LD. As observed in Seipin-deficient adipocytes, Linker-ER-Mi rescues this defect in PTPIP51-deficient cells, supporting the hypothesis that the effect of PTPIP51 KO relies indeed on its action in structuring MCS (Gomez-Suaga et al, 2017). In HeLa cells, the lipid transfer proteins ORP5 and ORP8 orchestrate LD biogenesis at MAM-LD sites by interacting with Seipin and regulating its localization (Guyard et al, 2022). In adipocytes, MAM-LD contacts have not yet been directly explored. One study reported that the MAM-localized mitochondrial fission protein DRP1 is involved in lipid trafficking to LDs, suggesting a need for cooperation among all three organelles for LD biogenesis, though without directly demonstrating the involvement of MAM-LD (Li et al, 2020). Additionally, the outer mitochondrial membrane protein MIGA2 reinforces mitochondria/LD contacts and promotes lipogenesis (Freyre et al, 2019). We attempted to isolate MAM-LD structures, but our yields were insufficient for unbiased analysis, particularly in comparison to MAM-CM. Further studies are needed to precisely identify the key players and mechanisms that regulate lipid metabolism at MAM-LD contacts. Another limitation of our study is that we could not directly assess tripartite contact-site dynamics by live imaging, as the optical properties of differentiated adipocytes, particularly their high LD content, prevented robust simultaneous visualization of the ER, mitochondria, and LD. Substantial methodological development and optimization should be performed in the future to access in real-time the interactions between the three organelles.

We evaluated the overall contribution of MCS to adipocyte metabolic flexibility. Glucose loading decreased MAM abundance, consistent with previous findings in the liver and supporting the idea that MAM formation are induced by fasting (Theurey and Rieusset, 2017). We found that MAM regulation by glucose depends on AMPK and PKA, two key signaling pathways involved in adaptation to fasting. Similar findings were reported in the liver previously (Theurey et al, 2016). Lipolysis, which is stimulated by fasting and the PKA pathway (Morigny et al, 2016), was impaired by specific MAM depletion but restored by Linker-ER-Mi–mediated MAM reinforcement. Furthermore, disruption of MAMs induced cell-autonomous insulin resistance in adipocytes, reinforcing their role as metabolic hubs essential for regulating metabolic flexibility.

Finally, having shown that MAM-LD alterations are associated with severe adipocyte dysfunction and that MCS remodeling is a key player in metabolic flexibility, we investigated whether MCS involving the ER, LD, and mitochondria is altered in the AT of obese mice—a model characterized by metabolic inflexibility. We demonstrated that nutrient-induced MCS remodeling is abolished in the AT of obese mice at a stage when they develop insulin resistance. Most precisely, while MAM-CM were increased in the AT of obese mice, MAM-LD were nearly absent. This urges us to better define the type of MAM we are referring to when describing the MCS. The state of MAM in the livers of obese mice has been the subject of considerable debate. While some groups found them decreased (Tubbs et al, 2014) other found them increased (Arruda et al, 2014; Parlakgul et al, 2024). It would be interesting to further clarify the type of MAM discussed in these two reports. The combination of PLA and SIM analysis used here, based on a previous publication (Monteiro-Cardoso et al, 2023), could be a simple tool for determining the MAM-LD state. However, an important limitation of our study that should be addressed in future work is that, as mentioned above, PLA was used to quantify contact sites and, whenever possible, validated by TEM. Yet, we did not investigate the molecular diversity of these contacts, particularly their protein composition. This is important because distinct contact subtypes can support different metabolic functions. For example, in muscle, Perilipin 5–mediated Mi–LD contacts promote fatty acid catabolism (Kimmel and Sztalryd, 2014), whereas in adipocytes, Miga2-mediated Mi–LD contacts favor lipogenesis (Freyre et al, 2019). Therefore, defining the protein composition of contacts across cellular contexts and nutritional states could help establish a functional cartography of contact subtypes with distinct biological roles. Nevertheless, dysfunctional adipocytes in the context of obesity exhibit profound alterations in lipid and glucose metabolism, hallmarks of metabolic inflexibility (Morigny et al, 2021). In particular, reduced lipogenesis activity in the AT is a hallmark of insulin resistance and AT failure in the context of obesity (Smith and Kahn, 2016). Based on our findings, we propose that abnormalities in MCS may contribute to this state of metabolic inflexibility, as in vitro, MCS disruption alone induces both insulin resistance and impaired lipolysis.

## Conclusion

Altogether, our work establishes MCS disruption as a hallmark of adipocyte dysfunction in two murine models of AT failure: CGL and diet-induced obesity. Specifically, contacts involving the ER, LDs, and mitochondria are significantly impaired. Using synthetic linkers to reinforce these connections corrected several adipocyte abnormalities, validating MAM-LD as a potential therapeutic target. Given that MCS remodeling is crucial for metabolic flexibility, interventions such as Linker-ER-Mi, which clamp MAMs in a specific state, appear unpromising. Conversely, strategies that restore remodeling capacity or enable transient MAM-LD augmentation during the postprandial phase represent promising future therapeutic avenues. This approach is particularly relevant considering the persistence of AT dysfunction (“fat memory”) even after weight loss. Targeting adipocyte dysfunction directly, alongside next-generation weight-loss medications, offers a synergistic therapeutic strategy.

## Methods

**Table:** Reagents and tools table

Reagent/resource	Reference or source	Identifier or catalog number
**Experimental models**		
3T3-L1	ATCC	CL-173
C57BL/6 mice J	Charles River	027
C57BL6J Bscl2lox/lox	European Mouse Mutant Cell Repository	Clone HEPD0757_2_A10
C57BL6J ERT2-Adipoq-CRE	Pr S Offermanns lab (Max Plank institute)	Sassmann et al, (2010)
**Recombinant DNA**		
Adenovirus FATE1	Vectorbuilder	AVM(VB211008-1313mbz)-C
Adenovirus LUC	Vectorbuilder	*AVM(VB211201-1122hmj)-C*
Adenovirus GFP	Vectorbuilder	AVM(VB010000-9299hac)-C
Adenovirus Linker RE-Mito	Vectorbuilder	AVM(VB211012-1315jdk)-C
**Antibodies**		
anti-Actin	Thermofisher	MA1-140
anti-p-Ser473-AKT	Cell signalling	#4060
anti-AKT	Cell signalling	#4691
anti-Calnexin	Proteintech	66903
anti-IP3R1-2-3	Santa Cruz	377518
anti-MCU (DZ3B)	Cell signalling	#14997
anti-p-PDH	Cell signalling	#31866
anti-PDH	Cell signalling	#2784
anti-Perilipin1	Progen	GP-29
anti-Perilipin1	Progen	651156
anti-PDI	Thermo Fisher	MA3-018
anti-PTPIP51 (RMDN3) for IF	Atlas antibody	HPA009975
anti-PTPIP51 (WB)	Proteintech	20641-1-AP
anti-TOM20	Thermo Fisher	MA5-34964
anti-Tubulin	Thermo Fisher	MA1-80017
anti-VAPB	Sigma-Aldrich	HPA013144
anti-VDAC1	Abcam	15895
**Oligonucleotides and other sequence-based reagents**		
On-target-plus siRNA smart pool against mouse Bscl2	Horizon discovery	L-040550-01-0005
On-target-plus siRNA non-targeting pool	Horizon discovery	D-001810-10
crRNA:tracrRNA for CRISPR/Cas9 editing (PTPIP51)	ACCAAGUAACAGUCCCAACC and UAUGACCUGUGCUCUUACCC	
PTPIP51 QPCR primers	Forward: 5’-*GGTTGTCACGTACAGGGTGT*-3’ 5’- *GGCGCGACCAGAACTAAAAG* -3’	
PTPIP51 reverse primer	Reverse: 5’- *GGCGCGACCAGAACTAAAAG* -3’	
Oligonucleotides for Real-Time quantitative PCR - *cytochrome c oxidase II (mt-Co2)*	Forward:CCACTTCATCTTACCATTTATTATCGC Reverse:TTTATCTGCATCTGAGTTTAATCCTGT	
Oligonucleotides for Real-Time quantitative PCR - Genomic *β-actin*	Forward:CTGCCTGACGGCCAGG Reverse:CTATGGCCTCAGGAGTTTTGTC	
**Chemicals, enzymes and other reagents**		
Cas9 protein (Alt-R^TM^ S.p. Cas9 Nuclease V3)	IDT	1081058
Alt-R™ CRISPR-Cas9 crRNA	IDT	
Alt-R™ CRISPR-Cas9 tracrRNA	IDT	1072532
Lipofectamine RNAiMax	Thermofisher	13778150
BODIPY 505/515	Cayman chemical	25893
BODIPY558/568 C12	Thermo Fisher	D3835
Oleic acid	Sigma-Aldrich	0-1383
Duolink™ In Situ FarRed kit	Sigma-Aldrich	#DUO92013
TrypLE Express	Thermo Fisher	12605036
caged(1,4,5)IP3/PM	Sichem	cag-iso-2-145-100
Pluronic acid F-127	Thermo Fisher	P6867
Rhod-2, AM	Thermo Fisher	R1244
^13^C_6_-glucose	Eurisotop	CLM-1396-1
Seahorse XFe96/XF pro Flux pack	Agilent	103792-100
Amaxa Cell Line Nucleofactor Kit V	Lonza	VCA-1003
**Software**		
Cellpose model	N/A	(Pachitariu and Stringer, 2022)
CellStitch	N/A	(Liu et al, 2023)
Imaris	Oxford instrument	V10
Amira	Thermo Fisher	V2024.2
GraphPad Prism	Dotmatics	V10
NIS elements	Nikon	
Metamorph	Molecular devices	
ChemiDoc	Bio-Rad	
Fiji (ImageJ)	GNU General Public License.	(Schneider et al, 2012)
EasyFRAP	N/A	(Rapsomaniki et al, 2012)
Proteome Discoverer	Thermo Fisher	

### 3T3-L1 cell culture and differentiation

Mouse embryonic 3T3-L1 cells (ATCC CL-173) were maintained in medium A (DMEM with 4.5 g/L glucose, 10% calf serum, 1% penicillin/streptomycin, and 1% glutamine). Cells were seeded at a density of 20,000 cells/mL in medium B (DMEM with 4.5 g/L glucose, 10% fetal calf serum (FCS), 1% penicillin/streptomycin, and 1% glutamine). Two days post-confluence, adipogenic differentiation was induced using medium B supplemented with 2 µM insulin, 500 µM IBMX, and 1 µM dexamethasone (differentiation medium, DMI). On day 3, cells were switched to medium B containing only 2 µM insulin. From day 6 onward, cells were maintained in medium B alone. Cells are tested for mycoplasma twice a year.

### Animals

*Bscl2*^lox/lox^ was established at the Institut Clinique de la Souris (http://www.phenomin.fr/fr-fr/). Three clones were ordered from the European Mouse Mutant Cell Repository (https://www.eummcr.org/). All three clones were validated by Southern blot (internal Neo probe) and PCR confirmation of the presence of the 3’ LoxP site. Clone HEPD0757_2_A10 was karyotyped by chromosome spreading and Giemsa staining and microinjected in BALB/cN blastocysts. Resulting male chimeras were obtained, and germ line transmission was achieved for the tm1a allele (knock-out first with conditional potential). The KO allele (tm1b) was obtained after breeding tm1a animals with a Cre deleter line (Birling et al, 2012) and phenotyped by PHENOMIN-ICS. All data are freely available through the IMPC website (https://www.mousephenotype.org/data/genes/MGI:1298392). The cKO Bscl2 allele (tm1c; Lox/Lox) was obtained by breeding the tm1a allele with a Flp deleter line (Birling et al, 2012). *Bscl2*^lox/lox^ mice were crossed with ERT2-Adipoq-CRE (Sassmann et al, 2010) mice to generate Bscl2^lox/lox^ mice X ERT2-Adipoq-CRE mice; i.e., iATSKO mice. For obesity studies, male C57BL/6 J mice were purchased from Charles River and fed a high-fat diet (HFD) (60% of calories from fat, 20% of calories from protein, and 20% calories from carbohydrate, #D12492 Safe Diets), or a normal chow diet ad libitum for a period of 12 weeks. All mice had ad libitum access to food and water and were housed in the same open mouse facility on a 12/12-h light/dark day/night cycle. Animal care and study protocols were approved by the French Ministry of Education and Research and the ethics committee N°6 in animal experimentation (APAFIS #43225-2023042614548433 v5 and APAFIS #37839-2022063008451968 v8) and were in accordance with the EU Directive 2010/63/EU for animal experiments. Before initiating any animal protocol, animals were randomized to minimize bias; however, experiments were not performed in a blinded manner.

### Insulin tolerance test (ITT)

Food was removed 2 h before the initiation of the tolerance test according to the recently published guidelines (Moro and Magnan, 2025). For ITT, mice were injected i.p. with 1 IU/kg body weight insulin at time 0. Blood glucose levels were monitored using glucometer strips at 0, 15, 30, 60, and 120 min on 2.5 µL samples collected from the tail.

### Generation of PTPIP51 knockout cells in the 3T3-L1 line

3T3-L1 preadipocytes were cultured in medium A and passaged upon reaching ~50% confluence. Cells were trypsinized, collected, and counted. A total of 5 × 10^5^ cells were electroporated using the Amaxa Cell Line Nucleofector Kit V (VCA-1003, Lonza) with a ribonucleoprotein complex consisting of recombinant Cas9 protein (Alt-R™ S.p. Cas9 Nuclease V3, 21 µM final, IDT), and two pre-assembled crRNA:tracrRNA duplexes (crRNA sequences ACCAAGUAACAGUCCCAACC and UAUGACCUGUGCUCUUACCC, IDT), targeting the *murine Rmdn3* gene (encoding PTPIP51, exon1). As a control, cells were electroporated with Cas9 and tracrRNA only, without crRNA. Forty-eight hours post-electroporation, cells were trypsinized and sorted using flow cytometry (FACS Aria III/Influx), with single cells seeded into individual wells of a 96-well plate to generate clonal populations. Clones were screened for successful biallelic gene disruption using genomic PCR (PTPIP51-F: *GGTTGTCACGTACAGGGTGT;* PTPIP51-R: *GGCGCGACCAGAACTAAAAG*). Selected clones were expanded and evaluated for their adipogenic differentiation potential. Respectively, four and five clones showing comparable differentiation capacities were pooled to generate two distinct populations referred to as “Control” and “PTPIP51 KO” throughout this study. These two populations are available on request.

### siRNA transfection and reverse adenoviral infection

On day 6 of differentiation, cells were harvested from T75 flasks using TrypLE Express (Thermo Fisher), and resuspended in 12 mL of medium B per flask. For transfection, a mix containing 20 nM siRNA, 2.5 µL Lipofectamine RNAiMAX (Thermo Fisher), and 47.5 µL Opti-MEM (Thermo Fisher) was prepared and added to the bottom of collagen IV-coated Ibidi 8-well chambers. Then, 0.25 mL of the cell suspension was added to each well. When required, adenoviruses were added directly to the transfection mix at the indicated MOI (see Fig legends). Cells were harvested and analyzed 72 h post-transfection. The siRNA pool targeting BSCL2 was obtained from Horizon Discovery.

### Confocal analysis of organelle area

Differentiated adipocytes were washed twice with PBS, fixed in 4% paraformaldehyde (PFA) for 10 min, and permeabilized with 0.5% saponin for 20 min. Cells were then blocked for 1 h at room temperature in PBS containing 3% BSA and 0.05% Tween. Primary antibodies were incubated for 1 h at room temperature in PBS supplemented with 1% BSA, followed by three washes in PBS. The following primary antibodies were used: mouse anti-Calnexin (1:100, Proteintech, 66903) and rabbit anti-TOM20 (1:100, Thermo Fisher, MA5-34964). Secondary antibodies were incubated for 30 min under the same conditions. In parallel experiments, cells were stained after fixation with 0.1 µM BODIPY 505/515 (Cayman Chemical) for 15 min and washed twice with PBS. Nuclei were counterstained with DAPI. Slides were mounted using ProLong mounting medium. Z-stack images were acquired using a Nikon A1RSi confocal microscope equipped with a 60× oil-immersion objective. Images were imported into IMARIS software, where surface segmentation was performed to quantify the volume and area of each organelle category.

### Proximity ligation assay (PLA)

PLA was performed on 3T3-L1 cells using the Duolink™ In Situ FarRed kit (#DUO92013, Sigma-Aldrich) following the manufacturer’s instructions. After PBS washes, cells were fixed in 4% PFA for 10 min and washed again in PBS. Permeabilization was performed using 0.2% saponin in PBS. Blocking was done at room temperature for 60 min using a solution of 3% BSA, 0.1% saponin, and 20 mM glycine in PBS. Primary antibodies were incubated overnight at 4 °C in PBS containing 2% BSA, 0.1% saponin, and 20 mM glycine. Antibodies used: mouse anti-IP3R1-2-3 (1:100, Santa Cruz 377518), mouse anti-Perilipin1 (1:500, Progen 651156), rabbit anti-VDAC1 (1:200, Abcam 15895), rabbit anti-VAPB (1:200, Sigma-Aldrich HPA013144), anti-PTPIP51 (Atlas antibody, HPA009975). Subsequent steps followed the kit protocol. Nuclei were counterstained with DAPI and washed in Tris-HCl (200 mM), NaCl (137 mM), and 0.05% Tween. Z-stack images were acquired on a Nikon A1RSi confocal microscope with a 60x oil immersion objective. PLA focus quantification per cell was performed using ImageJ 2.0 with the following settings: nuclear area 25–100 µm²; PLA dot size 0–1 µm². Statistical analysis was conducted using the Mann–Whitney test (GraphPad Prism).

### Super-resolution structured illumination microscopy (SIM)

Differentiated adipocytes treated with oleate (700 µM) during 6 h were washed twice with PBS, fixed in 4% PFA for 10 min, and permeabilized using 0.5% saponin for 20 min. Blocking was done at room temperature for 60 min using a solution of PBS-Tween BSA 3%. Primary antibodies were incubated for 1 h at room temperature in PBS with 1% BSA, followed by three PBS washes. Antibodies used: mouse anti-PDI (1:50 Thermo MA3-018), guinea pig anti-Perilipin1 (1:50 Progen), rabbit anti-TOM20 (1:100 Thermo MA5-34964). Secondary antibodies were incubated 30 min under the same conditions. Slides were mounted using ProLong mounting medium. SIM imaging was performed using a Nikon A1 N-SIM microscope equipped with a 100×/1.40 NA oil immersion objective. Structured illumination patterns were projected using 488, 561, and 642 nm lasers. Fluorescence was captured using an EMCCD camera (iXon 885, Andor Technology). 3D stacks were reconstructed with NIS software. LD segmentation was performed using a custom workflow based on a 2D retrained Cellpose model for 2D segmentation (Pachitariu and Stringer, 2022), and 3D reconstruction using CellStitch (Liu et al, 2023) (see Data availability section) and imported afterward in IMARIS, where spot segmentation and distance assessment (spot to surface and spot to spot) were then carried out.

### Fluorescence recovery after photobleaching (FRAP)

Differentiated 3T3-L1 adipocytes were incubated for 4 h in medium B supplemented with BODIPY558/568 C12 (2 µM) and oleic acid (OA, 20 µM). After PBS washes, cells were transferred to FluoroBrite medium containing OA (20 µM). Imaging was performed on a Nikon A1RSi confocal microscope using a 20x objective. A region of interest (ROI) located on a lipid droplet (LD) was selected, and fluorescence was recorded for 10 s before photobleaching with a high-intensity laser for 500 ms. Fluorescence recovery was monitored for 90 s. Data were analyzed using ImageJ 2.0 and EasyFRAP (Rapsomaniki et al, 2012). Two-way ANOVA was used for statistical comparisons (GraphPad Prism).

### FIB-SEM sample preparation

Fat tissues from wild-type (WT) and mutant specimens were dissected and washed three times in phosphate-buffered saline (PBS). Samples were then fixed in 2.5% glutaraldehyde (Electron Microscopy Sciences, MA, USA) prepared in 0.2 M cacodylate buffer (pH 7.2) overnight at 4 °C. Following fixation, tissues were washed three times in fresh 0,1 M cacodylate buffer (pH 7.2) and post-fixed in 1% osmium tetroxide (Electron Microscopy Sciences) in 0,1 M cacodylate buffer supplemented with 1.5% potassium ferrocyanide for 1 h in the dark. After three washes in distilled water, samples were incubated for 30 min at room temperature in 0.2% tannic acid (in water), then post-fixed a second time in 1% osmium tetroxide for 1 h and washed again. Samples were dehydrated through a graded ethanol series (25, 50, 70, 90, and 100%) and embedded in epoxy resin (PolyBed 812, Electron Microscopy Sciences), followed by polymerization for 58 h at 60 °C.

### FIB-SEM imaging

For focused ion beam-scanning electron microscopy (FIB-SEM) acquisition, resin-embedded tissue blocks were mounted on aluminum stubs and sputter-coated with a 20 nm gold-palladium layer using a Leica ACE600 coater. Imaging was performed with the specimen stage tilted at 54 °C, maintaining a 5 nm working distance at the coincidence point of the electron and gallium beams. Serial block milling was performed using a 500 pA milling current, removing 16 nm slices from the specimen surface at each step. Images were acquired at 1.5 kV using the in-lens EsB detector, with the EsB grid set between −300 and −500 V. The final dataset had a voxel size of 15 nm in x, y, and z dimensions.

### FIB-SEM image analysis

Images were aligned with the plug-in Stackreg from Fiji: P.Thévenaz, U.E.Ruttimann, M.Unser, “A Pyramid Approach to Subpixel Registration Based on Intensity” IEEE Transactions on Image Processing, vol.7, no.1, pp.27–41, January 1998. The rest of the workflow was done on Amira software version 2024.2. The segmentations were produced with the AI-assisted segmentation module. Seven ROIs (rectangular regions containing the annotations and background provided to the neural network) were manually segmented for the wild-type condition, including mitochondria, adipocyte plasma membrane, nucleus, and lipid droplets, whereas ten ROIs were generated for the CRE condition, including mitochondria, adipocyte plasma membrane, and lipid droplets. In both cases, the annotations were manually done with the brush or the magic wand tools. Then the ResNet18 deep learning model was selected, with 30 epochs chosen for the training. In the end, the prediction was corrected manually either in 2D with the brush, the pick or the lasso tools, or in 3D with the pick or the lasso tools.

### Mitochondrial calcium flux assay

Differentiated adipocytes were washed with HBSS and incubated for 1 h at 37 °C in a loading solution (900 µL HBSS + 100 µL Fluoforte reagent B, ENZO kit) containing 3 µM caged IP3 (Sichem), 5 µM Fluo4 (Thermo Fisher) or 10 µM Rhod-2 (Thermo Fisher), and 0.3% Pluronic F-127 (Thermo Fisher). After loading, cells were washed in HBSS. Time-lapse imaging was performed using a Leica DMI 6000B microscope controlled by Metamorph software. Following baseline acquisition, IP3 photorelease was triggered using a UV flash.

### Oxygen consumption measurements with the seahorse system

Oxygen consumption rate (OCR) was measured with a Seahorse XF96 Extracellular Flux Analyzer (Agilent). Before the analysis, differentiated adipocytes were maintained in their normal culture conditions. 30 min prior to OCR measurements, the cells were transferred to assay medium (XF base medium supplied with L-glutamine, 10 mM glucose and 1% FBS).

### Mitochondrial DNA quantification

Total DNA was isolated from adipose tissue (AT) using the QIAamp DNA Kit (Qiagen) following the manufacturer’s protocol. Mitochondrial DNA content was quantified by qPCR using 50 ng of total DNA, by measuring the mitochondrial-encoded *cytochrome c oxidase II (mt-Co2)* gene relative to the nuclear *β-actin* gene.

### Lipid droplets isolation

LD were isolated from adipose tissue under cold conditions. Briefly, ~500 mg of adipose tissue was collected, weighed, and finely minced with scissors. The minced tissue was transferred directly into a Dounce homogenizer and homogenized in 3 mL of SHE buffer (250 mM sucrose, 5 mM HEPES, 2 mM EDTA, pH 7.2) using ten strokes. The homogenate was filtered through a 100 µm cell strainer into a 50 mL Falcon tube, then distributed into two 2 mL safe-lock tubes. Samples were centrifuged at 4000×*g* for 15 min at 4 °C. After centrifugation, the infranatant was carefully removed with a P200 pipette, while preserving the lipid droplet fraction adhering to the tube wall. The bottom of each tube was washed twice with 200 µL PBS to remove residual cell debris. Lipid droplets from both tubes were then recovered together in a final volume of 200 µL PBS.

### Lipidomics analysis

LD fraction was resuspended in 400 µL deionized water. Lipid extraction was performed by adding 1600 µL of cyclohexane/isopropanol (3:2), followed by 1 h agitation at room temperature and centrifugation at 10,000×*g* for 5 min. The organic upper phase was transferred to glass tubes and evaporated at 60 °C under nitrogen. Dried lipids were resuspended in isopropanol/acetonitrile/water (65:30:5) and analyzed by liquid chromatography coupled with mass spectrometry (LC-MS).

### Proteomic analysis

Bottom-up mass spectrometry (MS)-based proteomics was used to characterize the proteome. This approach enables the identification and quantification of proteins through the analysis of peptide fragments generated by enzymatic proteolysis. Plasma samples (5 µL) and tissue homogenates (40 µL, 1 mg/mL in PBS) were subjected to tryptic digestion prior to untargeted MS-based proteomic analysis, as previously described (Rodrigues Oliveira et al, 2025). Peptides were identified and quantified by database searching against the *Mus musculus* reference proteome using Proteome Discoverer (version 2.5; Thermo Fisher Scientific, Villebon-sur-Yvette, France).

### Fluxomics analysis

Metabolites were analyzed by LC-HRMS. Cells were incubated for 24 h in serum-free DMEM lacking glutamine, pyruvate, and phenol red, supplemented with 25 mM 13C6-glucose (Eurisotop CLM-1396-1). After collection, cells were lysed in 200 µL absolute methanol and centrifuged at 12,000 rpm for 15 min. Supernatants were dried under nitrogen at 45 °C and resuspended in water containing 0.1% formic acid. Samples were analyzed using a Synapt G2 HRMS Q-TOF mass spectrometer (Waters) in negative ion mode, with separation on an Acquity H-Class UPLC system. Ionization parameters included a 1 kV capillary voltage, 30 V cone voltage, 900 L/h desolvation gas flow, and 550 °C temperature. Data were processed with MassLynx, TargetLynx, and IsoCor. Metabolite levels were normalized to cell number (per 10⁶ cells). 13C enrichment was calculated as the ratio of labeled to unlabeled metabolites. Mann–Whitney tests were used for statistical analysis (GraphPad Prism).

### Transmission electron microscopy (TEM)

Adipose tissue (1 mm³) or differentiated adipocytes were fixed for 16 h at 4 °C in 2.5% glutaraldehyde (LFG Distribution) in 0.1 M Sorensen buffer (pH 7.4). After rinsing in 0.2 M cacodylate buffer (pH 7.4), samples were post-fixed in 2% osmium tetroxide/1.5% potassium ferrocyanide for 45 min at room temperature. Samples were dehydrated in a graded ethanol series and embedded in EMbed 812 resin (LFG Distribution), polymerized at 60 °C for 48 h. Semi-thin (1 µm) sections were stained with methylene blue/Azur II and observed under an Olympus AX-60 light microscope. Ultrathin sections (60 nm) were examined with a Jeol JEM 1400 TEM operating at 120 keV.

### Transmission electron microscopy (TEM) analysis

Endoplasmic reticulum (ER), mitochondria, and lipid droplets (LD) were manually segmented on 2D TEM images using Fiji software. Contact sites were defined as membrane regions separated by a distance ≤50 nm. Individual contact segments are illustrated as C1, C2, and C3 in the schematic representation (Appendix Fig. S1F). For ER–mitochondria contacts (MAMs), two parameters were quantified: (1) contact number, defined as the occurrence of contacts with an intermembrane distance <50 nm, and (2) contact surface, defined as the cumulative contact length normalized to mitochondrial area. For mitochondria–LD (Mi–LD) contacts, the contact surface was calculated as the total contact length multiplied by 100 and normalized to mitochondrial area. The number of Mi–LD contacts corresponded to the number of discrete contact sites per mitochondrion. For ER–LD contacts, the contact surface was calculated as the total contact length multiplied by 100 and normalized to the LD area. The number of ER–LD contacts corresponded to the number of discrete contact sites per lipid droplet.

To investigate tripartite contact sites involving ER, mitochondria, and LDs, mitochondria were categorized according to their proximity to LDs. Mitochondria located within 50 nm of an LD were classified as peri-droplet mitochondria (PDM), whereas mitochondria located more than 50 nm away from LDs were classified as cytosolic mitochondria (CM). Contact surface and contact number were then quantified for each mitochondrial subset. ER–mitochondria contacts associated with PDM were designated MAM-LD, whereas those associated with CM were termed MAM-CM. This stratification enabled precise assessment of MAM remodeling according to mitochondrial spatial proximity to LDs.

Normalization of contact length to mitochondrial area (for MAM and Mi–LD contact surfaces) was performed to account for variability in mitochondrial size and sectioning plane inherent to 2D TEM imaging, thereby enabling comparison of contact extent independently of mitochondrial morphology.

### Western blotting

Cells or tissues were lysed in buffer containing 1% Igepal CA-630, 0.05–0.5% sodium deoxycholate, 0.1% SDS, 250 mM Tris-HCl (pH 7.5), and 150 mM NaCl, supplemented with protease inhibitors. Lysates were centrifuged at 13,000×*g* for 10 min at 4 °C. Equal amounts of protein were resolved by SDS-PAGE, transferred to membranes, and blocked in 5% milk in TBS-T (0.1% Tween-20) for 1 h at room temperature. Membranes were incubated with primary antibodies overnight at 4 °C and with HRP-conjugated secondary antibodies for 1 h at room temperature. Detection was performed using ECL substrate and visualized with a ChemiDoc system (Bio-Rad). Band intensities were quantified using ImageJ FIJI and normalized to total protein content (Stain-Free system, Bio-Rad).

### RNA analysis

Total RNA from tissues was purified using Trizol (Life technology, les Ulis, France) according to the manufacturer’s instructions. Total RNA from cell samples were purified with the NucleoSpin RNA II (Macherey Nagel, Hoerdt, France). Total RNA (500 ng) was reverse-transcribed, and real-time quantitative PCR was performed using the TaqMan 7900 Sequence Detection System (Applied Biosystems, Warrington, UK). Primers were designed using the Primer Express 3.1 software (sequence available on request).

### Statistical analysis

Data were expressed as mean ± SEM. Parametric or nonparametric tests are specified in the figure legends. Area under the curve (AUC) values were compared using unpaired *t*-tests. Two-way ANOVA was used, where indicated, to test for interactions between experimental groups and variables. All analyses were performed using GraphPad Prism.

## Supplementary information


Appendix
Peer Review File
Movie EV1
Movie EV2
Movie EV3
Movie EV4
Source data Fig. 1
Source data Fig. 2
Source data Fig. 3
Source data Fig. 4
Source data Fig. 5
Source data Fig. 6
Source data Fig. 7
Expanded View Figures


## Data Availability

Lipid droplets segmentation—The pipeline used for LD segmentation is available here: https://gitlab.univ-nantes.fr/micropicell/analyseimage/using-cellpose-and-cellstitch-for-lipid-droplets-segmentations. Proteomic raw data were available here: https://doi.org/10.5281/zenodo.20398187 (Prieur, 2026). The source data of this paper are collected in the following database record: biostudies:S-SCDT-10_1038-S44318-026-00876-z.
