# Supplementary material for: Tripartite ER-mitochondria-lipid droplets contact sites control adipocyte metabolic flexibility
Source: EMBO J. 2026 Jul 22;45(17):6021–53. doi: 10.1038/s44318-026-00876-z (PMC13534470; doi:10.1038/s44318-026-00876-z)
Supplement: Supplementary file 1 [file 44318_2026_876_MOESM14_ESM.pdf]

Expanded View Figures

Figure EV1. Seipin deficiency alters adipose tissue organelle organization and mitochondrial function.

(**A**) *Bscl2* mRNA expression was quantified by RT-qPCR in inguinal adipose tissue from *Bscl2*$^{lox/lox}$ (Control) and *Bscl2*$^{lox/lox}$*ERT2-Adipoq-Cre* (iATSKO) mice collected in the fed state, 14 days after tamoxifen injection (100 mg/kg). (**B–D**) TEM analyses were performed to quantify the number of MAMs (**B**), Mi–LD contacts (**C**), and ER–LD contacts (**D**) per cell. (**E**) Mitochondrial aspect ratio was quantified using Fiji. (**F**) Seahorse analysis was performed on 2 mg explants of subcutaneous adipose tissue. (**G**) Mitochondrial DNA content was quantified by measuring mitochondrial cytochrome *b* DNA normalized to genomic β-actin DNA. (**H**, **I**) Mitochondrial area and length were quantified according to mitochondrial subtype, together with their relative distribution (**I**). (**J**) Lipid droplet surface area was quantified using PLIN1 fluorescence signal. Data information: (**A**) Bars represent mean ± SEM; $n > 12$ mice per genotype. (**B–D**) Box plots show the median (center line), the 25th and 75th percentiles (box), the minimum and maximum values (whiskers), and individual data points; measurements were obtained from two mice per genotype, with five cells analyzed per mouse. (**F**) Seahorse analysis was performed using three biopsies per mouse; $n = 6$ mice per genotype. (**G**) $n = 6$ mice per genotype. (**H**) Violin plots show the distribution of quantified mitochondria, with each dot representing one individual mitochondrion; median indicated by the central line; measurements were obtained from two mice per genotype, with five cells analyzed per mouse. (**I**) Bars: mean ± SD, with each dot representing a cell. Measurements were obtained from two mice per genotype, with five cells analysed per mouse. $p < 0.05$, $p < 0.001$, $p < 0.0001$, One-way ANOVA test. (**J**) Violin plots show the distribution of lipid droplet surface area, with each dot representing one lipid droplet; median indicated by the central line; measurements were obtained from three mice per genotype, with 25 cells analyzed per mouse. Statistical analysis: Mann–Whitney test (**A–H**); one-way ANOVA (**I**, **J**). Exact *P* values from two-tailed Mann–Whitney tests: $P = 0.0027$ (**C**) and $P = 0.0016$ (**D**). Exact adjusted *P* values from Tukey's multiple comparisons test: contact area, PDM CTL vs. PDM iATSKO, $P = 0.0168$; contact length, PDM CTL vs. PDM iATSKO, $P = 0.0020$ (**H**). All other significant comparisons, $P < 0.001$. $^{*}p < 0.05$, $^{**}p < 0.01$, $^{***}p < 0.001$, $^{****}p < 0.0001$.

© The Author(s)

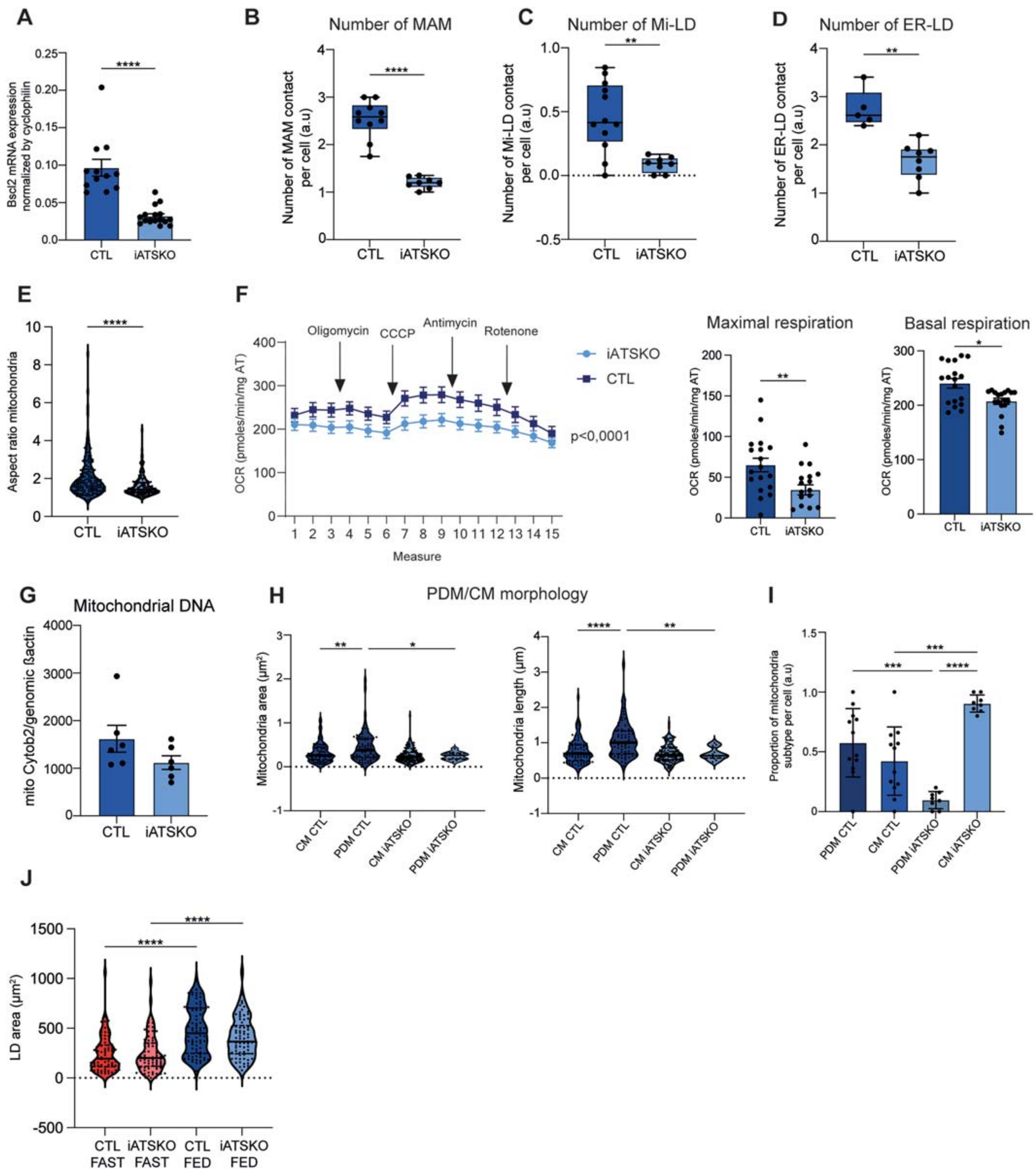
A
Bscl2 mRNA expression normalized by cyclophilin
CTL iATSKO
B
Number of MAM
Number of MAM contact per cell (a.u)
CTL iATSKO
C
Number of Mi-LD
Number of Mi-LD contact per cell (a.u)
CTL iATSKO
D
Number of ER-LD
Number of ER-LD contact per cell (a.u)
CTL iATSKO
E
Aspect ratio mitochondria
CTL iATSKO
F
Oligomycin
CCCP
Antimycin
Rotenone
OCR (pmoles/min/mg AT)
Measure
iATSKO
CTL
p<0,0001
Maximal respiration
Basal respiration
OCR (pmoles/min/mg AT)
CTL iATSKO
G
Mitochondrial DNA
mito Cytob2/genomic ßactin
CTL iATSKO
H
PDM/CM morphology
Mitochondria area (µm²)
Mitochondria length (µm)
CM CTL PDM CTL CM iATSKO PDM iATSKO
I
Proportion of mitochondria subtype per cell (a.u)
PDM CTL CM CTL PDM iATSKO CM iATSKO
J
LD area (µm²)
CTL FAST iATSKO FAST CTL FED iATSKO FED

 © The Author(s)

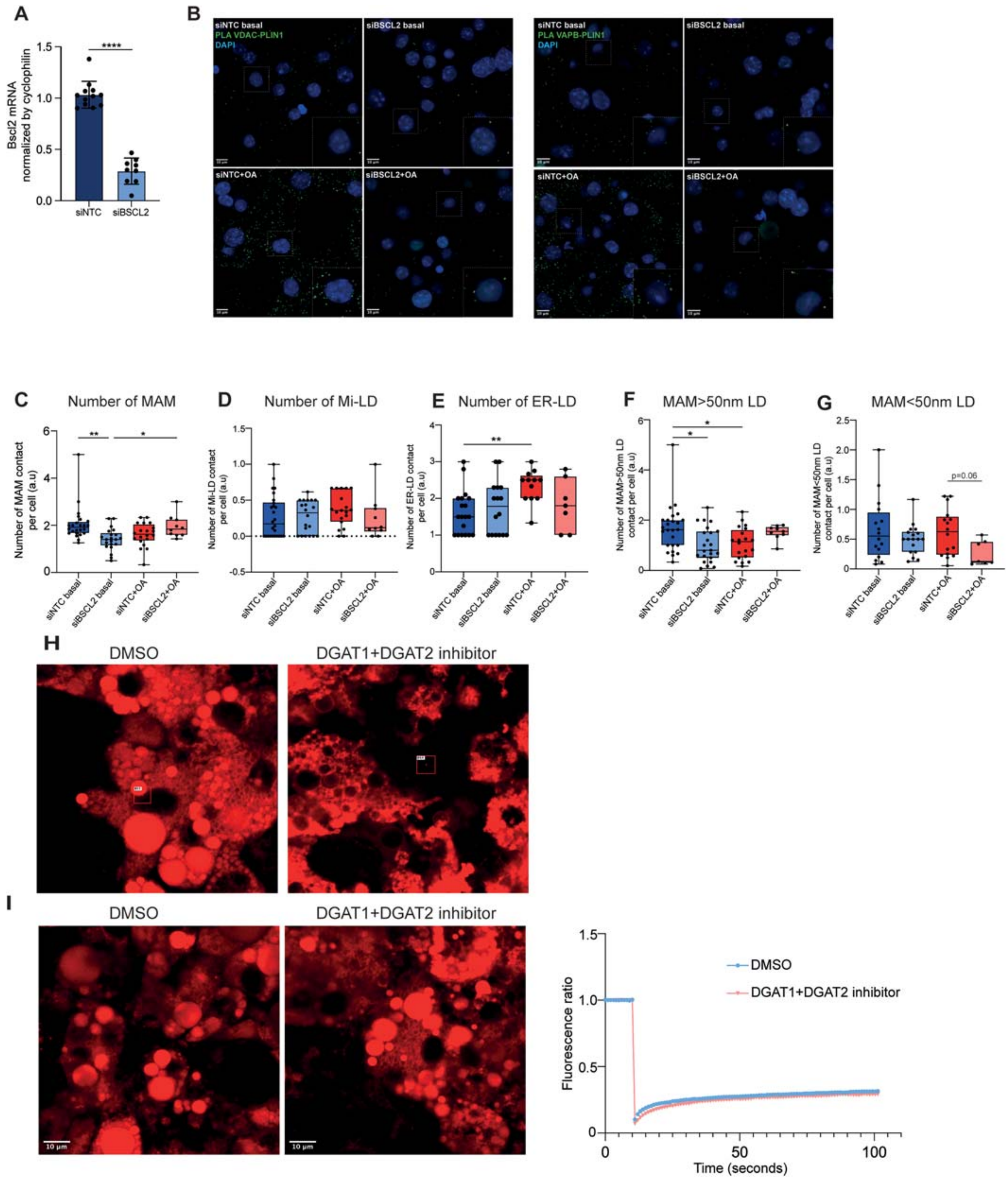
A
Bscl2 mRNA normalized by cyclophilin
****
siNTC
siBSCL2
B
siNTC basal
PLA VDAC-PLIN1
DAPI
siBSCL2 basal
siNTC+OA
siBSCL2+OA
siNTC basal
PLA VAPB-PLIN1
DAPI
siBSCL2 basal
siNTC+OA
siBSCL2+OA
C
Number of MAM
Number of MAM contact per cell (a.u)
D
Number of Mi-LD
Number of Mi-LD contact per cell (a.u)
E
Number of ER-LD
Number of ER-LD contact per cell (a.u)
F
MAM>50nm LD
Number of MAM>50nm LD contact per cell (a.u)
G
MAM<50nm LD
Number of MAM<50nm LD contact per cell (a.u)
p=0.06
siNTC basal
siBSCL2 basal
siNTC+OA
siBSCL2+OA
H
DMSO
DGAT1+DGAT2 inhibitor
I
DMSO
DGAT1+DGAT2 inhibitor
10 µm
Fluorescence ratio
Time (seconds)
DMSO
DGAT1+DGAT2 inhibitor

© The Author(s)

Figure EV2. Seipin depletion alters organelle contacts and lipid flux in adipocytes.

(**A**) *Bscl2* mRNA expression was quantified by RT-qPCR in 3T3-L1 adipocytes transfected with control (siNTC) or *BSCL2* siRNA (siBSCL2). (**B**) Representative PLA images of VDAC/PLIN1 and VAPB/PLIN1 in control and *BSCL2*-depleted adipocytes under basal or oleate-loaded conditions. (**C–G**) TEM analyses were performed to quantify the number of MAMs (**C**), Mi-LD contacts (**D**), ER-LD contacts (**E**), MAM-CM (>50 nm from LDs) (**F**), and MAM-LD (<50 nm from LDs) (**G**). (**H**) 3T3-L1 adipocytes were incubated for 6 h with BODIPY-C12 and treated with DMSO or DGAT1/2 inhibitors. (**I**) FRAP analysis was performed in adipocytes treated with DMSO or DGAT1/2 inhibitors added immediately before acquisition. Data information: (**A**) Bars represent mean ± SEM; triplicate measurements from three independent experiments. (**C–G**) Box plots show the median (center line), the 25th and 75th percentiles (box), the minimum and maximum values (whiskers), and individual data points; measurements were obtained from >10 cell profiles per condition. Statistical analysis: Mann–Whitney test (**A**); two-way ANOVA (**C–G**). Exact adjusted *P* values from multiple comparisons test: siNTC basal vs. siBSCL2 basal, $P = 0.0057$; siBSCL2 basal vs. siBSCL2 + OA, $P = 0.0436$ (**C**). siNTC basal vs. siNTC + OA, $P = 0.0145$ (**E**). siNTC basal vs. siBSCL2 basal, $P = 0.0209$; siNTC basal vs. siNTC + OA, $P = 0.0447$ (**F**). All other significant comparisons, $P < 0.001$. *$p < 0.05$, **$p < 0.01$, ***$p < 0.001$, ****$p < 0.0001$.

© The Author(s)

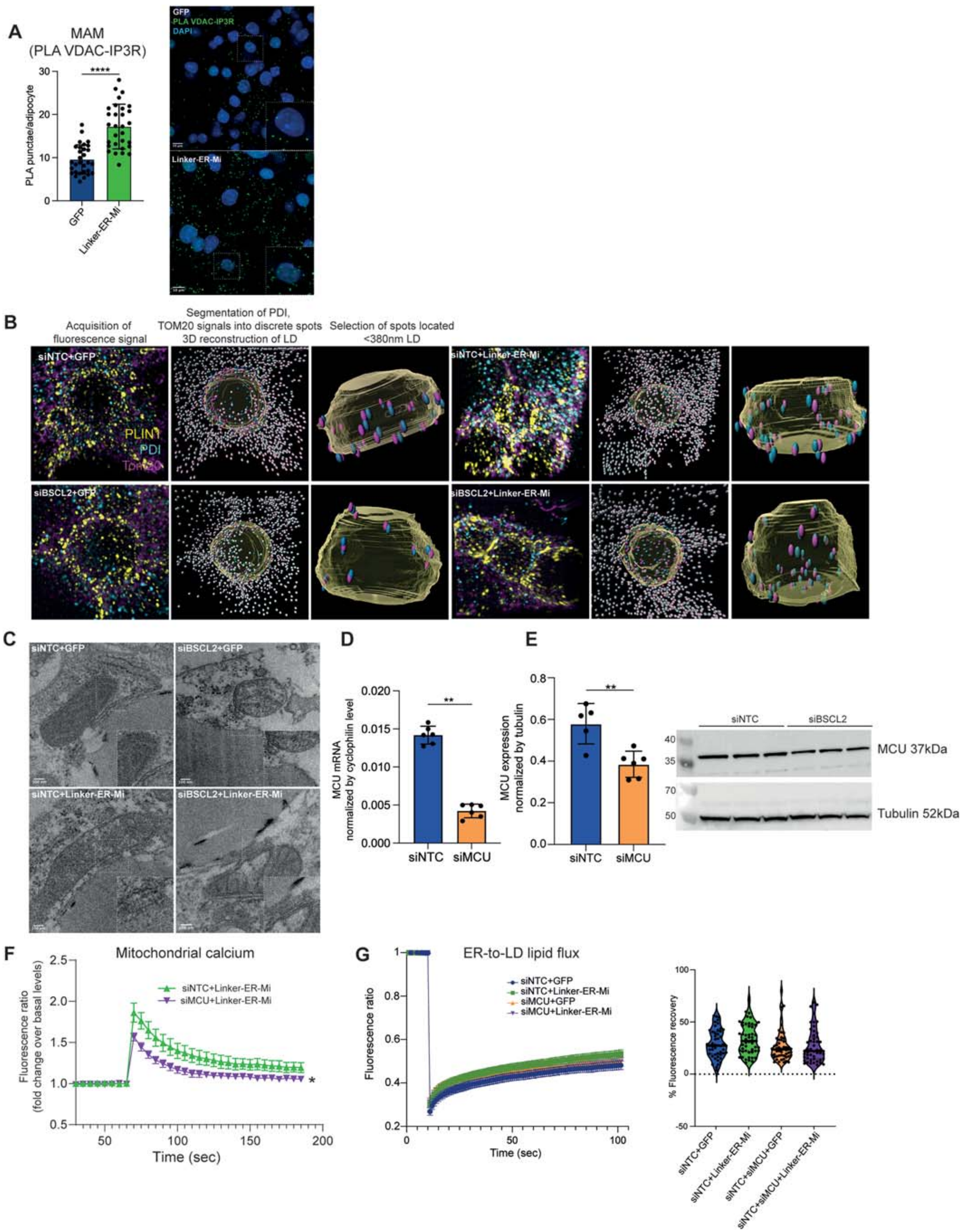
A
MAM
(PLA VDAC-IP3R)
PLA punctae/adipocyte
****
GFP
Linker-ER-Mi
PLA VDAC-IP3R
DAPI
B
Acquisition of fluorescence signal
Segmentation of PDI, TOM20 signals into discrete spots 3D reconstruction of LD
Selection of spots located <380nm LD
siNTC+GFP
siNTC+Linker-ER-Mi
siBSCL2+GFP
siBSCL2+Linker-ER-Mi
PLIN1
PDI
Tom20
C
siNTC+GFP
siBSCL2+GFP
siNTC+Linker-ER-Mi
siBSCL2+Linker-ER-Mi
D
MCU mRNA normalized by cyclophilin level
**
siNTC
siMCU
E
MCU expression normalized by tubulin
**
siNTC
siMCU
siNTC
siBSCL2
MCU 37kDa
Tubulin 52kDa
F
Mitochondrial calcium
Fluorescence ratio (fold change over basal levels)
siNTC+Linker-ER-Mi
siMCU+Linker-ER-Mi
Time (sec)
G
ER-to-LD lipid flux
Fluorescence ratio
siNTC+GFP
siNTC+Linker-ER-Mi
siMCU+GFP
siMCU+Linker-ER-Mi
Time (sec)
% Fluorescence recovery
siNTC+GFP
siNTC+Linker-ER-Mi
siNTC+siMCU+GFP
siNTC+siMCU+Linker-ER-Mi

© The Author(s)

Figure EV3. ER–mitochondria tethering and mitochondrial calcium handling in adipocytes.

(**A**) 3T3-L1 adipocytes were infected with adenoviruses expressing GFP or Linker-ER-Mi, and ER–mitochondria contacts were quantified by VDAC/IP3R PLA. (**B**) Structured illumination microscopy (SIM) and Imaris analysis workflow used to quantify ER–mitochondria contacts in proximity to lipid droplets. Selection of spots representation are reuse of Fig. 3A. (**C**) Representative TEM images of 3T3-L1 adipocytes transfected with control or *BSCL2* siRNA and infected with GFP or Linker-ER-Mi adenoviruses. (**D**) *MCU* mRNA expression was quantified by RT-qPCR following siRNA-mediated depletion. (**E**) Representative immunoblots of MCU and actin with corresponding quantification. (**F**) Mitochondrial calcium uptake was monitored using caged IP_3 photorelease and Rhod-2 fluorescence. (**G**) FRAP analysis was performed to assess fluorescence recovery kinetics. Data information: (**A**) Bars represent mean ± SD; two independent biological experiments, with 15 fields acquired per condition per experiment and ~50 cells analyzed per field (~1500 cells per condition). (**D**) Bars represent mean ± SEM; triplicate measurements from two independent experiments. (**E**) Bars represent mean ± SD; triplicate measurements from two independent experiments. (**F**) $n > 60$ cells per condition from 3 independent experiments. (**G**) Violin plots show fluorescence recovery (%), with each dot representing one cell; median and quartiles are indicated; $n > 45$ cells per condition from three independent experiments. Statistical analysis: Mann–Whitney test (**A**, **D**, **E**); two-way ANOVA (**F**); one-way ANOVA (**G**). Exact *P* values from two-tailed Mann–Whitney tests: $P = 0.0022$ (**D**) and $P = 0.0087$ (**E**). All other significant comparisons, $P < 0.001$. *$p < 0.05$, **$p < 0.01$, ***$p < 0.001$, ****$p < 0.0001$.

 © The Author(s)

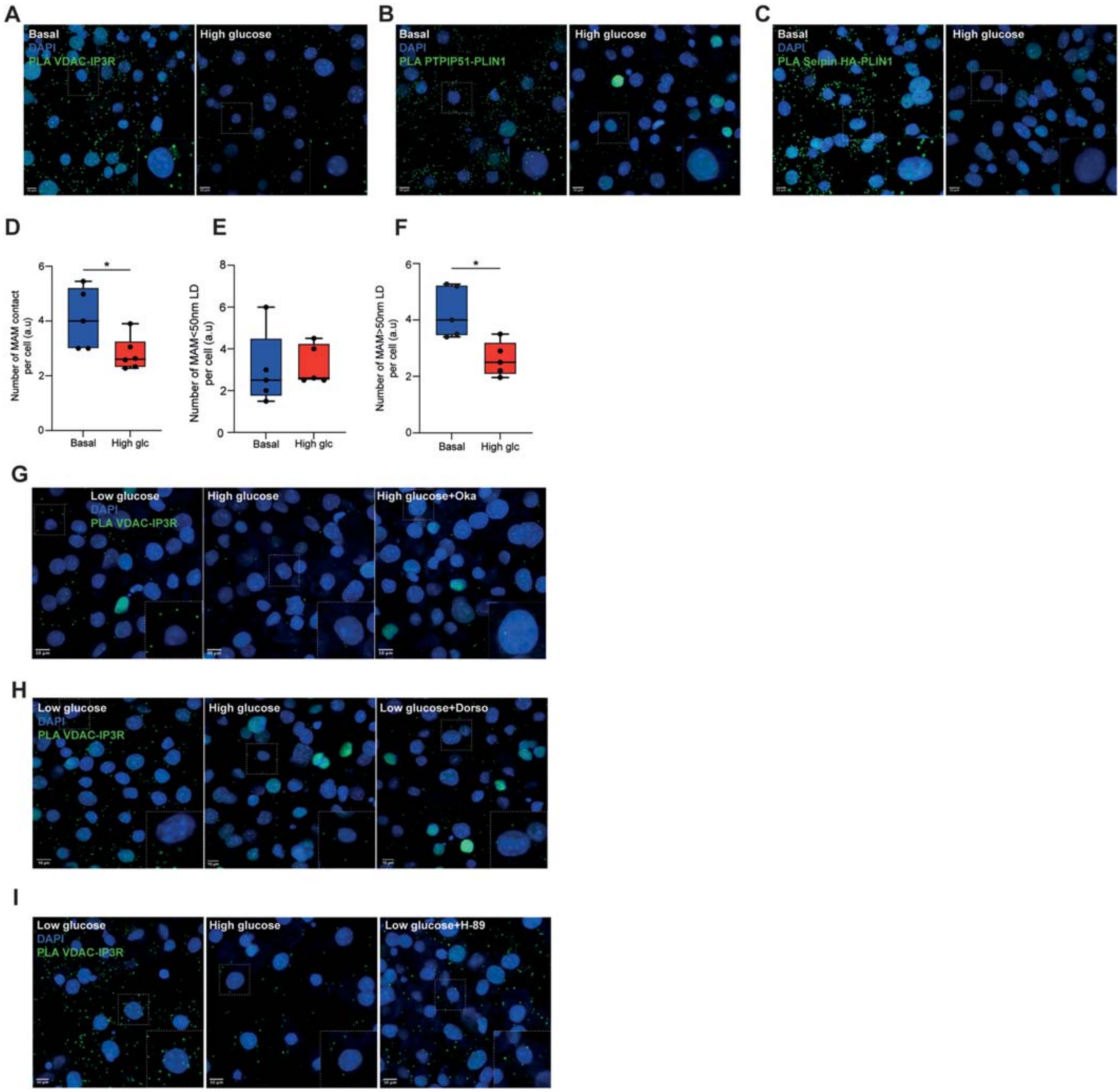

Figure EV4. Nutrient availability regulates membrane contact-site organization in adipocytes.

(**A–C**) Representative PLA images of ER–mitochondria contacts (VDAC/IP3R; **A**), Mi–LD contacts (PTPIP51/PLIN1; **B**), and ER–LD contacts (Seipin-HA/PLIN1; **C**) in adipocytes cultured under basal (6 h, 5 mM glucose) or high-glucose (6 h, 25 mM glucose) conditions. (**D–F**) TEM analyses were performed to quantify the number of MAMs (**D**), MAM–LD (<50 nm from LDs) (**E**), and MAM–CM (>50 nm from LDs) (**F**) per cell. (**G–I**) Representative PLA images of MAMs (VDAC/IP3R) in adipocytes cultured under low-glucose conditions alone or treated with dorsomorphin (5 µM) or H-89 (10 µM). Data information: (**D–F**) Box plots show the median (center line), the 25th and 75th percentiles (box), the minimum and maximum values (whiskers), and individual data points. Measurements were obtained from >5 cell profiles per condition. Statistical analysis: Mann–Whitney tests. Exact *P* values from two-tailed Mann–Whitney tests: $P = 0.0455$ (**D**) and $P = 0.0238$ (**F**). $^{*}p < 0.05$.

© The Author(s)

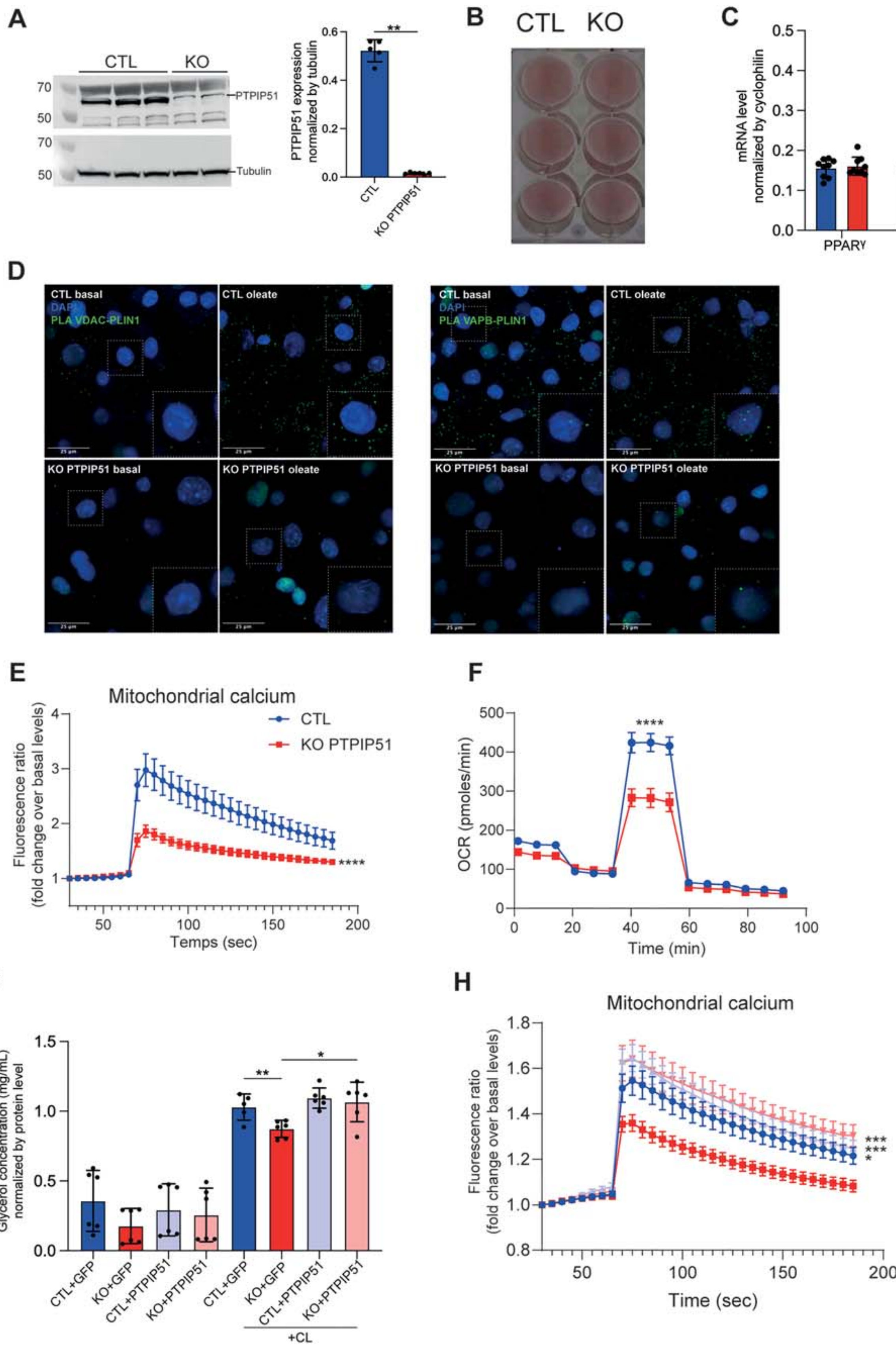
A
CTL
KO
70
50
70
50
PTPIP51
Tubulin
PTPIP51 expression normalized by tubulin
**
CTL
KO PTPIP51
B
CTL KO
C
mRNA level normalized by cyclophilin
****
PPARγ
FAS
ACLY
D
CTL basal
DAPI
PLA VDAC-PLIN1
CTL oleate
KO PTPIP51 basal
KO PTPIP51 oleate
PLA VAPB-PLIN1
E
Mitochondrial calcium
CTL
KO PTPIP51
Fluorescence ratio (fold change over basal levels)
Temps (sec)
****
F
OCR (pmoles/min)
Time (min)
****
G
Glycerol concentration (mg/mL) normalized by protein level
**
*
CTL+GFP
KO+GFP
CTL+PTPIP51
KO+PTPIP51
+CL
H
Mitochondrial calcium
Fluorescence ratio (fold change over basal levels)
Time (sec)
***
***
*

 © The Author(s)

Figure EV5. PTPIP51 depletion alters adipocyte differentiation and mitochondrial function.

(**A**) Representative immunoblots of PTPIP51 and tubulin in control and PTPIP51 KO 3T3-L1 adipocytes, with corresponding quantification. (**B**) Oil Red O staining of control and PTPIP51 KO 3T3-L1 adipocytes. (**C**) *PPARγ*, *FAS*, and *ACLY* mRNA expression levels were quantified by RT-qPCR in control and PTPIP51 KO adipocytes. (**D**) Representative PLA images of Mi–LD contacts (VDAC/PLIN1) and ER–LD contacts (VAPB/PLIN1) under basal or oleate-loaded conditions in control and PTPIP51 KO adipocytes. (**E**) Mitochondrial calcium uptake was monitored using caged IP_3 photorelease and Rhod-2 fluorescence. (**F**) Oxygen consumption rate (OCR) was measured using Seahorse analysis in control and PTPIP51 KO adipocytes. (**G**) Lipolysis was assessed by measuring glycerol release into the extracellular medium following 2 h incubation in the presence or absence of CL316-243. (**H**) Mitochondrial calcium uptake was monitored as described above. Data information: (**A**) Bars represent mean ± SD; triplicate measurements from two independent experiments. (**C**) Bars represent mean ± SD; triplicate measurements from three independent experiments. (**E**, **F**, **H**) Bars represent mean ± SEM; $n = 3$ independent experiments. (**G**) Bars represent mean ± SD; $n = 2$ independent experiments. Statistical analysis: Mann–Whitney test (**A**); one-way ANOVA (**C**); two-way ANOVA (**E**, **G**, **H**). Exact *P* value from a two-tailed Mann–Whitney test: $P = 0.0043$ (**A**). Exact adjusted *P* values from Dunn's multiple comparisons test (**G**): KO vs. CTL + CL, $P = 0.0228$; KO vs. CTL + PTPIP51 + CL, $P = 0.0018$; KO vs. KO + PTPIP51 + CL, $P = 0.0018$; CTL + PTPIP51 vs. CTL + PTPIP51 + CL, $P = 0.0308$; CTL + PTPIP51 vs. KO + PTPIP51 + CL, $P = 0.0308$; KO + PTPIP51 vs. CTL + PTPIP51 + CL, $P = 0.0105$; KO + PTPIP51 vs. KO + PTPIP51 + CL, $P = 0.0105$. Two-way repeated-measures ANOVA: time × group interaction, $P = 0.0018$; group effect, $P = 0.0025$ (**H**). $^{*}p < 0.05$, $^{**}p < 0.01$, $^{***}p < 0.001$, $^{****}p < 0.001$.

© The Author(s)
