# Supplementary material for: Tripartite ER-mitochondria-lipid droplets contact sites control adipocyte metabolic flexibility
Source: EMBO J. 2026 Jul 22;45(17):6021–53. doi: 10.1038/s44318-026-00876-z (PMC13534470; doi:10.1038/s44318-026-00876-z)
Supplement: Supplementary file 2 [file 44318_2026_876_MOESM1_ESM.pdf]

Appendix for :

Tripartite ER-Mitochondria-Lipid Droplets contact sites control adipocyte metabolic flexibility

Appendix Content	Page(s)
Appendix Figure S1	2
Appendix Figure S2	3
Appendix Figure S3	4–5
Appendix Figure S4	6–7
Appendix Figure S5	8
Appendix Figure S6	9
Appendix Figure S7	10
Appendix Figure S8	11–12
Appendix Figure S9	13–14

Appendix figure S1

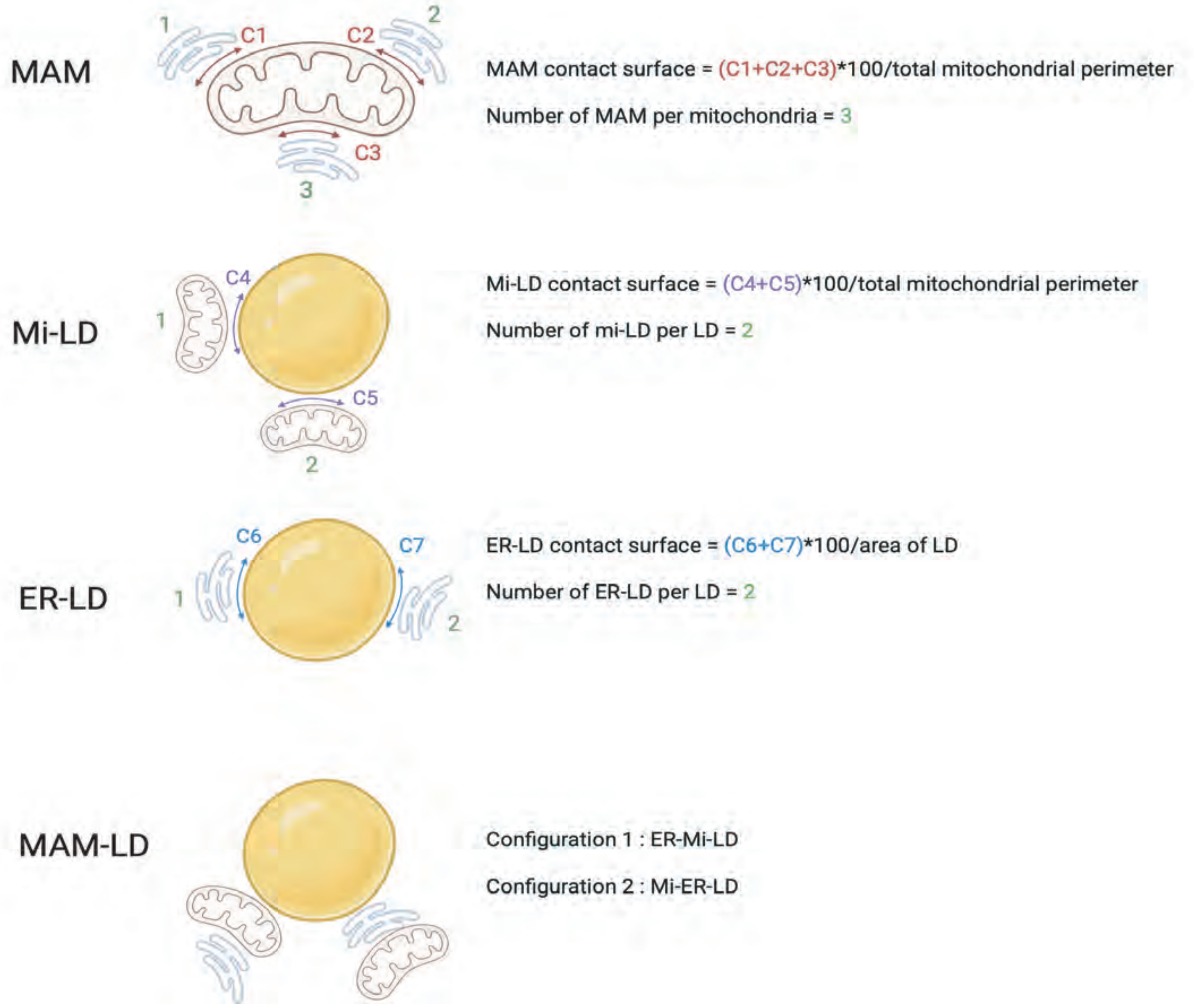

Appendix Figure S1
The cartoon represents the metrics that have been measured for each contact site and how they were obtained.

Appendix figure S2

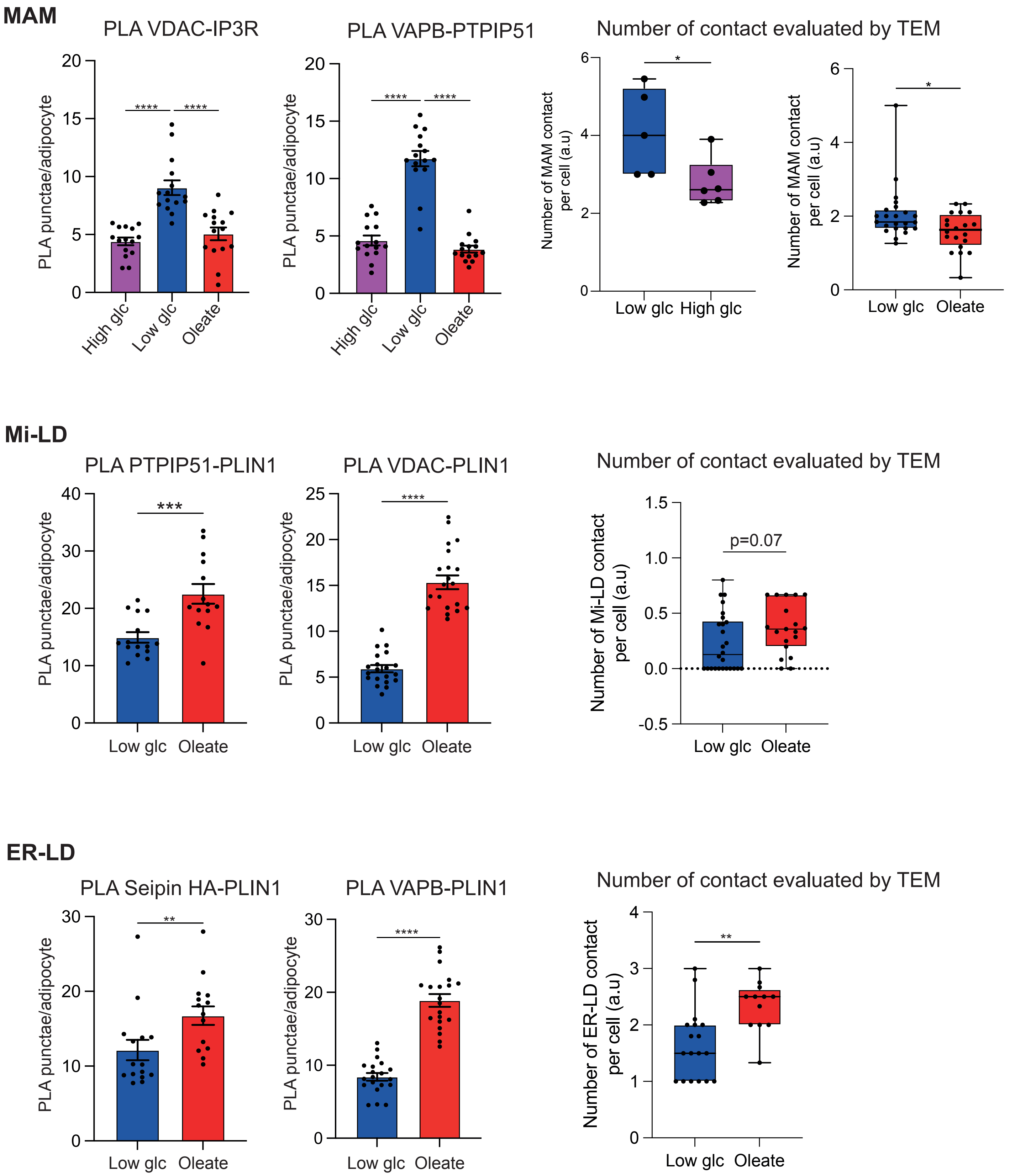

Appendix Figure S2

3T3-L1 adipocytes differentiated for 7 days were incubated in low-glucose medium in the presence or absence of oleic acid for 6 hours. Two PLA probe pairs were used to assess each type of inter-organelle contact, including MAM, mitochondria–lipid droplet (Mi–LD), and ER–lipid droplet (ER–LD) interactions. The right panel presents the number of contacts quantified by TEM.

Appendix figure S3

A

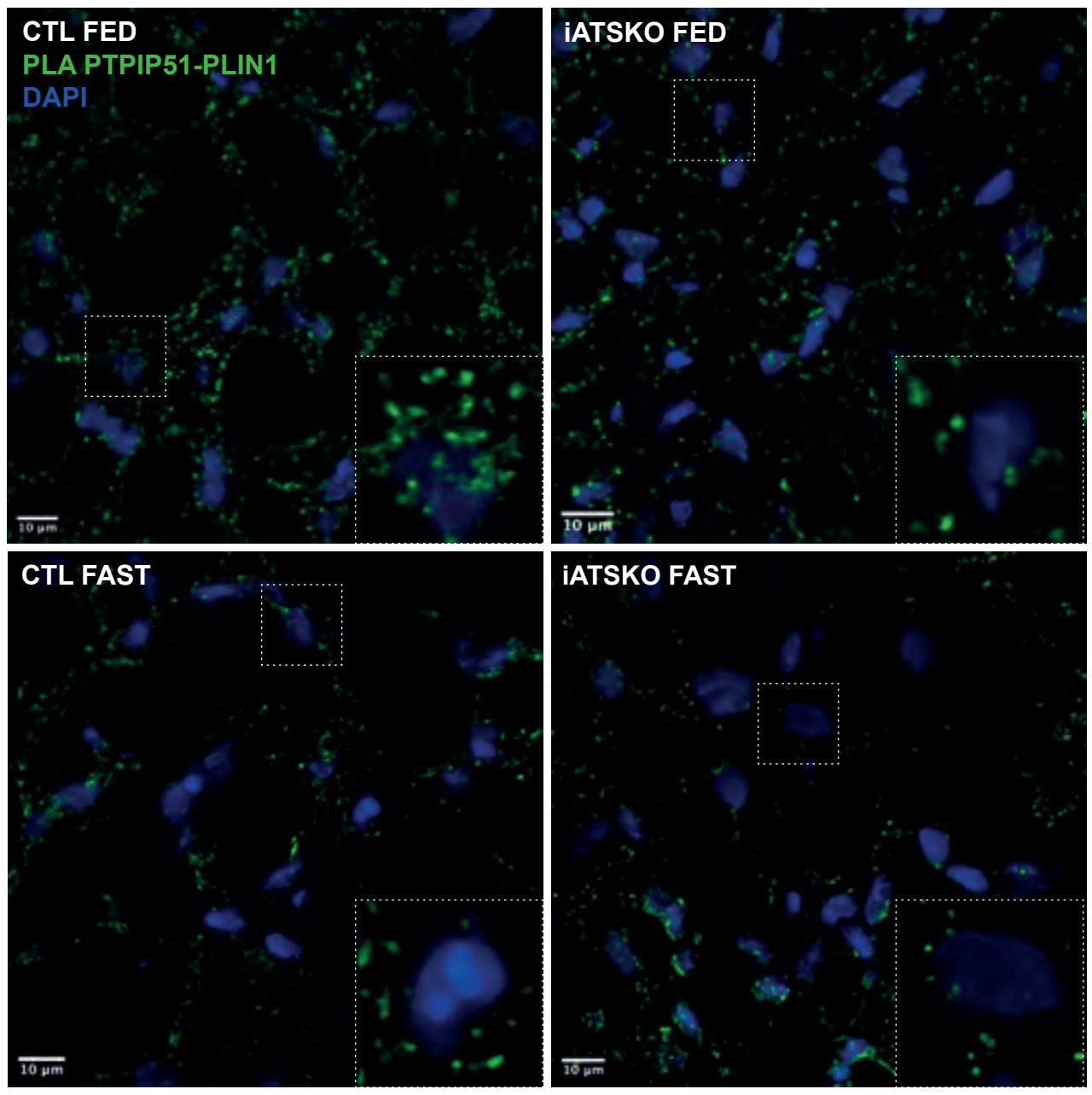

B

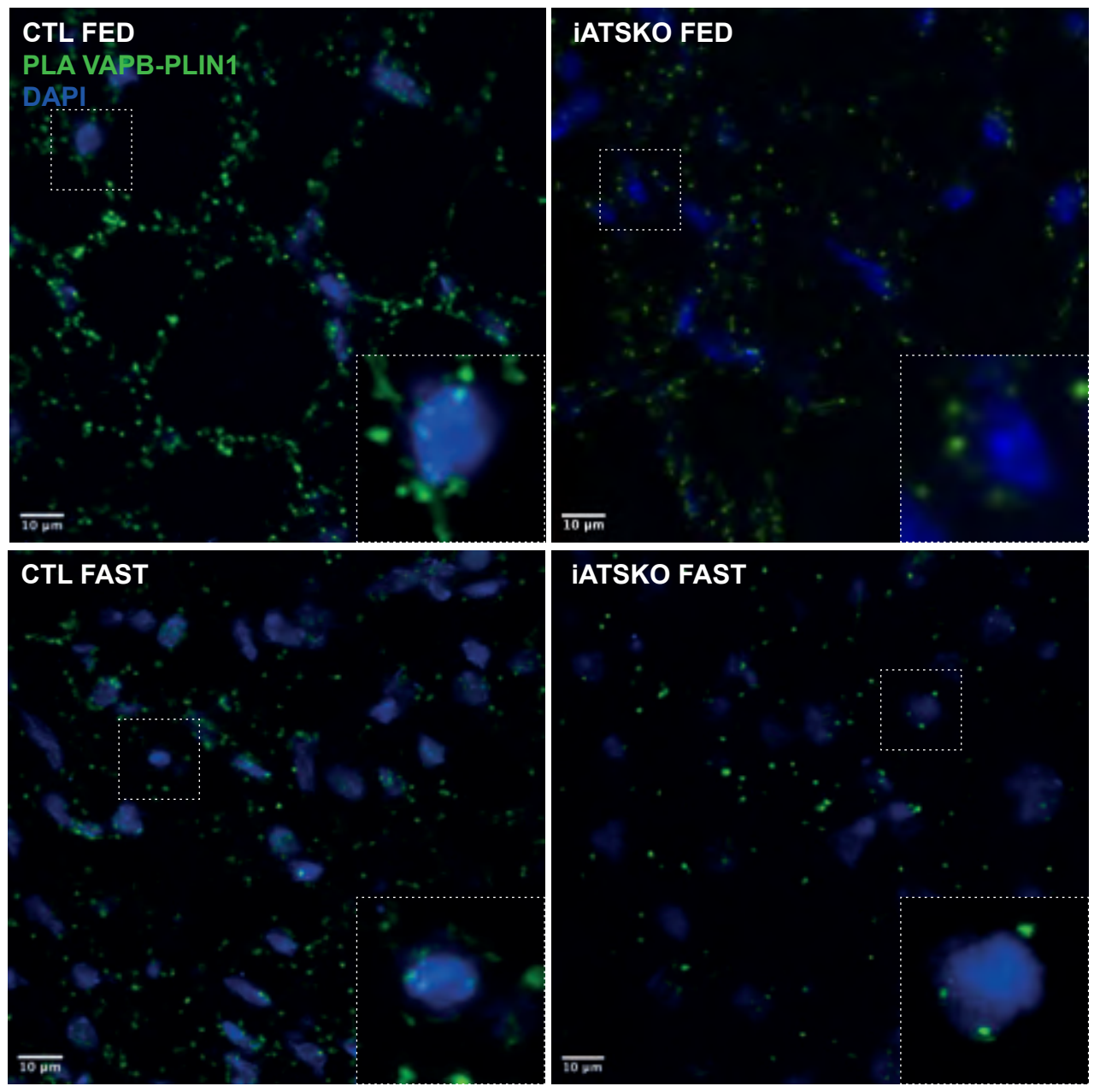

C

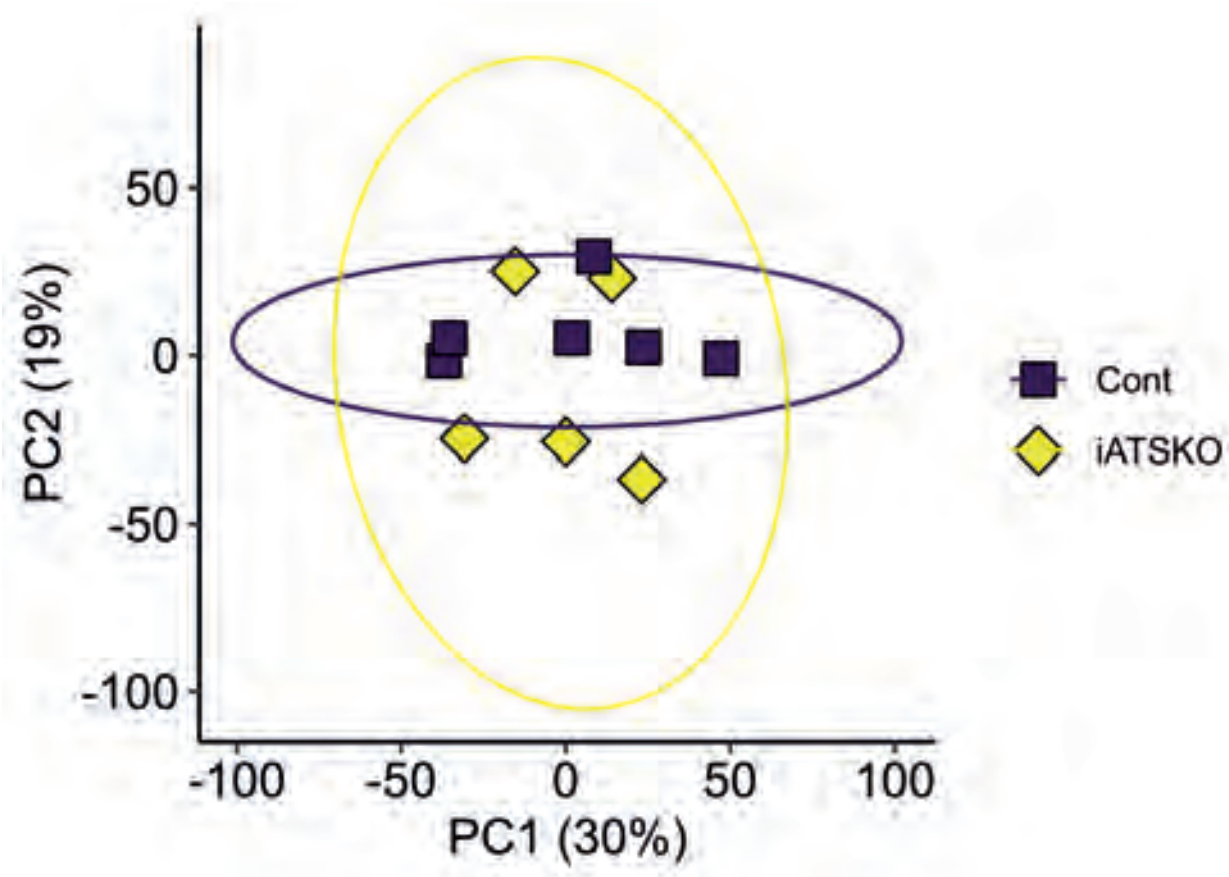

D

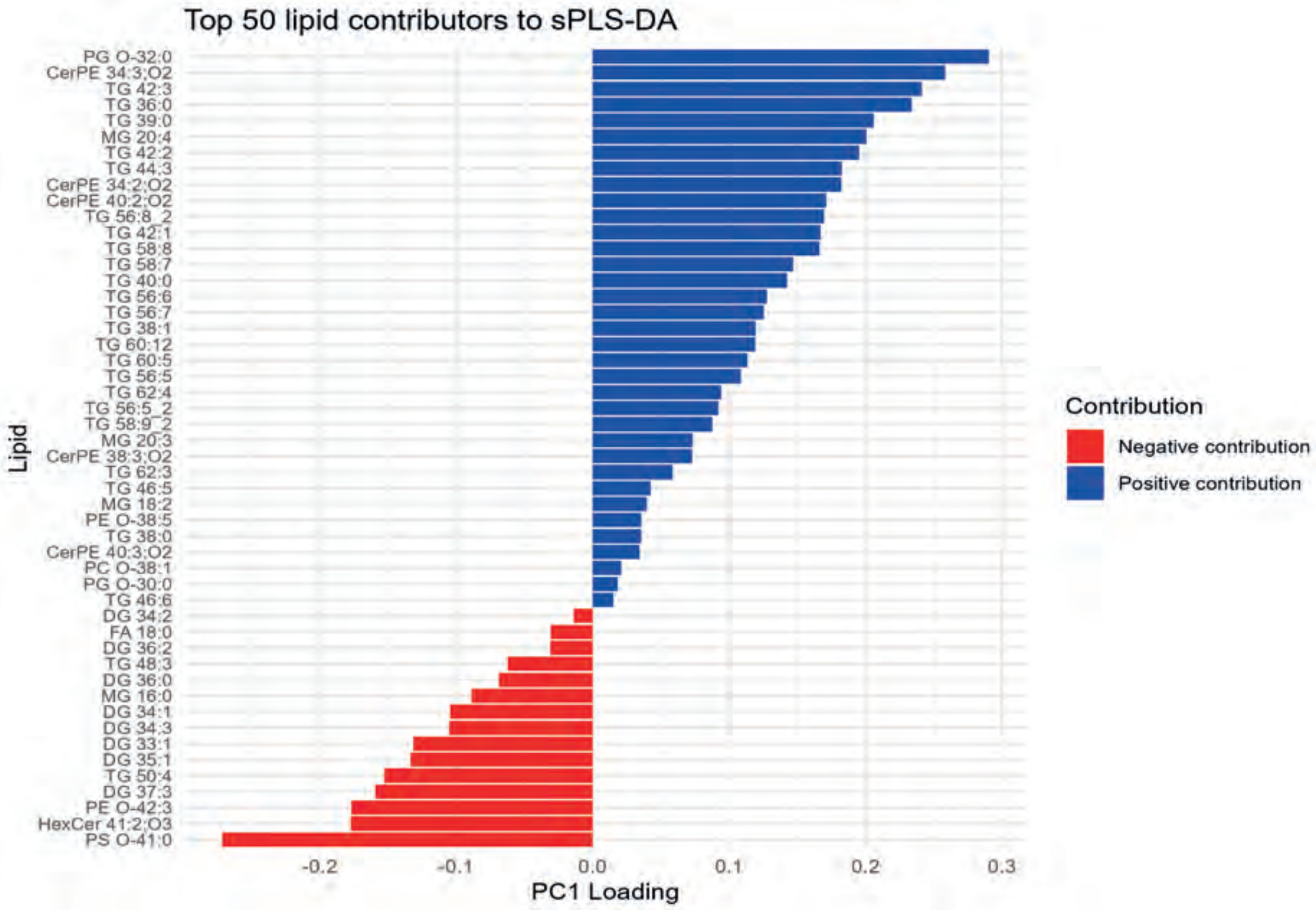

Appendix Figure S3

Representative images of PLA PTPIP51-PLIN1 and VAPB-PLIN1 on CTL and iATSKO adipose tissue sections (A-B). LDs were isolated from iATSKO and control mice, and proteomic (C) and lipidomic (D) analyses were performed. sPLDA analysis identified the lipids that contributed the most to the separation of the genotype profiles (D).

Appendix figure S4

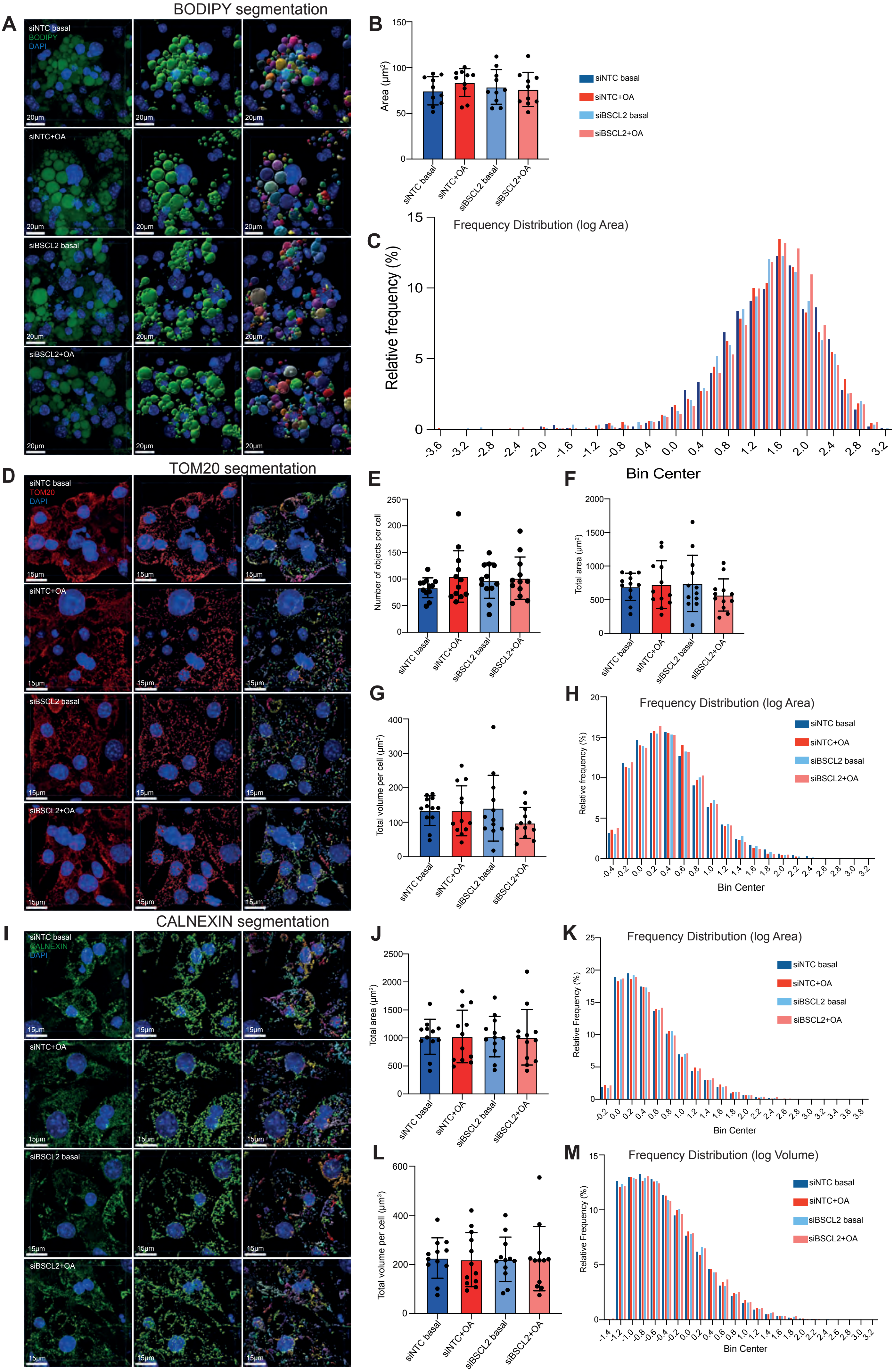

Appendix Figure S4
3T3-L1 adipocytes differentiated for 7 days were reverse-transfected with the indicated siRNAs. After 72 h, cells were incubated in low-glucose medium ± oleic acid for 6 h. Cells were stained with BODIPY to visualize LDs (E), anti-Tom20 to label mitochondria, and anti-calnexin to label the ER (F). Quantitative analyses were performed using Imaris software. For each condition, experiments were performed in duplicate wells, and a total of 15 images, representing approximately 150 cells, were analyzed per condition.

Appendix figure S5

A

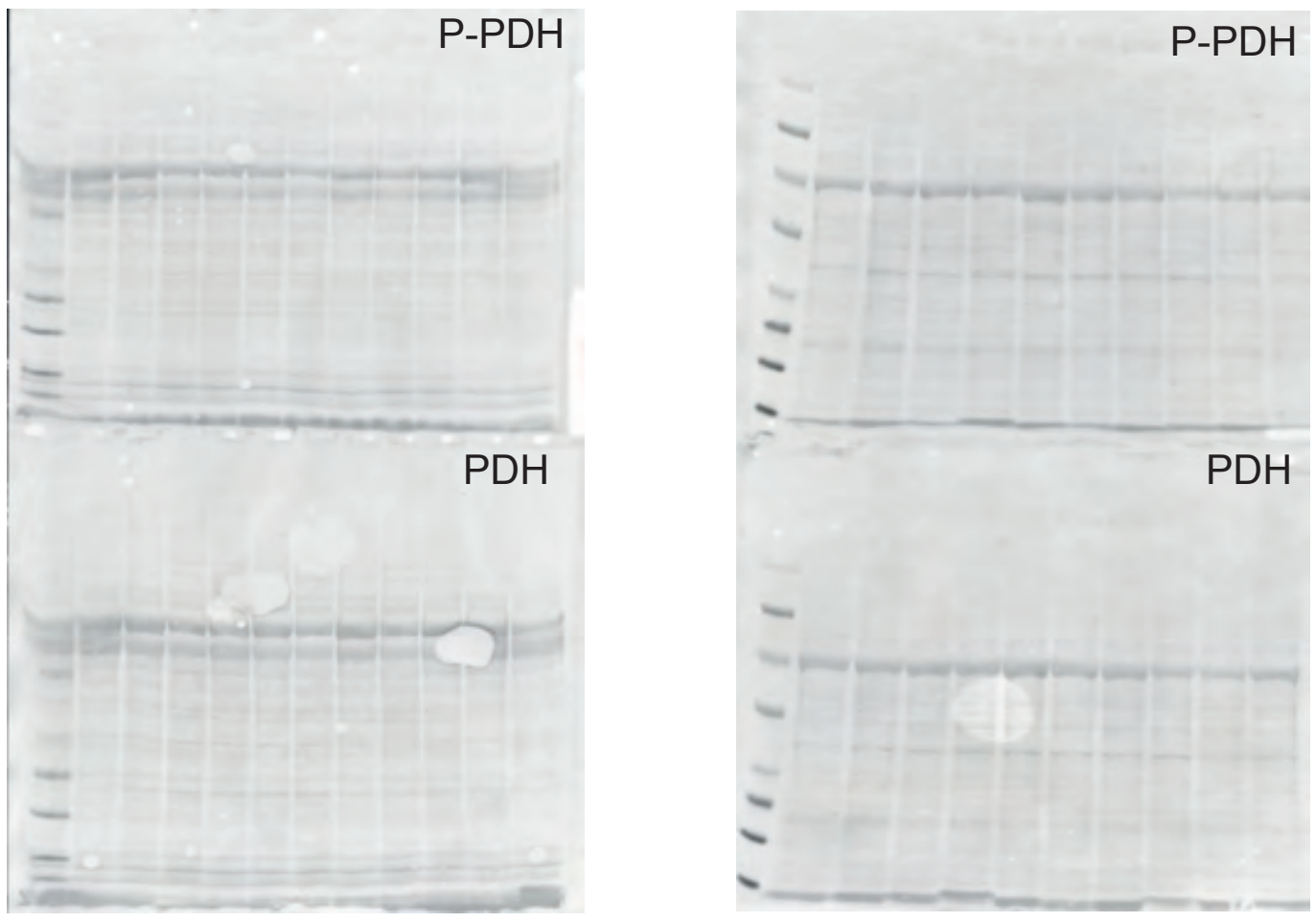

B

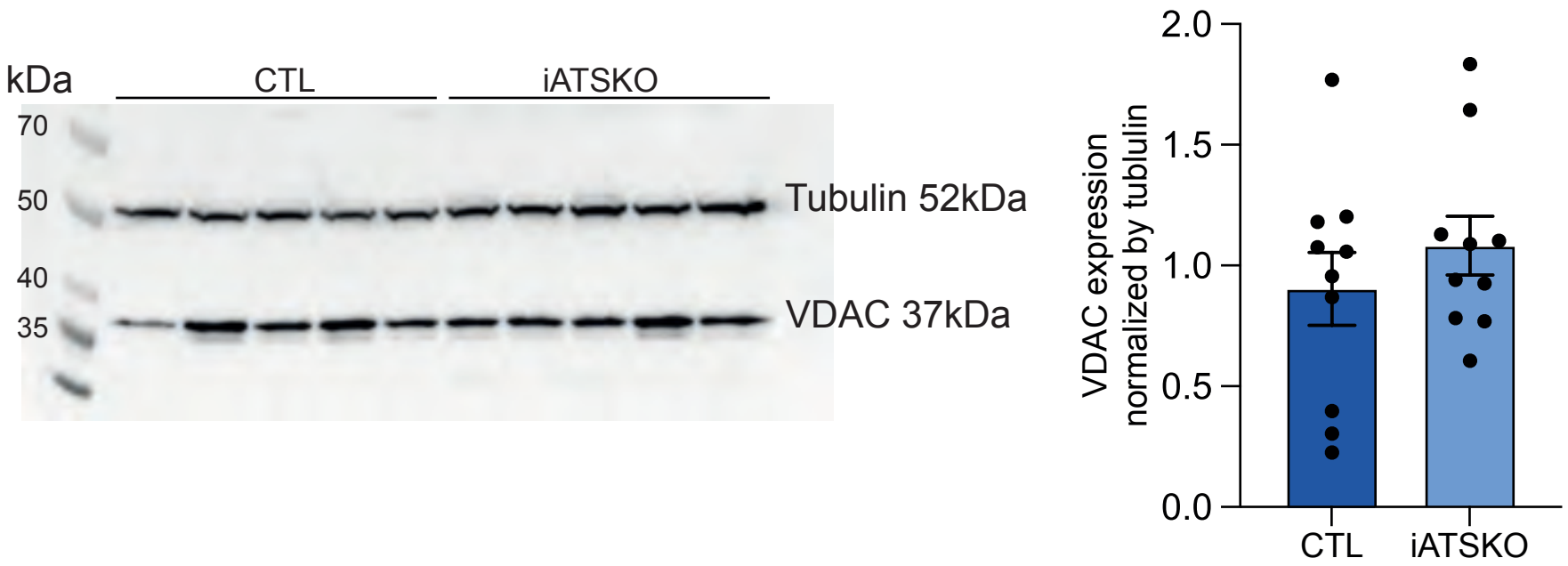

Appendix Figure S5

Representative total protein blots of phospho-PDH (P-PDH) and total PDH in siNTC and siBSCL2 adipocytes ± Linker-ER-Mi are shown (A, left). Representative total protein blots of P-PDH and PDH in inguinal adipose tissue lysates from control and iATSKO mice are shown (H, right). Representative immunoblots of VDAC and tubulin are shown and quantified (B). Bars represent mean ± SEM; n = 9 mice per genotype. Mann–Whitney test.

Appendix figure S6

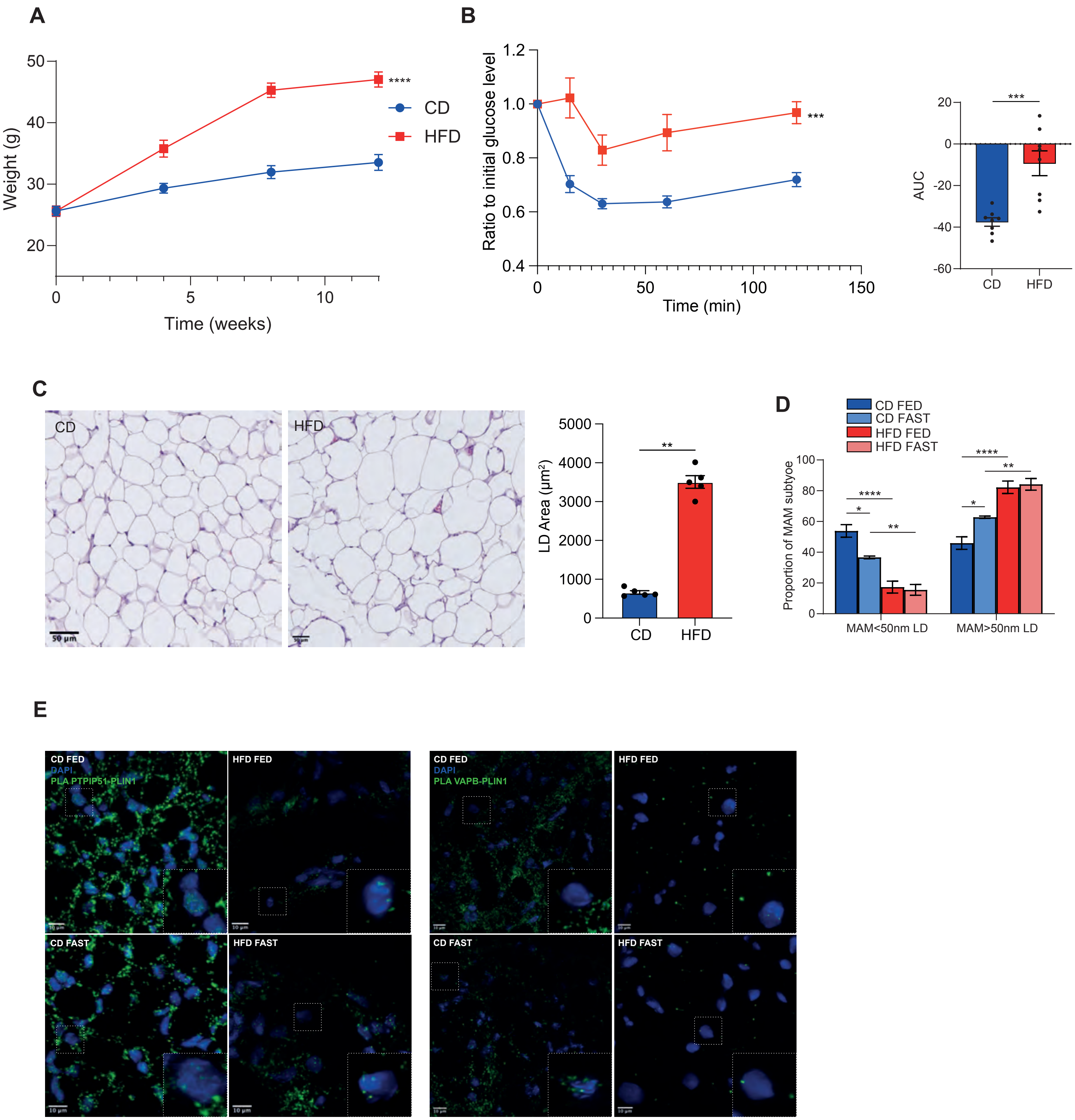

Appendix Figure S6

Mice were fed either a chow diet or a high-fat diet for 3 months. Body weight was monitored throughout the experiment (A), and insulin sensitivity was assessed after 12 weeks of diet and normalized to initial glucose levels (B). Gonadal adipose tissues from chow- or high-fat diet-fed mice were collected in either the fed state or after an 18 h fast. Representative histological images of gonadal adipose tissue and quantification of LD area using the Adiposoft Fiji plugin are shown (D). Bars represent mean ± SEM; n = 5 mice per group. *p < 0.05, ***p < 0.001, ****p < 0.0001; Mann–Whitney test. Transmission electron microscopy was used to quantify the proportion of MAMs located <50 nm or >50 nm from LDs. Bars represent mean ± SEM; n = 3 mice per group, with 5 cells analyzed per mouse. *p < 0.05, ***p < 0.001, ****p < 0.0001; two-way ANOVA. Representative PLA images of PTPIP51-PLIN1 and VAPB-PLIN1 are shown (E).

Appendix figure S7

A

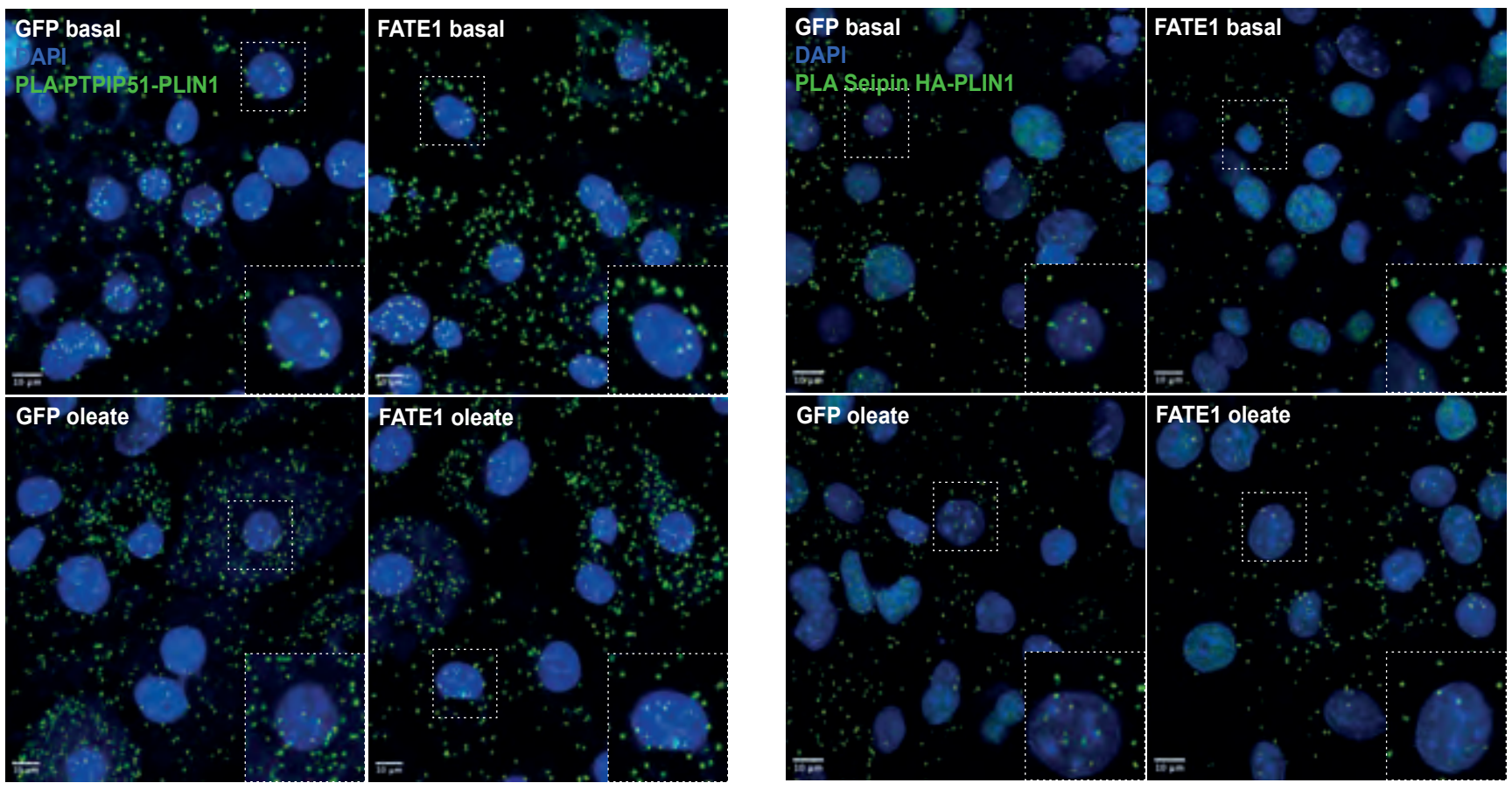

B

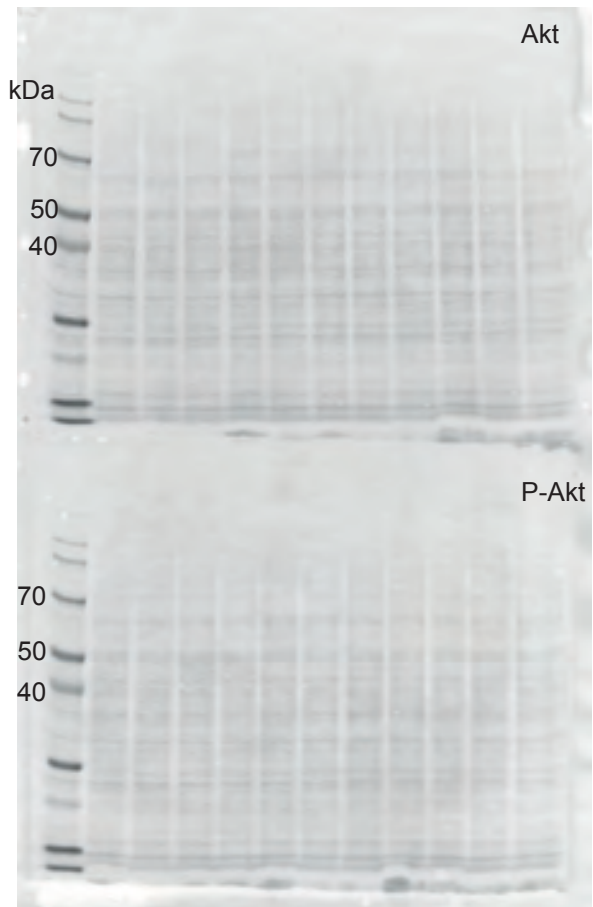

C

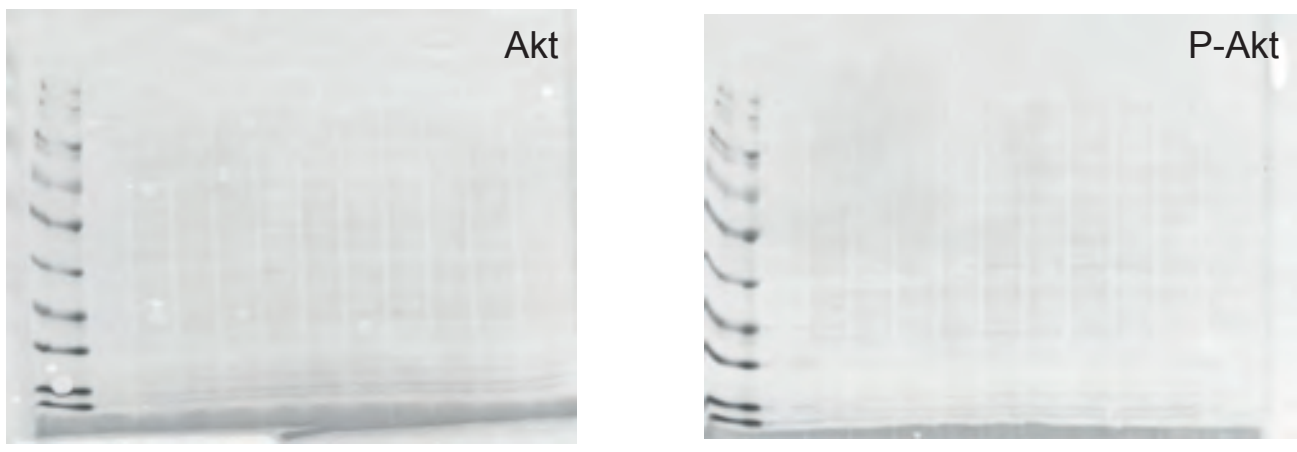

Appendix Figure S7
3T3-L1 adipocytes, after 7 days of differentiation were infected with adenovirus expressing GFP or FATE1. Representative images of PLA PTPIP51-PLIN1, and Seipin HA-PLIN1 in basal (6H, 5mM glucose) and oleate treated condition (6h, 5mM glucose, 700µM oleate) (A). Total protein blot of Akt and P-Akt is shown (B). CTL and KO PTPIP51 3T3-L1 adipocytes were starved and then stimulated by insulin. Total protein loading blots for Akt and phospho-Akt (P-Akt) (C).

Appendix figure S8

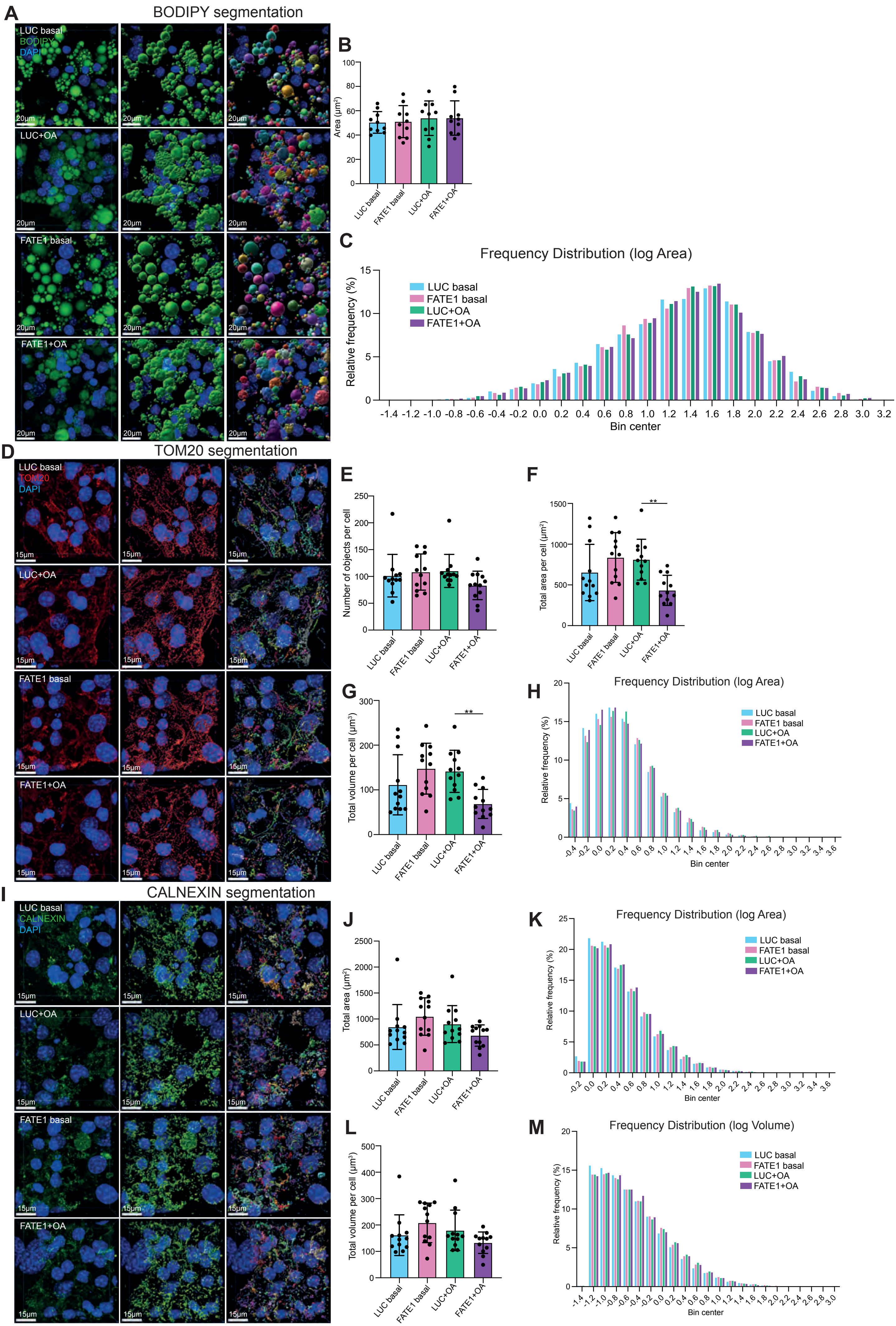

Appendix Figure S8

3T3-L1 adipocytes, after 7 days of differentiation were infected with adenovirus expressing GFP or FATE1. Cells were stained with BODIPY to visualize LDs (A), anti-Tom20 to label mitochondria (E), and anti-calnexin to label the ER (F). Quantitative analyses were performed using Imaris software. For each condition, experiments were performed in duplicate wells, and a total of 15 images, representing approximately 150 cells, were analyzed per condition.

Appendix figure S9

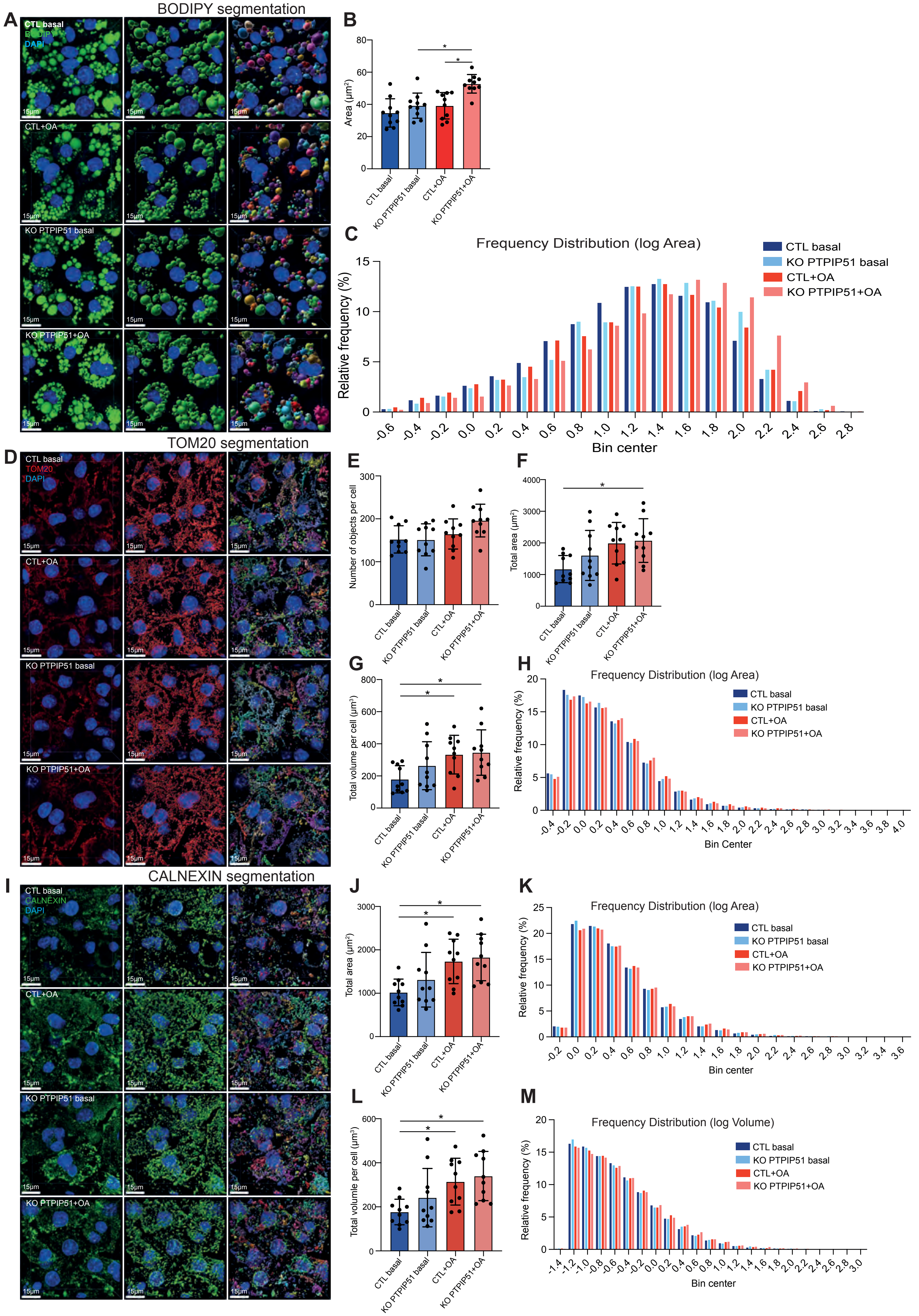

Appendix Figure S9

CTL and KO PTPIP51 3T3-L1 adipocytes, were incubated in low-glucose media +/- oleic acid for 6 hours. Cells were stained with BODIPY to visualize LDs (A), anti-Tom20 to label mitochondria (E), and anti-calnexin to label the ER (F). Quantitative analyses were performed using Imaris software. For each condition, experiments were performed in duplicate wells, and a total of 15 images, representing approximately 150 cells, were analyzed per condition.
