# Supplementary material for: Tripartite ER-mitochondria-lipid droplets contact sites control adipocyte metabolic flexibility
Source: EMBO J. 2026 Jul 22;45(17):6021–53. doi: 10.1038/s44318-026-00876-z (PMC13534470; doi:10.1038/s44318-026-00876-z)
Supplement: Supplementary file 3 [file 44318_2026_876_MOESM2_ESM.pdf]

THE EMBO JOURNAL

Tripartite ER-Mitochondria-Lipid Droplets contact sites control adipocyte metabolic flexibility

Marie Palard, Gilliane Chadeuf, Maxime Carpentier, Clotilde Renaud, Samuel Frey, Mikael Croyal, Rola Shaaban, Samy Hadjadj, Chloe Cloteau, Naima El Khallouki, Perrine Bomme, Soazig Le Lay, Manuel Majrouh, Perrine Paul-Gilloteaux, Alexandre Humbert, Christine Durand, Nicolas Bertocchini, Laurence Dubreil, Yoann Combot, Antria Antreou, Cédric Le May, Simon Ducheix, Bertrand Cariou, Francesca Giordano, Jennifer Rieusset, Abdou Thiam, and Xavier Prieur

Corresponding author(s): Xavier Prieur (xavier.prieur@univ-nantes.fr) , Jennifer Rieusset (jennifer.rieusset@univ-lyon1.fr), Abdou Thiam (thiam@phys.ens.fr)

Review Timeline:

Submission Date:	12th Dec 25
Editorial Decision:	2nd Feb 26
Revision Received:	27th May 26
Editorial Decision:	1st Jul 26
Revision Received:	3rd Jul 26
Accepted:	9th Jul 26

Editor: William Teale

Transaction Report:

(Note: With the exception of the correction of typographical or spelling errors that could be a source of ambiguity, letters and reports are not edited. Depending on transfer agreements, referee reports obtained elsewhere may or may not be included in this compilation. Referee reports are anonymous unless the Referee chooses to sign their reports.)

1st Editorial Decision **2nd Feb 2026**

Dear Xavier,

Thank you again for the submission of your manuscript entitled "Tripartite ER-Mitochondria-Lipid Droplets contact sites control adipocyte metabolic flexibility" and for your patience during the review process. We have now received reports from three referees, which I copy below.

As you can see from their comments, all thought that your manuscript could represent a useful and timely development to our understanding of the relevance of mitochondria-ER-lipid droplet contacts to lipid loading in adipocytes. That said, all of them point out weaknesses in how your data are presented, analysed and (in some places) verified. This will require your attention before your manuscript can be published in The EMBO Journal.

Based on the overall interest expressed in the reports, however, I would like to invite you to address the comments of all referees in a revised version of the manuscript. I should add that it is The EMBO Journal policy to allow only a single major round of revision and that it is therefore important to resolve the main concerns at this stage. I believe the concerns of the referees are reasonable and addressable, but please contact me (I am always available for a Zoom discussion) if you have any questions, need further input on the referee comments or if you anticipate any problems in addressing any of their points. Please, follow the instructions below when preparing your manuscript for resubmission.

I would also like to point out that as a matter of policy, competing manuscripts published during this period will not be taken into consideration in our assessment of the novelty presented by your study ("scooping" protection). We have extended this 'scooping protection policy' beyond the usual 3 month revision timeline to cover the period required for a full revision to address the essential experimental issues. Please contact me if you see a paper with related content published elsewhere to discuss the appropriate course of action.

When preparing your letter of response to the referees' comments, please bear in mind that this will form part of the Review Process File, and will therefore be available online to the community. For more details on our Transparent Editorial Process, please visit our website: https://link.springer.com/journal/44318/submission-guidelines#cms-Revised-submissions

Again, please contact me at any time during revision if you need any help or have further questions.

Thank you very much again for the opportunity to consider your work for publication. I look forward to your revision.

Best regards,

William

William Teale, Ph.D.
Editor
The EMBO Journal

When submitting your revised manuscript, please carefully review the instructions below and include the following items:

1) a .docx formatted version of the manuscript text (including legends for main figures, EV figures and tables). Please make sure that the changes are highlighted to be clearly visible.

2) individual production quality figure files as .eps, .tif, .jpg (one file per figure).

3) a .docx formatted letter INCLUDING the reviewers' reports and your detailed point-by-point response to their comments. As part of the EMBO Press transparent editorial process, the point-by-point response is part of the Review Process File (RPF), which will be published alongside your paper.

4) a complete author checklist, which you can download from our author guidelines (https://link.springer.com/journal/44318/submission-guidelines#cms-Revised-submissions). Please insert information in the checklist that is also reflected in the manuscript. The completed author checklist will also be part of the RPF.

5) Please note that all corresponding authors are required to supply an ORCID ID for their name upon submission of a revised manuscript.

6) We require a 'Data Availability' section after the Materials and Methods. Before submitting your revision, primary datasets produced in this study need to be deposited in an appropriate public database, and the accession numbers and database listed

under 'Data Availability'. Please remember to provide a reviewer password if the datasets are not yet public (see https://link.springer.com/partners/embo-press/editorial-policies#Data%20deposition). If no data deposition in external databases is needed for this paper, please then state in this section: This study includes no data deposited in external repositories. Note that the Data Availability Section is restricted to new primary data that are part of this study.

Note - All links should resolve to a page where the data can be accessed.

7) When assembling figures, please refer to our figure preparation guideline in order to ensure proper formatting and readability in print as well as on screen:
https://link.springer.com/journal/44318/submission-guidelines#cms-Figure-and-data-presentation

Please remember: Digital image enhancement is acceptable practice, as long as it accurately represents the original data and conforms to community standards. If a figure has been subjected to significant electronic manipulation, this must be noted in the figure legend or in the 'Materials and Methods' section. The editors reserve the right to request original versions of figures and the original images that were used to assemble the figure.

8) For data quantification: please specify the name of the statistical test used to generate error bars and P values, the number (n) of independent experiments (specify technical or biological replicates) underlying each data point and the test used to calculate p-values in each figure legend. The figure legends should contain a basic description of n, P and the test applied. Graphs must include a description of the bars and the error bars (s.d., s.e.m.).

9) We would also encourage you to include the source data for figure panels that show essential data. Numerical data can be provided as individual .xls or .csv files (including a tab describing the data). For 'blots' or microscopy, uncropped images should be submitted (using a zip archive or a single pdf per main figure if multiple images need to be supplied for one panel). Additional information on source data and instruction on how to label the files are available at
https://link.springer.com/journal/44318/submission-guidelines#cms-Source-data

10) We replaced Supplementary Information with Expanded View (EV) Figures and Tables that are collapsible/expandable online (see examples in https://www.embopress.org/doi/10.15252/embj.201695874). A maximum of 5 EV Figures can be typeset. EV Figures should be cited as 'Figure EV1, Figure EV2" etc. in the text and their respective legends should be included in the main text after the legends of regular figures.

- For the figures that you do NOT wish to display as Expanded View figures, they should be bundled together with their legends in a single PDF file called *Appendix*, which should start with a short Table of Content. Appendix figures should be referred to in the main text as: "Appendix Figure S1, Appendix Figure S2" etc. See detailed instructions regarding expanded view here: < https://link.springer.com/journal/44318/submission-guidelines#cms-Expanded-View-data >.

- Additional Tables/Datasets should be labelled and referred to as Table EV1, Dataset EV1, etc. Legends have to be provided in a separate tab in case of .xls files. Alternatively, the legend can be supplied as a separate text file (README) and zipped together with the Table/Dataset file.

11) At EMBO Press we ask authors to provide source data for the main manuscript figures. Our source data coordinator will contact you to discuss which figure panels we would need source data for and will also provide you with helpful tips on how to upload and organize the files.

12) Our journal encourages inclusion of *data citations in the reference list* to directly cite datasets that were re-used and obtained from public databases. Data citations in the article text are distinct from normal bibliographical citations and should directly link to the database records from which the data can be accessed. In the main text, data citations are formatted as follows: "Data ref: Smith et al, 2001" or "Data ref: NCBI Sequence Read Archive PRJNA342805, 2017". In the Reference list, data citations must be labeled with "[DATASET]". A data reference must provide the database name, accession number/identifiers and a resolvable link to the landing page from which the data can be accessed at the end of the reference. Further instructions are available at < https://link.springer.com/journal/44318/submission-guidelines#cms-Reference-guidelines >.

13) In order to increase the reproducibility and reach of your work, The EMBO Journal includes a table of reagents that were used in the study. Please provide this along with your revisions.

Instructions for preparing your revised manuscript:

Read our guidance for manuscript revisions and related editorial policies: https://link.springer.com/journal/44318/submission-guidelines#cms-Revised-submissions

Please make sure you upload a letter of response to the referees' comments together with the revised manuscript.

Please also check that the title and abstract of the manuscript are brief, yet explicit, even to non-specialists.

When assembling figures, please refer to our figure preparation guideline in order to ensure proper formatting and readability in print as well as on screen:
https://media.springernature.com/original/springer-cms/rest/v1/content/27825798/data/v1

At EMBO Press we ask authors to provide source data for the main manuscript figures. You will receive a separate email with instructions for providing source data with your revised manuscript, including how to upload and organize the files.

IMPORTANT: When you send the revision we will require
- a point-by-point response to the referees' comments, with a detailed description of the changes made (as a word file).
- a word file of the manuscript text.
- individual production quality figure files (one file per figure)
- a complete author checklist
- Expanded View files (replacing Supplementary Information)
- a Reagents and Tools Table as part of the Methods section

Please remember: Digital image enhancement is acceptable practice, as long as it accurately represents the original data and conforms to community standards. If a figure has been subjected to significant electronic manipulation, this must be noted in the figure legend or in the 'Materials and Methods' section. The editors reserve the right to request original versions of figures and the original images that were used to assemble the figure.

We realize that it is difficult to revise to a specific deadline. In the interest of protecting the conceptual advance provided by the work, we recommend a revision within 3 months (3rd May 2026). Please discuss the revision progress ahead of this time with the editor if you require more time to complete the revisions. Use the link below to submit your revision:

https://emboj.msubmit.net/cgi-bin/main.plex

Referee #1:

Palard et al., characterize changes in membrane contact sites in adipocytes under different metabolic conditions. They first use an in vivo model to show that loss of the lipid droplet anabolic protein seipin dysregulates ER-mitochondria, ER-LD, and LD-mitochondria contact sites. Furthermore, the loss of seipin promotes metabolic inflexibility, a function that is rescued by expression of a synthetic ER-mitochondria tether. The authors further pinpoint a defect in the transfer of triglycerides from the ER to lipid droplets with the loss of seipin. Broadening their analysis, Palard et al. describe how these contacts change with adipocyte differentiation and in models of obesity. While the author's findings provide important mechanistic details regarding the regulation of ER, mitochondria, and LD contact sites in different metabolic states, this work is limited by numerous technical issues, making the interpretation of their results difficult.
Major Issues:
1. A major issue with this manuscript is how the data is represented. It is often unclear what is being measured and what variation exists within that population.
a. Many different metrics for measuring aspects of MCSs are used throughout this study; however, the authors rarely take the time to explore how these analyses are different and often one analysis will be used for one experiment and a different analysis for a second. For example, the length of a contact and the number of contacts are not the same. Furthermore, it is rarely explained in the main text, methods, or figure legend what is being measured. Take figure 2 for example. In 2B "% contact" is measured, which, according to the figure legend, is "analysis of quantified MCS". It is unclear what exactly this is measuring, which is particularly important when the MAM results for figures 2B and 2C do not agree. Additionally, several different ways of normalizing the data are used, such as total contacts versus total contacts/# of mitochondria. Given that seipin is an LD biogenesis protein, it may be prudent to also test normalization of contacts with LDs to the number of LDs. While LD area is measured in iATSKO, it does not appear to be measured following seipin KD.
b. Throughout their manuscript the authors represent nearly all of their data as bar graphs with no accompanying swarm plot to indicate the data distribution. This is particularly important when relatively small n values are used. For example, it appears that only 2 mice are used with 5 cells per mouse in the first experiments.
c. PLA is used consistently throughout the manuscript but representative images are rarely, if ever, shown.
d. In several places in the manuscript the # of contacts is measured (e.g. Fig. 2C-E). However, it is unclear how this measurement was performed. Is this PLA? If not then it should be stated in the methods how this was performed. For a paper that is mostly based on microscopy, an explanation of how image analyses were performed is severely lacking.
2. For the experiment represented in figures 3B, 3C and supplemental 3B, parallel conditions with siNTC should be employed.
3. The authors consistently use PLA throughout the manuscript as a method of measuring MCSs, but fail to recognize that this method only measures specific interactions and not bulk MCSs. In the discussion, the authors explore how MAM appears to

have a different function when it is interacting with LDs. Similarly, different organelle-organelle interactions serve different functions depending on the suite of MCS proteins present. For example, as the authors are likely aware, IP3R-VDAC specifically functions in calcium transfer. In particular, it is strange that the authors chose to use PTPIP51 - PLIN1 as a marker of mito-LD contacts, given that this is not a well-established contact, while MFN2 - PLIN1 is.
4. Membrane contact sites are not static but rather dynamic structures that should persist over time. While I appreciate that to achieve the appropriate resolution fixation is preferred, live imaging should be used as an orthogonal experiment to prove that the contacts that the authors are quantifying are actually stable MCSs. This is particularly prudent given that organelle shape and number changes with the experimental setting tested, which could bias a fixed cell analysis towards more false positives as the cellular environment becomes more or less crowded.
5. I appreciate how PDH phosphorylation is linked with mitochondrial calcium levels, so the experiments in Fig 3F-I provide insights into how much pyruvate is making it into the TCA cycle. However, this work and the experiment depicted in Fig 3J only allow you to draw indirect conclusions about fatty acid metabolism. Please address this in the text or complete further experiments to better measure changes in fatty acid metabolism.
Minor Issues:
1. The authors indicate that a proteomic analysis is performed (Fig. S1H) but no methods or further details are provided for this experiment.
2. The authors should quantify how the abundance of PDM and CM (independent of their interaction with the ER) changes with seipin depletion. Also, I encourage the authors to use the term PDM throughout the manuscript.
3. Validation of the seipin knockout and BSCL2 knockdown is missing.
4. Figure 1I indicates what each channel represents.
5. In the first section of the results, the authors write "...MAM-CM and MAM-LD levels..." What do the authors mean by "levels" here?
6. Figure 2N, appears that the linker conditions are missing from the line graph as they are only present in the accompanying bar graph.
7. In the second section of the results, the authors write "... both mitochondria and ER appear loosely organized..." A further description of what the authors mean by "loosely organized" would be helpful.
8. The indicators of statistical significance in Fig 4A are very confusing. Please edit this graph to be more intuitive.
9. Fig 4C - Did you perform statistical analysis on these results? If so, please indicate whether these changes in organelle interaction percentages are significant.
10. Fig 4J-L: I appreciate the dots indicating the individual data points, but there is no contrast between the data points and the bar colors. Please change one or both of these so you can see the data points that overlap with the bar.
11. Supplementary Fig 4F is not cited in the text.
12. Fig 5J - The y-axis is just %. Percent of what? Please indicate on the figure.
13. I'm not convinced by the statement you make about Figs 6G-I ("... and this decrease is mainly explained by the decreases of MAMs-LD as MAMs-CM are not significantly reduced.") It feels like your n may need to increase because there is a lot of variation in the dataset. More replicates should better reveal what trends there are with more confidence.
14. The asterisks on Figure 4Q are misaligned.
15. The authors should acknowledge that several other studies (albeit in different cell types) have previously shown that loss of PTPIP51 alters respiration and calcium flux.
16. As far as I can tell, supplemental Figure 2 is never referenced.
17. Supplemental figures 4 and 5 do not contain any legends for the bar graphs.
18. A summary figure would make interpretation of the results clearer, particularly given that the author's manuscript touches on many different aspects of adipocyte metabolism.
19. Many graphs are lacking titles and have vague y-axis labels (e.g., "%", "Ratio"). Providing a simple title would facilitate the interpretation of the results.

Referee #2:

The authors address an important subject: the mechanism underlying Seipin deficiency effects on membrane contacts and lipid handling. Authors show that MCs, lipid handling, and metabolic flexibility are significantly altered in adipose tissues upon Seipin depletion. These alterations were associated with decreases in both mitochondrial respiration and calcium flux, and could be rescued by reinforcing ER-Mitochondria contacts.

Overall, the manuscript is well written and has a sound conceptualization.

However, it consistently lacked robust, well-defined morphometric quantification methods for membrane contact sites, proper normalization, and appropriate statistics.

The authors noted that Seipin-KO in mice results in an 80% reduction in adipose mass, necessitating the use of an earlier time point post-Seipin-KO. However, Authors fail to provide sufficient evidence that this KO phenotype has not yet manifested as a reduction in LD area at their chosen time point. Ideally, mitochondria and LDs should be stained and confocal microscopy used to quantify their gross morphology.

Throughout the manuscript, the authors relied heavily on TEM imaging, severely limiting the study's statistical power. For example, only 10 cells per group were analyzed in the TEM images shown in Figure 1. Robust morphometric methods, complemented with additional vetted techniques, are typically required when TEM is employed to study membrane contact site biology. Proximity ligation assay is a valuable complement to TEM results; however, it must be properly normalized to account for morphological alterations caused by Seipin-KO.
C
Confusing nomenclature is used throughout the manuscript; for example, the term "MAM-LD" to describe tripartite contacts is ambiguous and could be interpreted as either ER-Mito-LD or Mito-ER-LD contacts. This is not a generally accepted terminology and has been used only once in published literature. It is unclear what organelle contact length means - is it the distance between the apposed organelles, or the length of the regions closely apposed with each other? If the latter is what authors mean, then the reported numbers are well outside the range generally accepted by the field as a bona fide contact site. Regardless, this metric needs to be properly normalized to organelle perimeter or area and presented as percentages.

Lastly, statistical tests were often used inappropriately. The Mann-Whitney test was misused in several figures that compared more than 2 experimental conditions. The standard practice when using the Mann-Whitney test is to display the data as violin plots with individual data points and ranked quartiles. Comparisons across more than 2 conditions should use one- or two-way ANOVAs, as appropriate. Furthermore, the use of SEM rather than SD is not justified, as the authors reported experimental replicates with N < or = 3.

Minor Comments
All images need scale bars and channel labels. All Western blots need tick marks of the closest molecular weight marker. Western blot quantifications need to be normalized to mitochondrial mass (TOM20/VDAC) or a housekeeping protein (Actin), and/or the total protein stain needs to be shown. Mitochondrial calcium imaging showing ratios need to be normalized to biomass. The authors need to always state the total number of mice and cells analyzed for each experiment. Nomenclature should be used consistently throughout the manuscript for better clarity. The y-axis should always have easily interpretable titles that clearly state what was measured.

Specific comments

Figure1
The comments below are relevant to all other figures that use TEM and PLA to quantify contact sites.

For EM micrographs, it is currently unclear how the number of MAMs, MAM-LD, and MAM-CM was scored. Contact site distances can vary (generally considered <30nm) within a given contact site, so the distribution of distances between organelles at the contact needs to be quantified and shown. Furthermore, contact site lengths are usually expressed as a percentage of the organelle coverage. Given that the Seipin-KO condition alters mitochondrial morphology, the authors should normalize contact site lengths to mitochondrial perimeter or area. Presenting contact site quantification results as total counts can mask the differences in organelle abundance between the control and KO; authors should compare percentages rather than counts. It is often unclear how many cells were analyzed for the quantifications. Reliance on TEM imaging to quantify mitochondrial and LD morphology limits analyses to a single plane. Thus, statistically robust quantification is nearly unattainable, as TEM images do not fully capture a cell's gross morphology.

The authors did a great job of complementing TEM results by using Proximity ligation, a well-established quantitative technique. However, the total number of PLA dots/puncta needs to be properly normalized to the organelle in question. Since Total MAMs were significantly different between Control and KO, #MAM-LD and MAM-CM should be normalized to total #MAM (Figure1G).

All images need scale bars and channel labels. For example, it is not clear which channel is cyan in Figure1F, or which channels are blue or red in Figure 1I.

The authors say that "MAM was not significantly different between genotypes (Figure 1G)". This should say "MAM-CM was not significantly different between genotypes".

The nomenclature "MAM-LD and MAM-CM" is quite ambiguous and can be interpreted as ER-Mito-LD or Mito-ER-LD contacts. For better clarity, the authors should consider changing it to "MAMs-LD distance" < 380nm and > 380nm. The 380nm classification has prior justification in a previous publication, but the authors still need to clearly state this rationale in the text or legend.

The Y axes are often labeled too vaguely and can be better clarified by including a short descriptive term that defines what was actually measured. For example, "MAM-LD" can be further defined as "IP3R-PTP51 PLA punctae >380nm from LD".

Consider using CTL and iATSKO consistently throughout the manuscript.

Lipidomics data is pixelated and impossible to see. Lipidomics shows a marked decrease in TG and DG, but, surprisingly, this is not concomitant with reductions in LD area as quantified by TEM. This may demonstrate the limitations of using TEM for quantifying gross differences in LDs and mitochondrial morphology. Authors should use conventional techniques to provide statistically robust quantification of LD and mitochondrial morphology.

Figure 2
In 2B-F, it is unclear how the percentage contact and the number of contacts were calculated.
In 2I-M, for spatial proximity using SIM, it is unclear how contact sites were quantified. Furthermore, SIM-based methods for measuring proximity are constrained by spatial resolution (typically around ~300nm), which is outside the limits of what the field generally accepts as bona fide contact sites. In other words, spatial proximity analysis is not a direct measure of membrane contact sites. Furthermore, the authors do not state how many cells were analyzed by this technique. Authors use an ER-Mitochondrial linker but do not demonstrate or quantify ER-Mitochondria contacts as proof of concept that the linker increases ER-mitochondria proximity. Instead, Authors quantified ER-LD-Mitochondria proximity, MAMs, MDM-LD, and MAM-CM, and it is currently unclear how this was done. Importantly, the authors used a 380nm threshold to validate membrane contacts, a threshold specific to the proximity ligation technique, which was not used here. Ideally, proximity ligation assays should have been used to complement TEM results here.
In Figure 2N, the authors should include the siNTC-GFP and SiNTC+Linker traces to clearly demonstrate rescue. Also, the linker failed to improve recovery in the NTC background, and this should at least be mentioned in the text, along with the authors' explanation for this observation.

Figure 3
In Figures 3A-C, it is unclear how fluorescence ratios and % recovery were calculated. This needs to be restated regardless of previous figures. In 3D-E, it is unclear what ratios are calculated for spatial analyses. Western blots need a tick mark for the molecular weight of the closest protein ladder marker. In addition, p-PDH/PDH ratios should be further normalized to a mitochondrial mass marker to account for differences in total mitochondria. Citrate enrichment needs to be properly normalized to biomass.

Figure4 and 5
Better clarification is needed regarding how # of contact sites was determined by TEM. Since nutrient perturbations most likely alter mitochondrial and LD morphologies, PLA dots should be normalized to mitochondrial or LD perimeter (2D segmentation) or area (3D segmentation), as appropriate. Contact site nomenclature should be consistent (e.g., mito-LD, not mito/LD). It is unclear how tripartite contacts were quantified. Images need scale bars. In Figure 5, the total contact length was measured, but the authors need to clarify how it was normalized.

Figures 6 and 7
PLA dots should be normalized to mitochondrial or LD perimeter (2D segmentation) or area (3D segmentation), as appropriate. Western blots need to be normalized to the total protein loaded. Glycerol concentration needs to be normalized to biomass.

Supplemental
S1: It needs to be stated how the LD area was normalized.

S2: Images need scale bars, channel labels, and boxes indicating the ROIs that were magnified.

S3: MCU knockdown needs to be quantitatively validated at the protein level.

S4: Glycemia levels need to be normalized to weight. Scale bars needed for images.

S5: Western blot and Oil red staining need quantification. Mitochondrial calcium and OCR measurements need to be normalized to biomass.

Referee #3:

The manuscript by Pallard et al. "Tripartite ER mitochondria lipid droplet contact sites control adipocyte metabolic flexibility" represents a signifant conceptual advance. Contact sites between several organelles are known to be important for cell function but their identity as dynamic hubs in the control of metabolic activity of various cells such as adipocytes remains poorly understood. Pallard et al demonstrate in that adipocyte function in fat storage depends on the interplay of at least three organelles including the ER, mitochondria and lipid droplets.

Overall this is an extremely timely study, rich in novelty and conceptually creative. The paper starts with the investigation of seipin as an important factor in lipid droplet maintenance within adipose tissue. The authors introduce a new experimental

paradigm exploiting the inducible knock out of seipin in mouse.
The authors investigate the seipin KO mice already 15 days after induction of the gene knockout.
Loss of seipin function causes lipodystrophy a condition marked by the absence of functional white adipose tissue but the mechanisms that lead to the defect and the cell biological function of seipin in adipose tissue is not yet clear. With the new approach the authors shed light on the mechanisms by which seipin forms a contact site between the ER and mitochondria and clarify how calcium flux through this interface influences lipid flux to the LDs.

The strength of the paper is the creative interpretation of the data and the multimodal approach. Contact site formation is modulated and assessed in multiple ways. The EM is impressive and extensively quantified, combined with fluorescence microscopy and metabolic assays. While this is the strength of the work it sometimes turns into a problem because the early and later figures seem disconnected in content and approach. This needs to be more consistent before publication.

The major conclusion is interesting. Seipin dependent contact sites between mitochondria and the ER control calcium accumulation in the mitochondria. Although previously reported by the Prieux lab its identification as a metabolic switch is novel: The contact site switches ON pyruvate dehydrogenase as well as enzymes in the Krebs cycle to synthesise citrate as a precursor for fatty acid and triglyceride biosynthesis.

In obesity and under high glucose conditions the contact sites break similar as in seipin depleted adipose tissue which is of considerable conceptual novelty. Despite the lack of a precise molecular mechanism the physiology is compelling. The paper is therefore a good contender for EMBO journal. The quality of figures and the coherence across figures may be improved as a condition for publication.

Specific Comments

The conclusion in the first part of the paper depends on the fluorescence recovery assay using triglycerides labelled with fluorescent fatty acids. Loss of seipin slightly attenuates the recovery after bleaching (100 sec long experiments). It is unclear how the authors explain this effect mechanistically. Is this working through calcium? If yes, why would the effect be so fast (100 sec recovery). If not how would depletion of seipin stop lipid exchange with the ER? Recent work on seipin suggests that inactivation of seipin through adipogenin activates lipid storage in adipocytes. This part of the paper is conceptually and mechanistically not sound and needs improvement. For example a simple way to test whether triglyceride synthesis is required (calcium dependence) is to add DGAT inhibitors and see whether the recovery does not work. This would support a defect in de novo synthesis of TGs. If the TGs just move to the LDs and this is blocked without seipin the authors need to explain where the TGs are stored before movement.

A next point which needs explanation is which subtype of the various MAM-LD contact sites is important. This reviewer is not clear about which contact site is under the control of seipin. The confusion mainly arises because the paper moves from seipin to PTPIP51 and then to FATE. Neither of these proteins acts together with seipin. The authors use them as tools to modulate the contact sites but the conclusions regarding seipin function are not clearly set out.

The contact site markers for the proximity labelling require validation. Is the localisation of organelle markers suitable for the assay. Total MAM markers IP3R-VADAC are validated in the literature but this reviewer is not aware of an interaction between VAP proteins and PLIN1 as well as PTPIP51 and PLIN1. How were these partners validated and are they interacting or simply marking the organelles that are in close apposition?

Minor comments

The figure labelling should be revised and double checked.

Fig. 1 H: plasmic membrane - plasma membrane

The title in Fig. 2 is confusing: Please correct the language.

Fig. 3: calcium dependent.

Fig. 4: it is unclear what J0, J2...J8 reflect in Fig. 4. This reviewer suspects that this is an abbreviation for the word jour in French meaning day. The figure should be labelled in Englisn

Fig. 7: MAM re-inforcement in MCS altered cellular model....- this is incomprehensible. Please rewrite.

Referee #1:

Palard et al., characterize changes in membrane contact sites in adipocytes under different metabolic conditions. They first use an in vivo model to show that loss of the lipid droplet anabolic protein Seipin dysregulates ER-mitochondria, ER-LD, and LD-mitochondria contact sites. Furthermore, the loss of Seipin promotes metabolic inflexibility, a function that is rescued by expression of a synthetic ER-mitochondria tether. The authors further pinpoint a defect in the transfer of triglycerides from the ER to lipid droplets with the loss of Seipin. Broadening their analysis, Palard et al. describe how these contacts change with adipocyte differentiation and in models of obesity. While the author's findings provide important mechanistic details regarding the regulation of ER, mitochondria, and LD contact sites in different metabolic states, this work is limited by numerous technical issues, making the interpretation of their results difficult.

Major Issues:

1. A major issue with this manuscript is how the data is represented. It is often unclear what is being measured and what variation exists within that population.

a. Many different metrics for measuring aspects of MCSs are used throughout this study; however, the authors rarely take the time to explore how these analyses are different and often one analysis will be used for one experiment and a different analysis for a second. For example, the length of a contact and the number of contacts are not the same. Furthermore, it is rarely explained in the main text, methods, or figure legend what is being measured. Take figure 2 for example. In 2B "% contact" is measured, which, according to the figure legend, is "analysis of quantified MCS". It is unclear what exactly this is measuring, which is particularly important when the MAM results for figures 2B and 2C do not agree. Additionally, several different ways of normalizing the data are used, such as total contacts versus total contacts/# of mitochondria. Given that Seipin is an LD biogenesis protein, it may be prudent to also test normalization of contacts with LDs to the number of LDs. While LD area is measured in iATSKO, it does not appear to be measured following Seipin KD.

We thank the reviewer for this important comment.

Regarding clarification and standardization of MCS quantification: We acknowledge that, in the initial version of the manuscript, the terminology used to describe contact site parameters was not sufficiently standardized and that the presentation of the data lacked clarity regarding what was being measured. We have now revised the manuscript to clarify these points throughout the text, figure legends, and Methods section. In particular, we added a dedicated paragraph at the beginning of the Results section (page 4), together with a schematic representation of the quantified parameters (Appendix Figure S1) and a dedicated Methods section (page 18).
« Throughout this manuscript, we quantified organelle contact sites using TEM by analyzing ER–mitochondria contacts (i.e., MAMs), ER–lipid droplet (ER–LD) contacts, and mitochondria–LD (Mi–LD) contacts. Two parameters were measured: (1) the contact number, defined as the occurrence of contacts where the distance between organelles is <50 nm, and (2) contact surface, defined as the length of the contact normalized to the mitochondrial perimeter for MAMs and Mi–LD contacts, or to the LD perimeter for ER–LD contacts (as indicated in the axes and figure legends; see Appendix Fig S1 and Methods section). Because this parameter is normalized to the mitochondrial or LD perimeter, it does not simply reflect changes in organelle size or morphology, but rather specifically reports the structural properties of the contact sites. *» (page 4).*

Importantly, contact number and contact surface represent distinct structural features of membrane contact sites and are therefore not expected to vary identically under all conditions. We now explicitly clarify this point in the revised manuscript.

« MAM surface (Fig 2E) and MAM number (Fig EV2C) were decreased in Seipin knockdown cells under basal conditions. Unexpectedly, OA treatment induced a slight increase in MAM number compared with the basal state, but only in Seipin-deficient cells". (page 6).

In the discussion we further comment:

« Interestingly, under OA loading in vitro, Seipin deficiency reduces MAM surface area while increasing MAM number, suggesting a redistribution toward smaller but more numerous contacts » (page 11).

Regarding lipid droplet (LD) properties in Seipin-deficient cells: We agree that this is an important point given the established role of Seipin in LD biology. However, our study focuses on acute—or early—time points following Seipin deletion, both in vivo and in vitro, in order to minimize secondary effects associated with severe alterations in LD and adipocyte function. Accordingly, 15 days after tamoxifen injection in iATSKO mice, adipose tissue loss remains moderate (~20%), and LD surface area is not significantly altered (Figure EV1J). We have now included these data in the revised manuscript, as requested by Reviewer 2.

« Of note, we assessed LD area and found that, while feeding increased LD area, it was not affected by Seipin knockout at this stage, as determined by PLIN1 fluorescence signal quantification (Fig EV1J). » (page 6).

In addition, for the *in vitro* experiments, we have now included in Appendix Figure S4 a detailed assessment of LD number and area and show that neither parameter is significantly altered following siBSCL2 treatment. This is consistent with our previous work[1] using this mature adipocyte model, in which acute Seipin knockdown allows the investigation of early and direct consequences of Seipin deficiency without inducing generalized adipocyte dysfunction within 72 h. The manuscript text has now been revised accordingly.

« Importantly, these changes in contact sites occur while the number and area of mitochondria, LDs, and ER remain unaffected by siBSCL2 (Appendix Fig S4). » (page 6)

b. Throughout their manuscript the authors represent nearly all of their data as bar graphs with no accompanying swarm plot to indicate the data distribution. This is particularly important when relatively small n values are used. For example, it appears that only 2 mice are used with 5 cells per mouse in the first experiments.

We thank the reviewer for this comment and agree that displaying data distribution is particularly important for experiments with relatively small sample sizes. In response, we have revised the graphical representation of the data throughout the manuscript. For small datasets, data are now presented as bar graphs with individual data points overlaid, whereas larger datasets are displayed using violin plots with individual data points. In addition, following the recommendations of the reviewers, we re-evaluated the statistical analyses used throughout the manuscript. Statistical comparisons involving more than two groups are now analyzed using one-way or two-way ANOVA, as appropriate, and the statistical tests used are systematically indicated in the figure legends.

c. PLA is used consistently throughout the manuscript but representative images are rarely, if ever, shown.

We apologize for that, and we are now providing images for each PLA data set.

d. In several places in the manuscript the # of contacts is measured (e.g. Fig. 2C-E). However, it is unclear how this measurement was performed. Is this PLA? If not then it should be stated in the methods how this was performed. For a paper that is mostly based on microscopy, an explanation of how image analyses were performed is severely lacking.

We agree that the description of the image analysis procedures was insufficient in the initial version of the manuscript. As clarified above, the quantification of contact sites was not performed using PLA, but by transmission electron microscopy (TEM)-based analysis of membrane apposition between organelles. To improve clarity and reproducibility, we have now substantially expanded both the Results and Methods sections to provide detailed descriptions of how contact number and contact surface were quantified. We also added a graphical representation of the different parameters measured (Appendix Figure S1). Specifically, contact number corresponds to the occurrence of organelle appositions with an intermembrane distance below 50 nm, while contact surface corresponds to the length of these contacts normalized to the perimeter of the relevant organelle. In addition, figure legends have been revised throughout the manuscript to systematically indicate the nature of the quantified parameter and the corresponding normalization method and clearly state the n number.

2. For the experiment represented in figures 3B, 3C and supplemental 3B, parallel conditions with siNTC should be employed.

This experiment, now presented in Figure EV3G and described in the main text (page 7), was repeated three independent times with a large number of cells analysed in each replicate. Importantly, the observed trend consistently remained non-significant.

« In control cells, MCU knockdown showed a mild trend toward reduced fluorescence recovery, although this did not reach statistical significance (Fig EV3G). » (page 7).

3. The authors consistently use PLA throughout the manuscript as a method of measuring MCSs, but fail to recognize that this method only measures specific interactions and not bulk MCSs. In the discussion, the authors explore how MAM appears to have a different function when it is interacting with LDs. Similarly, different organelle-organelle interactions serve different functions depending on the suite of MCS proteins present. For example, as the authors are likely aware, IP3R-VDAC specifically functions in calcium transfer. In particular, it is strange that the authors chose to use PTPIP51 - PLIN1 as a marker of mito-LD contacts, given that this is not a well-established contact, while MFN2 - PLIN1 is.

We thank the reviewer for this important and insightful comment.

Regarding the use of PLA for MCS measurement: we fully agree that PLA does not measure bulk membrane contact sites (MCSs), but rather the proximity of specific protein pairs. Therefore, PLA reports defined molecular interactions and should not be interpreted as capturing the full spectrum of organelle contacts. We have now clarified this limitation throughout the manuscript and explicitly state

that our PLA analyses reflect changes in specific contact site populations, which may have distinct functional roles depending on their molecular composition (page 5).
In our study, when analyzing contact sites under basal or not already reported models (Figures 1–5), PLA measurements were systematically complemented by transmission electron microscopy (TEM). Across multiple experimental settings, we observed strong concordance between PLA and TEM analyses, supporting the use of PLA as a quantitative proxy for relative changes in contact abundance. Importantly, during the initial optimization experiments using oleate-stimulated differentiated 3T3-L1 adipocytes, we further validated this approach by analyzing each contact type with two independent PLA pairs. These distinct measurements yielded consistent results and were additionally supported by TEM analyses (Appendix Figure S2), reinforcing the robustness of PLA-based measurements. In later figures (Figures 6–7), where contact sites were experimentally manipulated, we relied primarily on PLA as a higher-throughput approach. This choice was supported by the consistency established between PLA and TEM in earlier experiments, as well as by previous studies demonstrating that FATE1 expression and PTPIP51 depletion modulate ER–mitochondria contacts.

To clarify this point, we have modified the manuscript as follows:

"Next, we investigated the impact of the nutritional state on contact remodeling. To this end, we used PLA to quantify contact numbers, as this approach provides a reliable, robust, and rapid method for assessing organelle proximity. IP3R–VDAC PLA was used to quantify MAMs (Tubbs & Rieusset, 2016 [2], while Mi–LD and ER–LD contacts were assessed using PTPIP51–PLIN1 and VAPB–PLIN1 PLA, respectively. Throughout the following analyses, PLA is used to assess proximity between selected protein pairs, providing a proxy for either established contact sites (IP3R–VDAC) or close apposition between organelles (PTPIP51–PLIN1, VAPB–PLIN1). However, in initial optimization experiments using oleate stimulation in 3T3-L1 differentiated adipocytes, we validated our approach by using two distinct PLA pairs for each contact type. These independent measurements yielded consistent results, which were further supported by TEM analyses (Appendix Fig S2).» (page 5).

Regarding the use of PTPIP51–PLIN1 as a marker of Mi–LD contacts: We agree that this interaction is less established than MFN2-based contacts. However, PTPIP51 has been identified in mitochondria-associated fractions[3] and was also detected in our LD proteomic analyses, supporting its potential relevance at Mi–LD interfaces. In addition, we observed strong concordance between PLA and TEM measurements for Mi–LD contacts (e.g., Figures 1–2, Appendix figure S2), further supporting the validity of this approach. Finally, we chose not to rely on MFN2-based PLA assays because MFN2 expression is itself altered under metabolic conditions such as obesity[4], which could confound interpretation of PLA signals independently of actual changes in contact abundance.

We also acknowledge that PLA does not provide information regarding the full molecular composition of contact sites and that distinct contact sites populations likely exert different biological functions depending on their molecular composition. We now discuss this important limitation in the manuscript discussion:

"However, an important limitation of our study that should be addressed in future work is that, as mentioned above, PLA was used to quantify contact sites and, whenever possible, validated by TEM. Yet, we did not investigate the molecular diversity of these contacts, particularly their protein composition. This is important because distinct contact subtypes can support different metabolic functions. For example, in muscle, Perilipin 5–mediated Mi-LD contacts promote fatty acid catabolism [5], whereas in adipocytes, Miga2-mediated Mi-LD contacts favor lipogenesis [3]. Therefore, defining the protein composition of contacts across cellular contexts and nutritional states could help establish a functional cartography of contact subtypes with distinct biological roles. » (page 13).

4. Membrane contact sites are not static but rather dynamic structures that should persist over time. While I appreciate that to achieve the appropriate resolution fixation is preferred, live imaging should be used as an orthogonal experiment to prove that the contacts that the authors are quantifying are actually stable MCSs. This is particularly prudent given that organelle shape and number changes with the experimental setting tested, which could bias a fixed cell analysis towards more false positives as the cellular environment becomes more or less crowded.

We fully agree with the reviewer that live imaging would provide important complementary information regarding the dynamic nature and stability of membrane contact sites. However, live imaging in differentiated adipocytes remains particularly challenging due to the large size and high number of lipid droplets, which generate substantial autofluorescence, increase light scattering, and strongly enhance photobleaching during stack acquisition. These limitations are especially critical when imaging multiple organelles simultaneously in thick cells such as 3T3-L1 adipocytes.

Nevertheless, following the reviewer's suggestion, we performed a pilot live-imaging experiment in differentiated 3T3-L1 adipocytes under basal conditions and after oleic acid (OA) loading. We used green MitoTracker to label mitochondria, LipiTox for lipid droplets, and red ER tracker for the ER. While mitochondrial and LD signals were of satisfactory quality, and while we observed a trend toward increased mitochondria–LD co-localization upon OA treatment consistent with our PLA and TEM data, the ER signal remained of poor quality. Specifically, the ER staining was faint and blurry, and we observed marked photobleaching during acquisition. In z-stack imaging, fluorescence intensity progressively decreased with imaging depth, leading to a loss of more than 70% of the ER signal in the deepest optical sections. This limitation was even more pronounced under OA loading conditions.

We are presenting these preliminary results for the reviewer's consideration only. However, we did not pursue these experiments further because the inability to reliably visualize the ER prevents robust analysis of the central aspect of our study, namely the coordinated regulation of MAMs, ER–LD contacts, and potentially tripartite contacts.

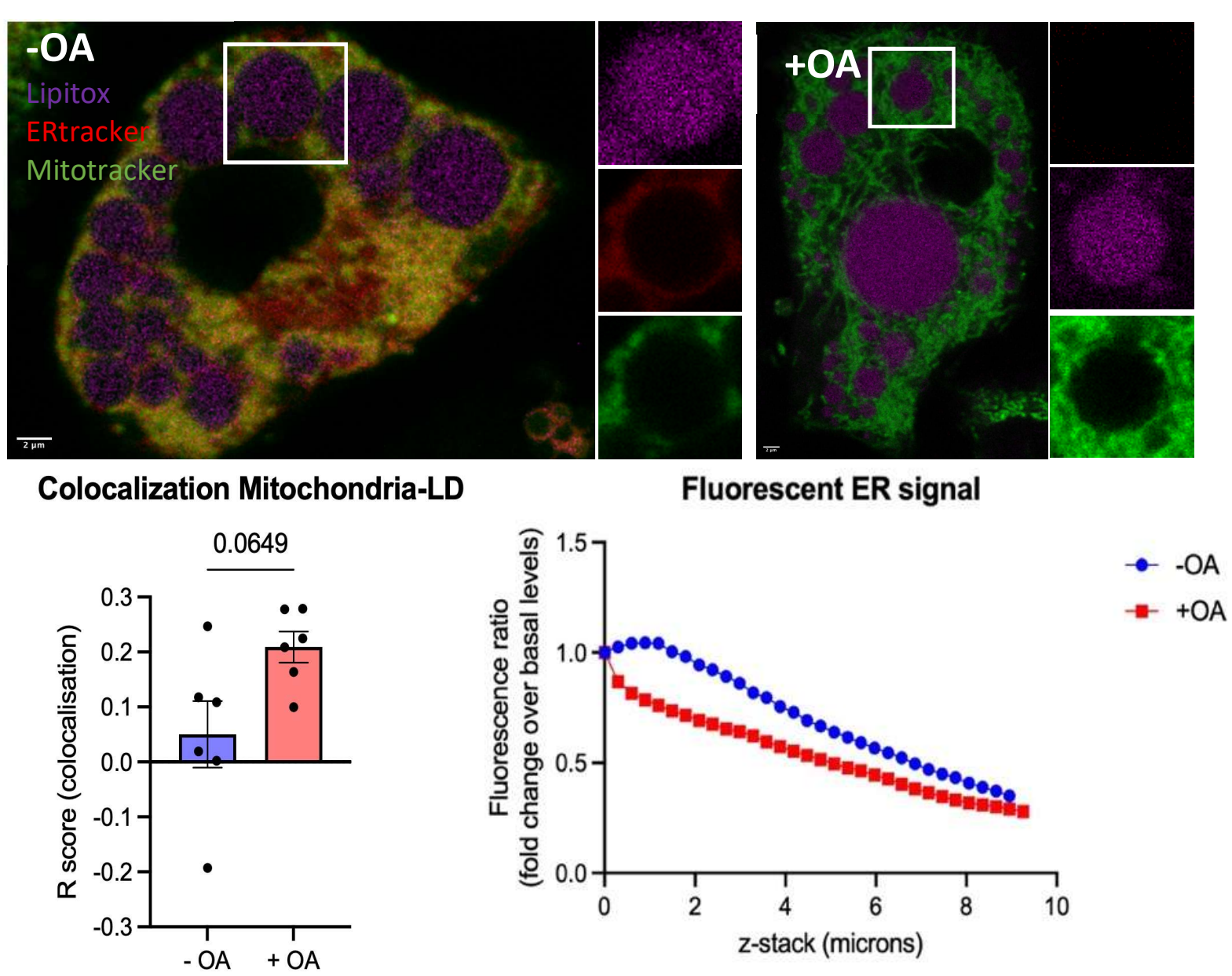

We also explored alternative strategies based on genetically encoded contact reporters. In particular, we attempted to use SPLICS in 3T3-L1 adipocytes. However, we were unable to obtain a satisfactory

fluorescent signal because of the high lipid-associated autofluorescence. In addition, SPLICS expression itself appeared to increase MAM formation, likely reflecting a tethering-related artifact. In parallel, one laboratory within our consortium (Jennifer Rieusset) is currently using the MERLIN system[6], a BRET-based approach, in hepatocytes, and this strategy could potentially be adapted to 3T3-L1 adipocytes. However, MERLIN only provides information on MAMs. More recently, splitFAST-based reporters have been developed for mito–LD and ER–LD contacts[7]; however, no compatible MAM reporter is currently available in this system. Therefore, implementing a unified live-cell imaging strategy capable of simultaneously assessing the three classes of contacts that constitute the core of our study would require substantial methodological development and optimization. This would likely be particularly time-consuming in differentiated adipocytes, where efficient expression of reporters generally relies on viral transduction approaches. For all these reasons, we thought that the dynamic analyses of the tripartite ER-mito-LD contact sites is out of the scope of the present study.

We acknowledge this in the discussion section:

« Another limitation of our study is that we could not directly assess tripartite contact-site dynamics by live imaging, as the optical properties of differentiated adipocytes, particularly their high LD content, prevented robust simultaneous visualization of the ER, mitochondria, and LD. Substantial methodological development and optimization should be performed in the future to access in real-time the interactions between the 3 organelles. » (page 12).

5. I appreciate how PDH phosphorylation is linked with mitochondrial calcium levels, so the experiments in Fig 3F-I provide insights into how much pyruvate is making it into the TCA cycle. However, this work and the experiment depicted in Fig 3J only allow you to draw indirect conclusions about fatty acid metabolism. Please address this in the text or complete further experiments to better measure changes in fatty acid metabolism.

We fully agree with the reviewer; however, we respectfully clarify that we do not draw any conclusions regarding fatty acid oxidation. In this context, PDH phosphorylation is used solely as a readout of mitochondrial calcium levels. We have revised the text accordingly to avoid any confusion.
« Here, PDH phosphorylation was used as an indirect readout of mitochondrial calcium levels, complementing our direct measurements (Fig 3G). While direct measurements of mitochondrial calcium levels were technically not feasible in the adipose tissue of iATSKO mice, PDH phosphorylation levels were increased (Fig 3M-N), supporting the hypothesis that impaired mitochondrial calcium import is a hallmark of Seipin deficiency. » (page 8).

Minor Issues:
1. The authors indicate that a proteomic analysis is performed (Fig. S1H) but no methods or further details are provided for this experiment.

This paragraph has been added in the method section.

***« Proteomic analysis**-Bottom-up mass spectrometry (MS)-based proteomics was used to characterize the proteome. This approach enables the identification and quantification of proteins through the analysis of peptide fragments generated by enzymatic proteolysis. Plasma samples (5 µL) and tissue homogenates (40 µL, 1 mg/mL in PBS) were subjected to tryptic digestion prior to untargeted MS-based proteomic analysis, as previously described [8]. Peptides were identified and quantified by database searching against the Mus musculus reference proteome using Proteome Discoverer (version 2.5; Thermo Fisher Scientific, Villebon-sur-Yvette, France) ». (page 18).*

2. The authors should quantify how the abundance of PDM and CM (independent of their interaction with the ER) changes with Seipin depletion. Also, I encourage the authors to use the term PDM throughout the manuscript.
We are now providing this data in Figure EV1I.

3. Validation of the Seipin knockout and BSCL2 knockdown is missing.
We apologize, and we are now providing these data as mentioned in the manuscript:
" We analyzed subcutaneous AT from inducible adipocyte-specific Seipin knockout mice (iATSKO) 15 days after tamoxifen injection, resulting in a 70% reduction in Bscl2 *mRNA levels (Fig EV1A). » (page 4).*
« To address this, we used 3T3-L1 adipocytes transfected with either non-targeting control siRNA (siNTC) or BSCL2 siRNA (siBSCL2), resulting in a 75% Bscl2 mRNA decrease in 72 hours (Fig EV2A). » (page 6).

4. Figure 1I indicates what each channel represents.
This information has now been included in what is now the figure 1J.

5. In the first section of the results, the authors write "...MAM-CM and MAM-LD levels..." What do the authors mean by "levels" here?
According to the clarification mentioned above, the whole sentence has been rewritten as follows:
« Seipin deficiency reduced the surface area of both MAM–CM and MAM–LD contacts (Fig 1E). However, whereas control mice displayed comparable proportions of MAM–CM and MAM–LD contacts, iATSKO mice exhibited a marked predominance of MAM–CM contacts (~90%), with MAM–LD contacts accounting for only ~10% of total MAMs (Fig 1F) ». (page 5).

6. Figure 2N, appears that the linker conditions are missing from the line graph as they are only present in the accompanying bar graph.
We thank the reviewer for pointing this out. The linker conditions were indeed missing from the line graph in Figure 2N and have now been added for clarity and consistency with the accompanying bar graph.

7. In the second section of the results, the authors write "... both mitochondria and ER appear loosely organized..." A further description of what the authors mean by "loosely organized" would be helpful.
We thank the reviewer for this suggestion. To clarify what we meant by “loosely organized,” we have modified the sentence as follows: « In contrast, in Seipin-deficient cells, mitochondria and the ER no longer closely surround lipid droplets, indicating a loss of tight spatial organization between these organelles (Figure 2K and Videos EV3 and 4). (page 6)

8. The indicators of statistical significance in Fig 4A are very confusing. Please edit this graph to be more intuitive.
We thank the reviewer for this comment. We have modified the figure accordingly to improve clarity and make the statistical indicators more intuitive.

9. Fig 4C - Did you perform statistical analysis on these results? If so, please indicate whether these changes in organelle interaction percentages are significant.
We are now providing statistical analysis.

10. Fig 4J-L: I appreciate the dots indicating the individual data points, but there is no contrast between the data points and the bar colors. Please change one or both of these so you can see the data points that overlap with the bar.
We have now revised Figures 4J–L to allow better visualization of overlapping data points.

11. Supplementary Fig 4F is not cited in the text.
We have replaced this data for histology images in Appendix Fig S6C. We added this sentence: *"Histological analysis confirmed adipocyte hypertrophy in the gonadal white AT (gWAT) of obese mice compared to control lean mice (Appendix Fig S6C)." (page 9)*

12. Fig 5J - The y-axis is just %. Percent of what? Please indicate on the figure.
We thank the reviewer for pointing this out. Following our revised nomenclature and clarification of TEM quantifications, Figures 5D–J now indicate contact number, whereas Figures 5H–K indicate contact area. The y-axis labelling has been corrected accordingly throughout the figure for clarity.

13. I'm not convinced by the statement you make about Figs 6G-I ("... and this decrease is mainly explained by the decreases of MAMs-LD as MAMs-CM are not significantly reduced.") It feels like your n may need to increase because there is a lot of variation in the dataset. More replicates should better reveal what trends there are with more confidence.

We fully agree with the reviewer that the initial dataset was not sufficiently convincing. To address this concern, we have increased the sample size by performing two additional independent experiments. We now include data from 15 full cells across four independent experimental datasets. Importantly, these additional data confirm and strengthen our initial observation: the decrease in total MAM number is primarily driven by a reduction in MAM–LD contacts, while MAM–CM contacts are not significantly affected.

14. The asterisks on Figure 4Q are misaligned.

The alignment of the asterisks in Figure 4Q has been corrected.

15. The authors should acknowledge that several other studies (albeit in different cell types) have previously shown that loss of PTPIP51 alters respiration and calcium flux.
We have cited a previous reference :
"As previously demonstrated in neurons [9], calcium signalling and oxygen consumption were altered in KO cells (Fig EV5E-F)." (page 10)

16. As far as I can tell, supplemental Figure 2 is never referenced.
This dataset is now presented as Figure EV3B and has been appropriately referenced in the Results section as follows:
« SIM analysis with immunolabeling for mitochondria (Tom20), ER (PDI), and LDs (PLIN1), followed by 3D analysis using a 380 nm proximity threshold corresponding to an estimation of the spatial resolution limit of the technique [10] (Fig EV3B) showed that Linker-ER-Mi increases the spatial proximity between ER, mitochondria, and LDs, in the context of Seipin deficiency (Fig 3A-B)) (page 7).

17. Supplemental figures 4 and 5 do not contain any legends for the bar graphs.
These datasets are now presented as Appendix Figure S6 and Figure EV5, respectively. In addition, the figure legends have been revised to include the missing information corresponding to the bar graphs.

18. A summary figure would make interpretation of the results clearer, particularly given that the author's manuscript touches on many different aspects of adipocyte metabolism.
To facilitate interpretation of the results and provide an integrated overview of our findings, we have now included a summary schematic in Figure 8.

19. Many graphs are lacking titles and have vague y-axis labels (e.g., "%", "Ratio"). Providing a simple title would facilitate the interpretation of the results.

We have thoroughly revised the figures to improve clarity, including the addition of more explicit graph titles and more informative y-axis labels throughout the manuscript.

Referee #2:

The authors address an important subject: the mechanism underlying Seipin deficiency effects on membrane contacts and lipid handling. Authors show that MCs, lipid handling, and metabolic flexibility are significantly altered in adipose tissues upon Seipin depletion. These alterations were associated with decreases in both mitochondrial respiration and calcium flux, and could be rescued by reinforcing ER-Mitochondria contacts.

Overall, the manuscript is well written and has a sound conceptualization.

However, it consistently lacked robust, well-defined morphometric quantification methods for membrane contact sites, proper normalization, and appropriate statistics.

1-The authors noted that Seipin-KO in mice results in an 80% reduction in adipose mass, necessitating the use of an earlier time point post-Seipin-KO. However, Authors fail to provide sufficient evidence that this KO phenotype has not yet manifested as a reduction in LD area at their chosen time point. Ideally, mitochondria and LDs should be stained and confocal microscopy used to quantify their gross morphology.

We thank the reviewer for this important comment.

Regarding lipid droplets, we have now quantified LD area using the PLIN1 confocal images shown in Figure 1J and added these data to Figure EV1K. As now indicated in the Results section:

"Of note, we assessed LD area and found that, while feeding increased LD area, it was not affected by Seipin knockout at this stage, as determined by PLIN1 fluorescence signal quantification (Fig EV1J). » (page 6).

These data support our choice of the early time point by showing that the major lipodystrophic phenotype has not yet manifested at the level of LD size.

Regarding mitochondria, we similarly intended to analyze the TOM20 signal in a confocal setup, but the quality and the resolution did not allow proper mitochondria segmentation. To bypass this issue, we quantified the mitochondrial DNA content of EV1G, showing no difference between genotypes.

"We then focused on mitochondrial properties and found a reduced length-to-width ratio (aspect ratio; Fig EV1E) and impaired oxygen consumption in Seipin-deficient AT (Fig EV1F). Of note, these changes occurred despite unchanged mitochondrial DNA content in Seipin-deficient mice (Fig EV1G). » (page 4-5)

In addition, as requested by this reviewer, we measured VDAC1 protein levels in whole tissue extract and observed no differences between genotypes (Appendix Figure S5B).

2-Throughout the manuscript, the authors relied heavily on TEM imaging, severely limiting the study's statistical power. For example, only 10 cells per group were analyzed in the TEM images shown in Figure

1. Robust morphometric methods, complemented with additional vetted techniques, are typically required when TEM is employed to study membrane contact site biology. Proximity ligation assay is a valuable complement to TEM results; however, it must be properly normalized to account for morphological alterations caused by Seipin-KO.

We fully agree with the reviewer that rigorous morphometric characterization is essential when studying membrane contact sites using both TEM and PLA approaches. We acknowledge that this aspect was insufficiently addressed in the initial version of the manuscript. Regarding TEM analyses, we clarify our normalization methods in the following point. Regarding PLA analyses, we have now performed a comprehensive morphometric characterization of lipid droplets (LDs), mitochondria, and the ER in our three main in vitro models. LDs were labelled with BODIPY 505/515, mitochondria with anti-TOM20, and the ER with anti-calnexin antibodies, followed by confocal imaging and segmentation-based quantitative analyses using IMARIS software. These analyses have now been included for Seipin knockdown adipocytes (Appendix Figure S4), FATE1-expressing adipocytes (Appendix Figure S8), and PTPIP51 KO cells (Appendix Figure S9). Although it was not technically feasible to repeat all PLA experiments with normalization based on these newly acquired morphometric datasets, we now systematically present organelle morphology analyses alongside the corresponding PLA results. Importantly, throughout the manuscript, we explicitly indicate when alterations in organelle morphology may influence PLA signal interpretation and therefore require cautious interpretation of contact site quantifications.

The corresponding clarifications have now been added to the relevant Results sections throughout the manuscript.

« Importantly, these changes in contact sites occur while the number and area of mitochondria, LDs, and ER remain unaffected by siBSCL2 (Appendix Fig S4). » (page 6).

« Despite its strong effect in decreasing MAM contacts, FATE1 expression did not modify general mitochondrial or ER parameters, nor did it alter LD properties (Appendix Fig S8). ». (page 9).

« PTPIP51 KO cells display a modest increase in LD area under OA loading, accompanied by a higher frequency of large lipid droplets (Appendix Fig S9). In addition, OA induced a modest increase in mitochondrial and ER volume and area in both control and KO cells. » (page 10).

3-Confusing nomenclature is used throughout the manuscript; for example, the term "MAM-LD" to describe tripartite contacts is ambiguous and could be interpreted as either ER-Mito-LD or Mito-ER-LD contacts. This is not a generally accepted terminology and has been used only once in published literature. It is unclear what organelle contact length means - is it the distance between the apposed organelles, or the length of the regions closely apposed with each other? If the latter is what authors mean, then the reported numbers are well outside the range generally accepted by the field as a bona fide contact site. Regardless, this metric needs to be properly normalized to organelle perimeter or area and presented as percentages.

We acknowledge that, in the initial version of the manuscript, the terminology used to describe contact site parameters in TEM was not sufficiently standardized and that the presentation of the data lacked clarity regarding what was being measured. We have now extensively revised the manuscript to clarify these points throughout the text, figure legends, and “Methods” section. In particular, we added a dedicated paragraph at the beginning of the “Results” section, together with a schematic representation of the quantified parameters (Appendix Figure S1) and a detailed “Methods” section. We now explicitly define the two parameters quantified by TEM analysis as follows:

"Throughout this manuscript, we quantified organelle contact sites using TEM by analyzing ER–mitochondria contacts (i.e., MAMs), ER–lipid droplet (ER–LD) contacts, and mitochondria–LD (Mi–LD) contacts. Two parameters were measured: (1) the contact number, defined as the occurrence of contacts where the distance between organelles is <50 nm, and (2) contact surface, defined as the length of the contact normalized to the mitochondrial perimeter for MAMs and Mi–LD contacts, or to the LD perimeter for ER–LD contacts (as indicated in the axes and figure legends; see Appendix Fig S1 and Methods section). Because this parameter is normalized to the mitochondrial or LD perimeter, it does not simply reflect changes in organelle size or morphology, but rather specifically reports the structural properties of the contact sites. » (page 4).

We also revised our terminology regarding contacts associated with peri-droplet mitochondria to avoid ambiguity. In the revised manuscript, we now explicitly state that "MAM–LD" refers to ER–mitochondria in close proximity to lipid droplets and we are explaining why we choose this nomenclature. Accordingly, the Results section now reads:

« Previous reports have revealed distinct properties of ER–mitochondria contacts involving peri-droplet mitochondria (PDMs), which are closely associated with LDs (<50 nm distance) and have been termed MAM-PDM [11] or MAM–LD [10], compared with contacts involving cytosolic mitochondria (CM), referred to here as MAM–CM [11]. Because our analytical methods do not allow us to distinguish between the ER–Mi–LD (proper MAM-PDM) and Mi–ER–LD configurations, we used the term MAM–LD, which appears more appropriate and less restrictive. Seipin deficiency reduced the surface area of both MAM–CM and MAM–LD contacts (Fig 1E). However, whereas control mice displayed comparable proportions of MAM–CM and MAM–LD contacts, iATSKO mice exhibited a marked predominance of MAM–CM contacts (~90%), with MAM–LD contacts accounting for only ~10% of total MAMs (Fig 1F)» (page 5).

In the Methods section, we further clarified the classification strategy used for these analyses:

"To investigate tripartite contact sites involving ER, mitochondria, and LDs, mitochondria were categorized according to their proximity to LDs. Mitochondria located within 50 nm of a LD were classified as peri-droplet mitochondria (PDM), whereas mitochondria located more than 50 nm away from LDs were classified as cytosolic mitochondria (CM). Contact surface and contact number were then quantified for each mitochondrial subset. ER–mitochondria contacts associated with PDM were designated MAM-LD, whereas those associated with CM were termed MAM-CM. This stratification enabled precise assessment of MAM remodelling according to mitochondrial spatial proximity to LDs." (page 18).

We believe these revisions substantially improve the clarity of the nomenclature and the interpretation of the quantified TEM parameters.

4-Lastly, statistical tests were often used inappropriately. The Mann-Whitney test was misused in several figures that compared more than 2 experimental conditions. The standard practice when using the Mann-Whitney test is to display the data as violin plots with individual data points and ranked quartiles. Comparisons across more than 2 conditions should use one- or two-way ANOVAs, as appropriate. Furthermore, the use of SEM rather than SD is not justified, as the authors reported experimental replicates with N < or = 3.

We thank the reviewer for this important comment. We have carefully revised the statistical analyses throughout the manuscript. In particular, when comparing more than two experimental conditions, we have now applied the appropriate one- or two-way ANOVA tests, as recommended.

In addition, we have revised the data presentation to improve clarity and consistency with standard practices. We have also replaced SEM with SD where appropriate, given the number of experimental replicates.

Minor Comments

All images need scale bars and channel labels. All Western blots need tick marks of the closest molecular weight marker. Western blot quantifications need to be normalized to mitochondrial mass (TOM20/VDAC) or a housekeeping protein (Actin), and/or the total protein stain needs to be shown.

We thank the reviewer for this important comment. We have carefully revised all relevant figures to address these concerns. Specifically, we have added scale bars and channel labels to all microscopy images.

For all Western blots, we now include molecular weight markers with corresponding tick marks (Figures 3K and 3M, Figures 6E and 7E, Figure EV3E, and Appendix Figure S5B). In addition, we improved the clarity of Western blot quantifications by explicitly indicating in each graph axis the housekeeping protein used for normalization. We also now provide total protein staining using Ponceau Red as an additional loading control (Appendix Figures S5A and S7B and C), thereby ensuring appropriate normalization and documentation across samples.

Mitochondrial calcium imaging showing ratios need to be normalized to biomass. The authors need to always state the total number of mice and cells analyzed for each experiment. Nomenclature should be used consistently throughout the manuscript for better clarity. The y-axis should always have easily interpretable titles that clearly state what was measured.

We thank the reviewer for these important comments and have revised the manuscript accordingly to improve clarity and consistency.

Regarding normalization, we agree that normalization to biomass would be ideal. However, direct biomass quantification is not technically feasible in the context of these calcium imaging experiments. Instead, mitochondrial calcium levels were assessed using Rhod-2 fluorescence and expressed as a ratio relative to baseline fluorescence. Because Rhod-2 preferentially labels mitochondrial calcium, this approach partially accounts for variations in mitochondrial content by normalizing changes to the basal fluorescence signal. This is now clearly stated in the figure legends, and the y-axis has been revised accordingly to indicate that the values represent fluorescence normalized to baseline:

« Fluorescence intensity (fold change over basal levels) »

As mentioned in major point #2, to further address this point, we now provide a comprehensive assessment of the mitochondrial network for each cellular model used in the study, which complements the calcium measurements and supports our interpretation.

In addition, we quantified total protein levels (NTC vs siBSCL2) across three independent experiments and did not observe any significant differences. These data are provided for the reviewer's information.

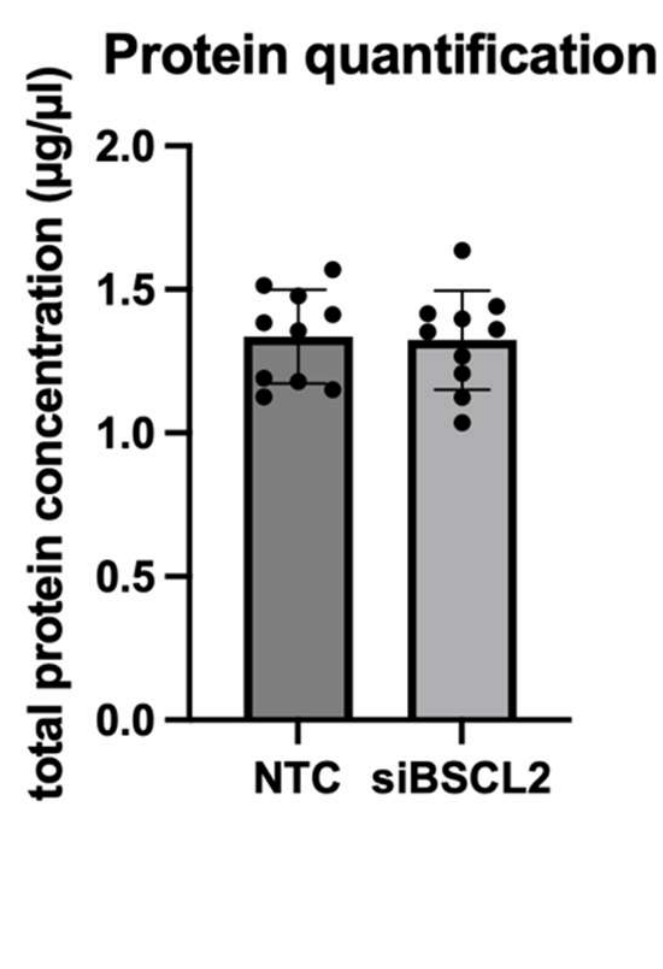

Finally, we have revised all figures to ensure that the total number of cells and animals analyzed is clearly indicated, that nomenclature is used consistently throughout the manuscript, and that all axis labels are explicit and easily interpretable.

Specific comments

Figure1

The comments below are relevant to all other figures that use TEM and PLA to quantify contact sites.

For EM micrographs, it is currently unclear how the number of MAMs, MAM-LD, and MAM-CM was scored. Contact site distances can vary (generally considered <30nm) within a given contact site, so the distribution of distances between organelles at the contact needs to be quantified and shown. Furthermore, contact site lengths are usually expressed as a percentage of the organelle coverage. Given that the Seipin-KO condition alters mitochondrial morphology, the authors should normalize contact site lengths to mitochondrial perimeter or area. Presenting contact site quantification results as total counts can mask the differences in organelle abundance between the control and KO; authors should compare percentages rather than counts. It is often unclear how many cells were analyzed for the quantifications. Reliance on TEM imaging to quantify mitochondrial and LD morphology limits analyses to a single plane. Thus, statistically robust quantification is nearly unattainable, as TEM images do not fully capture a cell's gross morphology.

We thank the reviewer for this detailed and constructive comment. We have clarified and revised our methodology and data presentation accordingly. As indicated in our response to Major Point #3, contact sites were defined based on a distance threshold of <50 nm. We now explicitly state this criterion in the manuscript. In addition, the parameters referred to as "contact" correspond to the surface of contact sites normalized to the relevant organelle geometry: mitochondrial perimeter for MAM and mitochondria–LD (Mi–LD) contacts, and lipid droplet perimeter for ER–LD contacts. This normalization addresses potential differences in organelle morphology, including those observed in the Seipin-deficient condition. To improve clarity and avoid misinterpretation related to organelle abundance, we now systematically report both the normalized contact surface and the number of contacts, as described in the revised manuscript.

We have also clarified the number of cells analyzed for each quantification in the figure legends. Finally, to overcome the limitations inherent to TEM-based analyses, we now provide complementary confocal imaging to assess LD morphology at the whole-cell level and mitochondrial DNA quantification.

The authors did a great job of complementing TEM results by using Proximity ligation, a well-established quantitative technique. However, the total number of PLA dots/puncta needs to be properly normalized to the organelle in question. Since Total MAMs were significantly different between Control and KO, #MAM-LD and MAM-CM should be normalized to total #MAM (Figure1G).

We thank the reviewer for this comment and fully agree that normalization to total MAM abundance is important for proper interpretation of the relative distribution of MAM subpopulations. Accordingly, we have revised Figure 1F and now present MAM–LD and MAM–CM data as percentages of total MAMs rather than absolute values. This normalization allows a more accurate comparison of the relative contribution of each MAM subtype between control and Seipin-deficient conditions, independently of the overall reduction in total MAM abundance.

All images need scale bars and channel labels. For example, it is not clear which channel is cyan in Figure1F, or which channels are blue or red in Figure 1I.

We have revised all images to include scale bars and clear channel labels. In particular, the color assignments (e.g., cyan, blue, red) are now explicitly indicated in the corresponding figure panels and/or legends (Figures 1F and 1G).

The authors say that "MAM was not significantly different between genotypes (Figure 1G)". This should say "MAM-CM was not significantly different between genotypes".

The sentence has been rewritten as follows:
"Seipin-deficient AT showed a marked reduction in the proportion of MAM-LD, while MAM-CM percentage was increased (Figure 1G-H). » (page 5).

The nomenclature "MAM-LD and MAM-CM" is quite ambiguous and can be interpreted as ER-Mito-LD or Mito-ER-LD contacts. For better clarity, the authors should consider changing it to "MAMs-LD distance" < 380nm and > 380nm. The 380nm classification has prior justification in a previous publication, but the authors still need to clearly state this rationale in the text or legend. The Y axes are often labeled too vaguely and can be better clarified by including a short descriptive term that defines what was actually measured. For example, "MAM-LD" can be further defined as "IP3R-PTP51 PLA punctae >380nm from LD". Consider using CTL and iATSKO consistently throughout the manuscript.

As mentioned in response to major point #3, we have now clarified in the manuscript the definition of MAM-LD and the rationale for using this terminology. We have also explicitly justified the use of the 380 nm threshold:

"Then, following a validated protocol [12], we classified ER-Mi contacts as MAM-LD when their distance to LD was below 380 nm; otherwise, they were considered MAM-CM. This threshold was selected on the estimated size of the PLA amplicon. According to the manufacturer, the full PLA signal complex (comprising the primary antibodies, secondary antibodies, and the amplification products generated) has an approximate diameter of 380 nm. Seipin-deficient AT showed a marked reduction in the proportion of MAM-LD, while MAM-CM percentage was increased (Fig 1G-H). (page 5).

We did not modify the Y-axis labels, as we aimed to maintain figure readability. However, the specific PLA pair analyzed is now systematically indicated either in the figure title or in the immediate legend of the corresponding microscopy images, ensuring clarity regarding what was measured.

Finally, the terminology has been harmonized throughout the revised manuscript, and the abbreviations CTL and iATSKO are now used consistently across the main text, figures, and figure legends.

Lipidomics data is pixelated and impossible to see. Lipidomics shows a marked decrease in TG and DG, but, surprisingly, this is not concomitant with reductions in LD area as quantified by TEM. This may demonstrate the limitations of using TEM for quantifying gross differences in LDs and mitochondrial morphology. Authors should use conventional techniques to provide statistically robust quantification of LD and mitochondrial morphology.

We improved the quality of the images.

As mentioned in Major point #1, we have now quantified LD area using the PLIN1 confocal images shown in Figure 1J, and added these data to Figure EV1J.

Figure 2

In 2B-F, it is unclear how the percentage contact and the number of contacts were calculated. In 2I-M, for spatial proximity using SIM, it is unclear how contact sites were quantified. Furthermore, SIM-based methods for measuring proximity are constrained by spatial resolution (typically around ~300nm), which is outside the limits of what the field generally accepts as bona fide contact sites. In other words, spatial proximity analysis is not a direct measure of membrane contact sites. Furthermore, the authors do not state how many cells were analyzed by this technique. Authors use an ER-Mitochondrial linker but do not demonstrate or quantify ER-Mitochondria contacts as proof of concept that the linker increases ER-mitochondria proximity. Instead, Authors quantified ER-LD-Mitochondria proximity, MAMs, MDM-LD, and MAM-CM, and it is currently unclear how this was done. Importantly, the authors used a 380nm threshold to validate membrane contacts, a threshold specific to the proximity ligation technique, which was not used here. Ideally, proximity ligation assays should have been used to complement TEM results here.

In Figure 2N, the authors should include the siNTC-GFP and SiNTC+Linker traces to clearly demonstrate rescue. Also, the linker failed to improve recovery in the NTC background, and this should at least be mentioned in the text, along with the authors' explanation for this observation.

We thank the reviewer for these important comments and have substantially revised the manuscript to clarify both the methodology and interpretation of the analyses presented in former Figure 2, which has now been reorganized between Figures 2 and 3.

First, regarding the TEM quantifications, as mentioned in Major Point #3, we fully revised the presentation of TEM data. To improve clarity, contact surface measurements are now presented in Figure 2E-I, whereas contact number measurements are presented separately in Figure EV2C-G. The corresponding definitions and quantification procedures are now extensively detailed in the Methods section and illustrated in Appendix Figure S1.

Regarding the SIM analyses, now presented in Figure 3A-B, we fully agree that SIM-based proximity analysis does not provide a direct measurement of membrane contact sites at nanometer resolution. The 380 nm threshold used in our analyses was initially derived from previous PLA/SIM-based studies and also corresponds closely to the spatial resolution limit of SIM (~300 nm), as correctly pointed out by the reviewer. For consistency, we therefore used the same threshold throughout all SIM analyses. Importantly, in the revised manuscript, the organization of the figures has been modified to better separate SIM-derived data (Figure 3A-B) from TEM-derived data (Figure 3C-E), which may indeed have been confusing in the original version.

The revised text now reads:

"Next, we expressed the Linker-ER-Mi in Seipin-deficient adipocytes and we used Structured illumination microscopy (SIM) to assess the effect on MAM-LD. SIM analysis with immunolabeling for mitochondria (Tom20), ER (PDI), and LDs (PLIN1), followed by 3D analysis using a 380 nm proximity threshold corresponding to the spatial resolution limit of the technique [10] (Fig EV3B) showed that Linker-ER-Mi increases the spatial proximity between ER, mitochondria, and LDs, in the context of Seipin deficiency (Fig 3A-B). TEM further confirmed that Linker-ER-Mi expression reestablished MAM structures, particularly MAM-LD contacts (Fig 3 C-E, Fig EV3C). » (page 7).

We also clarified the number of analyzed cells in the figure legends.

Importantly, in response to the reviewer's suggestion to complement TEM analyses with PLA, we now provide additional PLA data in Figure EV3A validating the functionality of the ER–mitochondria linker. Specifically, we quantified VDAC-IP3R PLA signal in 3T3-L1 adipocytes and observed that Linker-ER-Mi expression increased ER–mitochondria proximity. The "Results" section now states:

"First, we validated, using PLA, that expression of the Linker-ER-Mi increased MAM abundance (VDAC-IP3R) in 3T3-L1 adipocytes (Fig EV3A). » (page 7).

Finally, regarding Figure 3F (former Figure 2N), we have now added the siNTC-GFP and siNTC+Linker traces to the revised figure. We also added the following sentence to the "Results" section:

"Of note, the Linker-ER-Mi has no effect on fluorescence recovery in siNTC-treated cells." (page 7).

Figure 3

In Figures 3A-C, it is unclear how fluorescence ratios and % recovery were calculated. This needs to be restated regardless of previous figures. In 3D-E, it is unclear what ratios are calculated for spatial analyses. Western blots need a tick mark for the molecular weight of the closest protein ladder marker. In addition, p-PDH/PDH ratios should be further normalized to a mitochondrial mass marker to account for differences in total mitochondria

We are now providing this data and added the following sentence in the text:
"To ensure that variations in PDH levels were not due to differences in total mitochondrial mass, we also quantified VDAC1 expression as a mitochondrial marker and observed no differences (Appendix Fig S5B)." (page 8).

Citrate enrichment needs to be properly normalized to biomass.

As mentioned for the calcium data, we are not able to provide a direct assessment of biomass in these experiments, as only polar metabolites were extracted. However, we have quantified total protein levels (NTC vs siBSCL2) across three independent experiments and did not observe any significant differences. These data are provided for the reviewer's information.

Figure4 and 5

Better clarification is needed regarding how # of contact sites was determined by TEM. Since nutrient perturbations most likely alter mitochondrial and LD morphologies, PLA dots should be normalized to mitochondrial or LD perimeter (2D segmentation) or area (3D segmentation), as appropriate.

As mentioned previously, we have fully revised the TEM nomenclature, and the symbol "#" is no longer used.

Contact site nomenclature should be consistent (e.g., mito-LD, not mito/LD).

We have now been very careful to ensure consistency in the terminology used throughout the manuscript.

It is unclear how tripartite contacts were quantified.
As indicated in our response to Major Point #3, we have clarified this point in the revised version of the manuscript.

Images need scale bars. In Figure 5, the total contact length was measured, but the authors need to clarify how it was normalized.
We added the scale bars. We fully revised our TEM nomenclature.

Figures 6 and 7
PLA dots should be normalized to mitochondrial or LD perimeter (2D segmentation) or area (3D segmentation), as appropriate. Western blots need to be normalized to the total protein loaded. Glycerol concentration needs to be normalized to biomass.

We thank the reviewer for this important comment. Glycerol measurements for lipolysis assessment are now normalized to total protein levels. In addition, Phospho-AKT levels are normalized to β-actin as a loading control.

Regarding PLA quantification, we acknowledge the reviewer's suggestion to normalize to organelle perimeter or area. While we have not applied this normalization to the PLA data, we have strengthened our analysis in the revised manuscript by providing quantitative assessments of lipid droplets, ER, and mitochondria using confocal imaging. These additional data allow us to account for potential differences in organelle abundance and support the interpretation of the PLA results.

Supplemental
S1: It needs to be stated how the LD area was normalized.
As mentioned previously, this has been changed and done.

S2: Images need scale bars, channel labels, and boxes indicating the ROIs that were magnified.
We have revised the figures accordingly.

S3: MCU knockdown needs to be quantitatively validated at the protein level.
These data are now presented in figure EV3E and the "Results" section indicates the following:
"We confirmed that siMCU effectively reduced MCU mRNA and protein (Figure EV3D-E) levels" (page 7)

S4: Glycemia levels need to be normalized to weight. Scale bars needed for images.

For GTT and ITT, our laboratory now follows recently published guidelines that do not recommend normalizing glycemia to body weight[13]. However, the administered glucose dose was normalized to body weight.

S5: Western blot and Oil red staining need quantification. Mitochondrial calcium and OCR measurements need to be normalized to biomass.

We have now included quantification of the western blot data. Unfortunately, the Oil Red O images were acquired using a low-quality scanner, which does not allow reliable quantification, and we apologize for this limitation. As an alternative, we have added mRNA expression data for key adipocyte differentiation and mature adipocyte marker genes to further support the differentiation status of the cells.

Referee #3:

The manuscript by Pallard et al. "Tripartite ER mitochondria lipid droplet contact sites control adipocyte metabolic flexibility" represents a signifant conceptual advance. Contact sites between

several organelles are known to be important for cell function but their identity as dynamic hubs in the control of metabolic activity of various cells such as adipocytes remains poorly understood. Pallard et al demonstrate in that adipocyte function in fat storage depends on the interplay of at least three organelles including the ER, mitochondria and lipid droplets.

Overall this is an extremely timely study, rich in novelty and conceptually creative. The paper starts with the investigation of Seipin as an important factor in lipid droplet maintenance within adipose tissue. The authors introduce a new experimental paradigm exploiting the inducible knock out of Seipin in mouse.

The authors investigate the Seipin KO mice already 15 days after induction of the gene knockout. Loss of Seipin function causes lipodystrophy a condition marked by the absence of functional white adipose tissue but the mechanisms that lead to the defect and the cell biological function of Seipin in adipose tissue is not yet clear. With the new approach the authors shed light on the mechanisms by which Seipin forms a contact site between the ER and mitochondria and clarify how calcium flux through this interface influences lipid flux to the LDs.

The strength of the paper is the creative interpretation of the data and the multimodal approach. Contact site formation is modulated and assessed in multiple ways. The EM is impressive and extensively quantified, combined with fluorescence microscopy and metabolic assays. While this is the strength of the work it sometimes turns into a problem because the early and later figures seem disconnected in content and approach. This needs to be more consistent before publication.

The major conclusion is interesting. Seipin dependent contact sites between mitochondria and the ER control calcium accumulation in the mitochondria. Although previously reported by the Prieux lab its identification as a metabolic switch is novel: The contact site switches ON pyruvate dehydrogenase as well as enzymes in the Krebs cycle to synthesise citrate as a precursor for fatty acid and triglyceride biosynthesis.

In obesity and under high glucose conditions the contact sites break similar as in Seipin depleted adipose tissue which is of considerable conceptual novelty. Despite the lack of a precise molecular mechanism the physiology is compelling. The paper is therefore a good contender for EMBO journal. The quality of figures and the coherence across figures may be improved as a condition for publication.

Specific Comments

The conclusion in the first part of the paper depends on the fluorescence recovery assay using triglycerides labelled with fluorescent fatty acids. Loss of Seipin slightly attenuates the recovery after bleaching (100 sec long experiments). It is unclear how the authors explain this effect mechanistically. Is this working through calcium? If yes, why would the effect be so fast (100 sec recovery). If not how would depletion of Seipin stop lipid exchange with the ER? Recent work on Seipin suggests that inactivation of Seipin through adipogenin activates lipid storage in adipocytes. This part of the paper is conceptually and mechanistically not sound and needs improvement. For example a simple way to test whether triglyceride synthesis is required (calcium dependence) is to add DGAT inhibitors and see whether the recovery does not work. This would support a defect in de novo synthesis of TGs. If the TGs just move to the LDs and this is blocked without Seipin the authors need to explain where the TGs are stored before movement.

We thank the reviewer for this important comment.

First, we would like to clarify that although the FRAP acquisition is performed over ~100 seconds, the fluorescent signal reflects triglycerides that were synthesized and incorporated into lipid droplets during the prior loading phase. Therefore, the recovery measured during FRAP does not report acute triglyceride synthesis within this short time window, but rather the dynamics of an already established fluorescent TG pool. In this context, our data suggest that Seipin deficiency alters the availability of triglycerides prior to the FRAP measurement. One possible mechanism is that impaired ER–mitochondria calcium transfer reduces citrate production, thereby limiting fatty acid synthesis and subsequent triglyceride synthesis. We note that this remains a working hypothesis, consistent with our observation that the ER–mitochondria linker restores both citrate levels and TG flux.

To directly address the reviewer's suggestion regarding the role of de novo TG synthesis, we performed experiments using DGAT1 and DGAT2 inhibitors. When inhibitors were applied during the fluorescent fatty acid loading phase, lipid droplets failed to become fluorescent, precluding FRAP analysis. This indicates that incorporation of fluorescent fatty acids into TGs via DGAT activity is required to generate the measurable pool. In contrast, when DGAT inhibitors were added only during the FRAP acquisition, fluorescence recovery was unaffected. This demonstrates that ongoing TG synthesis is not required during the FRAP time window itself, and supports the idea that the observed recovery reflects redistribution or exchange within a pre-existing TG pool rather than acute synthesis.

Together, these results indicate that TG synthesis is essential for establishing the fluorescent pool prior to FRAP, but does not directly contribute to recovery kinetics during the assay. This is consistent with our interpretation that Seipin primarily affects upstream metabolic processes influencing TG availability, rather than acute lipid transfer during the FRAP measurement.

We added this sentence in the result section:
"Of note, inhibition of triglyceride synthesis enzymes (DGAT1 and DGAT2) during the BODIPY-C12 fluorescent fatty acid loading phase altered the cellular labeling pattern, preventing large and medium-sized LDs from becoming fluorescent. This confirms that incorporation of the probe depends on triglyceride synthesis during this step (Fig. EV2H). In contrast, inhibition of DGAT1/2 when applied only during the FRAP assay did not affect fluorescence recovery (Fig EV2I). These findings indicate that the observed fluorescence recovery does not depend on ongoing triglyceride synthesis during the assay itself, but rather on prior incorporation of the fluorescent fatty acid into triglycerides during the loading phase." (page 7).

We added this sentence in the discussion:
"In addition, when DGAT inhibitors were used, lipid loading during FRAP was abolished, supporting that the observed phenomenon relies on de novo TG synthesis." (page 11).

A next point which needs explanation is which subtype of the various MAM-LD contact sites is important. This reviewer is not clear about which contact site is under the control of Seipin. The confusion mainly arises because the paper moves from Seipin to PTPIP51 and then to FATE. Neither of these proteins acts together with Seipin. The authors use them as tools to modulate the contact sites but the conclusions regarding Seipin function are not clearly set out.

We thank the reviewer for this important comment. We fully acknowledge that this is a key limitation of our study and appreciate the opportunity to clarify this point.

Our intention was not to suggest that Seipin directly interacts with, or acts through, PTPIP51 or FATE1. These proteins were used solely as experimental tools to modulate ER–mitochondria contact abundance and assess the functional consequences of altering these contacts. We agree that this

distinction was not sufficiently clear in the original version and have now clarified this point in the revised manuscript (page 13).

Importantly, the approaches used in this study (primarily PLA, complemented when possible by TEM) allowed us to quantify the contact abundance and spatial organization, but not to define the precise molecular identity or protein composition of the distinct MAM–LD contact subtypes regulated by Seipin. Addressing this question would require a much deeper characterization of these contact sites, including their proteomic composition and how this may vary across nutritional or metabolic states. This is currently an active area of investigation in our laboratory.

We have now explicitly discussed this limitation in the discussion section of the manuscript:

« However, an important limitation of our study that should be addressed in future work is that, as mentioned above, PLA was used to quantify contact sites and, whenever possible, validated by TEM. Yet, we did not investigate the molecular diversity of these contacts, particularly their protein composition. This is important because distinct contact subtypes can support different metabolic functions. For example, in muscle, Perilipin 5–mediated Mi-LD contacts promote fatty acid catabolism [5], whereas in adipocytes, Miga2-mediated Mi-LD contacts favor lipogenesis [3]. Therefore, defining the protein composition of contacts across cellular contexts and nutritional states could help establish a functional cartography of contact subtypes with distinct biological roles.» (page 13).

The contact site markers for the proximity labelling require validation. Is the localization of organelle markers suitable for the assay. Total MAM markers IP3R-VADAC are validated in the literature but this reviewer is not aware of an interaction between VAP proteins and PLIN1 as well as PTPIP51 and PLIN1. How were these partners validated and are they interacting or simply marking the organelles that are in close apposition?

We thank the reviewer for this important comment regarding the validation and interpretation of the PLA markers used to assess organelle contacts. We agree that it is essential to distinguish between PLA pairs representing established molecular contact sites and those used more broadly as proxies for organelle proximity. As now clarified in the revised manuscript, IP3R–VDAC PLA was used to quantify ER–mitochondria contacts (MAMs), as this interaction is well established in the literature (Tubbs & Rieusset, 2016). In contrast, for Mi–LD and ER–LD contacts, our intention was not to imply direct or functionally established molecular interactions between PTPIP51 and PLIN1, or between VAPB and PLIN1. Rather, these PLA pairs were selected as markers of close apposition between the corresponding organelles, allowing us to assess relative changes in organelle proximity. To make this distinction explicit, we have added the following clarification to the manuscript: *"Throughout the following analyses, PLA is used to assess proximity between selected protein pairs, providing a proxy for either established contact sites (IP3R–VDAC) or close apposition between organelles (PTPIP51–PLIN1, VAPB–PLIN1). »* (page 5)

Regarding marker selection, PTPIP51 was chosen because it is a mitochondrial-associated protein identified in mitochondria-associated fractions[3] and was also detected in our lipid droplet proteomic analyses, supporting its relevance at Mi–LD interfaces. We deliberately did not rely on MFN2-based PLA assays, although MFN2 has been implicated in Mi–LD contacts, because MFN2 expression is itself altered under metabolic conditions such as obesity[4], which could confound interpretation of PLA signals independently of actual contact abundance.

Importantly, we also experimentally validated our PLA-based approach during assay optimization in differentiated 3T3-L1 adipocytes by testing two independent PLA pairs for each contact type. These

distinct PLA measurements yielded consistent results and showed strong concordance with TEM-based quantification, supporting the robustness of the approach. Given that this validation is critical for the interpretation of our data, as appropriately pointed out by the reviewer, we have now included these validation data in Appendix Figure S2.

We fully acknowledge, however, that PLA does not define the precise molecular composition of individual contact sites, but rather provides a robust readout of organelle proximity dynamics.

Minor comments

The figure labelling should be revised and double checked.
We have thoroughly revised the nomenclature, axis labeling, graph titles, and figure legends throughout the manuscript.

Fig. 1 H: plasmic membrane - plasma membrane
This has now been corrected.

The title in Fig. 2 is confusing: Please correct the language.
We have changed the title to:
Seipin deficiency alters MAM–LD contacts and disrupts TG flux toward lipid droplets

Fig. 3: calcium dependent.
This has now been corrected.

Fig. 4: it is unclear what J0, J2...J8 reflect in Fig. 4. This reviewer suspects that this is an abbreviation for the word jour in French meaning day. The figure should be labelled in Englisn
This has now been corrected.

Fig. 7: MAM re-inforcement in MCS altered cellular model....- this is incomprehensible. Please rewrite.
We have changed the title to:
MAM reinforcement is sufficient to restore adipocyte properties

1. Combot, Y., Salo, V.T., Chadeuf, G., Holtta, M., Ven, K., Pulli, I., Ducheix, S., Pecqueur, C., Renoult, O., Lak, B., et al. (2022). Seipin localizes at endoplasmic-reticulum-mitochondria contact sites to control mitochondrial calcium import and metabolism in adipocytes. Cell Rep *38*, 110213. 10.1016/j.celrep.2021.110213.
2. Tubbs, E., and Rieusset, J. (2016). Study of Endoplasmic Reticulum and Mitochondria Interactions by In Situ Proximity Ligation Assay in Fixed Cells. J Vis Exp. 10.3791/54899.
3. Freyre, C.A.C., Rauher, P.C., Ejsing, C.S., and Klemm, R.W. (2019). MIGA2 Links Mitochondria, the ER, and Lipid Droplets and Promotes De Novo Lipogenesis in Adipocytes. Mol Cell *76*, 811-825 e814. 10.1016/j.molcel.2019.09.011.
4. Mancini, G., Pirruccio, K., Yang, X., Bluher, M., Rodeheffer, M., and Horvath, T.L. (2019). Mitofusin 2 in Mature Adipocytes Controls Adiposity and Body Weight. Cell Rep *26*, 2849-2858 e2844. 10.1016/j.celrep.2019.02.039.
5. Kimmel, A.R., and Sztalryd, C. (2014). Perilipin 5, a lipid droplet protein adapted to mitochondrial energy utilization. Curr Opin Lipidol *25*, 110-117. 10.1097/MOL.0000000000000057.
6. Hertlein, V., Flores-Romero, H., Das, K.K., Fischer, S., Heunemann, M., Calleja-Felipe, M., Knafo, S., Hipp, K., Harter, K., Fitzgerald, J.C., and Garcia-Saez, A.J. (2020). MERLIN: a novel BRET-based proximity biosensor for studying mitochondria-ER contact sites. Life Sci Alliance *3*. 10.26508/lsa.201900600.

7. Li, X., Gamuyao, R., Wu, M.L., Cho, W.J., King, S.V., Petersen, R.A., Stabley, D.R., Lindow, C., Climer, L.K., Shirinifard, A., et al. (2024). A fluorogenic complementation tool kit for interrogating lipid droplet-organelle interaction. J Cell Biol *223*. 10.1083/jcb.202311126.
8. Rodrigues Oliveira, A., Chevalier, C., Wargny, M., Pakulska, V., Caradeuc, C., Cloteau, C., Letertre, M.P.M., Giraud, N., Bertho, G., Bigot-Corbel, E., et al. (2025). Methylglyoxal-Induced Glycation of Plasma Albumin: From Biomarker Discovery to Clinical Use for Prediction of New-Onset Diabetes in Individuals with Prediabetes. Clin Chem *71*, 688-699. 10.1093/clinchem/hvaf035.
9. Gomez-Suaga, P., Perez-Nievas, B.G., Glennon, E.B., Lau, D.H.W., Paillusson, S., Morotz, G.M., Cali, T., Pizzo, P., Noble, W., and Miller, C.C.J. (2019). The VAPB-PTPIP51 endoplasmic reticulum-mitochondria tethering proteins are present in neuronal synapses and regulate synaptic activity. Acta Neuropathol Commun *7*, 35. 10.1186/s40478-019-0688-4.
10. Guyard, V., Monteiro-Cardoso, V.F., Omrane, M., Sauvanet, C., Houcine, A., Boulogne, C., Ben Mbarek, K., Vitale, N., Faklaris, O., El Khallouki, N., et al. (2022). ORP5 and ORP8 orchestrate lipid droplet biogenesis and maintenance at ER-mitochondria contact sites. J Cell Biol *221*. 10.1083/jcb.202112107.
11. Najt, C.P., Adhikari, S., Heden, T.D., Cui, W., Gansemer, E.R., Rauckhorst, A.J., Markowski, T.W., Higgins, L., Kerr, E.W., Boyum, M.D., et al. (2023). Organelle interactions compartmentalize hepatic fatty acid trafficking and metabolism. Cell Rep *42*, 112435. 10.1016/j.celrep.2023.112435.
12. Monteiro-Cardoso, V.F., Le Bars, R., and Giordano, F. (2023). Visualization and Quantification of Endogenous Intra-Organelle Protein Interactions at ER-Mitochondria Contact Sites by Proximity Ligation Assays. J Vis Exp. 10.3791/64750.
13. Moro, C., and Magnan, C. (2025). Revisited guidelines for metabolic tolerance tests in mice. Lab Anim (NY) *54*, 16-23. 10.1038/s41684-024-01473-5.

1st Revision - Editorial Decision 1st Jul 2026

Dear Xavier,

Your revised manuscript was sent to the three original reviewers, I have now received re-review reports from two of them, which I have included below. As you will see, you have addressed their concerns satisfactorily. Before I can finally accept the manuscript, there are some remaining editorial points which need to be addressed. In this regard would you please:

- label main and EV figure legends;
- rename Expanded View Figure 1-5 as Figure EV1-EV5
- rename the 'Conflict of interest statement' to the 'Disclosure and Competing Interests Statement', acknowledging employment in a biotech company therein,
remove the AC/CrediT section from the text,
- list all figure callouts in the manuscript sequentially, including a callout for Fig. 8,
- re-upload EV figures as individual, high-resolution figure files,
- include an appendix title page containing the title "Appendix for Tripartite ER-Mitochondria-Lipid Droplets contact sites control adipocyte metabolic flexibility" (an author and affiliation list is not necessary here) and a table of contents with the page numbers for the listed items; here, the nomenclature should be Appendix Figure Sx and Appendix Table Sx throughout the manuscript and Appendix PDF; figure legends should be placed below the corresponding figure,
- save Source data files in a scheme of one figure/folder and then uploaded as .zip files. E.g. all the Source data files for figure 1 need to be saved in a single folder and this needs to be zipped and then uploaded as "SD figure 1.zip" file,
- provide a synopsis image in .jpg or .png format, exactly 550 pixels wide and 300 pixels high ensuring that the text is legible at the final size and that the synopsis image has appropriate proportions,
- provide suggestions for a short 'blurb' text prefacing and summing up the conceptual aspect of the study in two sentences (max. 250 characters), followed by 3-5 one-sentence 'bullet points' with brief factual statements of key results of the paper; they will form the basis of an editor-written 'Synopsis' accompanying the online version of the article,
- list reuse of images in Figure 3A and Figure EV3B in the EV3 figure legend,
- provide exact p values in the legends of 1B-F,H,K-M; 2B-I,M; 3B-H,J,L,N,O; 4A-H,J-O; 5A-K; 6A-G,I; 7A-H; EV-1A-J; EV-2A,C,E,F, EV-3A,D,E,F; EV-4D,F; EV-5A,C,E,F,G,H;
- define box plots in terms of minima, maxima, bounds of box and whiskers, and percentile in the legends of figure 5D-G; EV-1B-D; EV-2C-G; EV-4D,F,
define error bars in the legends of figures 1E,F,
- rename movie files as Movie EV1-EV4 with the corresponding callouts in the manuscript text, remove legends from Appendix PDF and zip with each movie file,
- rename "Materials and Methods" as "Methods", and
- use the following section order: Title page - Abstract - Introduction - Results - Discussion - Methods - Data Availability - Acknowledgements - Disclosure and Competing Interests Statement - References - Figure Legends - Table(s) - Expanded View Figure Legends.

I am looking forward to receiving your revised manuscript.

EMBO Press is an editorially independent publishing platform for the development of EMBO scientific publications.

Best wishes,

William

William Teale, PhD
Editor
The EMBO Journal
w.teale@embojournal.org

Instructions for preparing your revised manuscript:

Read our guidance for manuscript revisions and related editorial policies: https://link.springer.com/journal/44318/submission-guidelines#cms-Revised-submissions

Please also check that the title and abstract of the manuscript are brief, yet explicit, even to non-specialists.

When assembling figures, please refer to our figure preparation guideline in order to ensure proper formatting and readability in print as well as on screen:

https://media.springernature.com/original/springer-cms/rest/v1/content/27825798/data/v1

IMPORTANT: When you send the revision we will require
- a point-by-point response to the referees' comments, with a detailed description of the changes made (as a word file).
- a word file of the manuscript text.
- individual production quality figure files (one file per figure)
- a complete author checklist
- Expanded View files (replacing Supplementary Information)
- a Reagents and Tools Table as part of the Methods section

Please remember: Digital image enhancement is acceptable practice, as long as it accurately represents the original data and conforms to community standards. If a figure has been subjected to significant electronic manipulation, this must be noted in the figure legend or in the 'Methods' section. The editors reserve the right to request original versions of figures and the original images that were used to assemble the figure.

We realize that it is difficult to revise to a specific deadline. In the interest of protecting the conceptual advance provided by the work, we recommend a revision within 3 months (29th Sep 2026). Please discuss the revision progress ahead of this time with the editor if you require more time to complete the revisions. Use the link below to submit your revision:

https://emboj.msubmit.net/cgi-bin/main.plex

Referee #1:

I commend the authors on a very thorough response. The additional studies and analysis have greatly improved the quality and readability of this manuscript. This is very exciting work that reframes our understanding of seipin and LD biogenesis - congratulations.

Referee #3:

The authors have addressed most of the issues raised by this reviewer.

The figures have significantly improved.
The scientific value is the detailed analysis of adipocyte ultrastructure. I appreciate this exceptional effort.
The mechanism which is suggested is plausible but I cannot follow the logic completely; the work will stimulate significant discussion in the field.

1. Label main and Expanded View figure legends.
Response: We added the appropriate labels at the beginning of both the Figure Legends and Expanded View Figure Legends sections.

2. Rename Expanded View Figure 1–5 as Figure EV1–EV5.
Response: We renamed the Expanded View figures as requested.

3. Rename the "Conflict of interest statement" to the "Disclosure and Competing Interests Statement", acknowledging employment in a biotech company therein, and remove the AC/CrediT section from the text.
Response: We renamed the section to **Disclosure and Competing Interests Statement** and updated it as follows:

« All authors declare no competing interests related to this study. A.R.T. is cofounder and advisor of Oria Bioscience. »

We also removed the AC/CrediT section from the manuscript.

4. List all figure callouts in the manuscript sequentially, including a callout for Figure 8.
Response: We carefully checked the manuscript to ensure that all figures are cited sequentially. We also added a callout for Figure 8 at the end of the first paragraph of the Discussion:

« Altogether, we highlight the key role of MAM–LD in lipid handling and the importance of membrane contact sites involving the ER, lipid droplets, and mitochondria in metabolic flexibility. A working model summarizing our findings is presented in Figure 8. »

5. Re-upload Expanded View figures as individual high-resolution figure files.
Response: The files have been uploaded.

6. Include an Appendix title page containing the title "Appendix for *Tripartite ER-Mitochondria-Lipid Droplets contact sites control adipocyte metabolic flexibility*", together with a table of contents; use the nomenclature Appendix Figure Sx and Appendix Table Sx throughout; place figure legends below the corresponding figures.
Response: We implemented all of these requested modifications and uploaded the new file.

7. Save Source Data files as one folder per figure and upload them as zipped files (e.g., "SD Figure 1.zip").
Response: The Source Data files have been reorganized and uploaded according to the requested format.

8. Provide a synopsis image in .jpg or .png format (550 × 300 pixels).
Response: The requested synopsis image has been prepared and uploaded according to the specified dimensions.

9. Provide a short blurb and bullet points for the editor-written Synopsis.
Response: We propose the following text.

Blurb

Adipocyte lipid loading drives remodeling of tripartite ER–mitochondria–lipid droplet contacts. Loss of this remodeling in Seipin deficiency and obesity is associated with adipocyte dysfunction and impaired metabolic flexibility.

Bullet points

- Lipid loading remodels adipocyte membrane contact sites by increasing tripartite ER–mitochondria–lipid droplet contacts while reducing ER–mitochondria contacts distant from lipid droplets.
- Seipin deficiency abolishes this nutrient-induced remodeling and impairs triglyceride transfer from the ER to lipid droplets.
- Reinforcing ER–mitochondria contacts with a synthetic linker restores ER-to-lipid droplet triglyceride flux in Seipin-deficient adipocytes through a calcium-dependent mechanism.
- Obesity is associated with loss of nutrient-dependent membrane contact site remodeling in adipose tissue.
- Genetic disruption of membrane contact sites impairs lipid transfer to lipid droplets, lipolysis, and insulin signaling in adipocytes.

10. List reuse of images in Figure 3A and Figure EV3B in the Figure EV3 legend.
Response: We added the following statement to the Figure EV3B legend:

Selected spot representations are reused from Figure 3A.

11. Provide exact *P* values in the requested figure legends; define box plots in the specified legends; define error bars in the legends of Figures 1E and 1F.
Response: We revised all requested figure legends by providing the exact *P* values where requested, defining the box plots (minimum, maximum, box boundaries, whiskers, and percentiles), and specifying the meaning of the error bars in Figures 1E and 1F.

12. Rename movie files as Movie EV1–EV4, include corresponding callouts in the manuscript, remove legends from the Appendix PDF, and zip each movie with its corresponding files.
Response: We renamed the movie files as requested, updated the corresponding callouts in the manuscript, removed the movie legends from the Appendix PDF, and uploaded each movie as a separate zipped archive.

13. Rename "Materials and Methods" as "Methods".
Response: We renamed the section to **Methods**.

14. Use the following section order: Title page – Abstract – Introduction – Results – Discussion – Methods – Data Availability – Acknowledgements – Disclosure and Competing Interests Statement – References – Figure Legends – Tables – Expanded View Figure Legends.
Response: We reorganized the manuscript to follow the requested section order, including moving the **Acknowledgements** section accordingly.

2nd Revision - Editorial Decision 9th Jul 2026

Dear Xavier,

I am pleased to inform you that your manuscript has been accepted for publication in the EMBO Journal.

Congratulations to all involved!

Your manuscript will be processed for publication by EMBO Press. It will be copy edited and you will receive page proofs prior to publication. Please note that you will be contacted by Springer Nature Author Services to complete licensing and payment information.

You may qualify for financial assistance for your publication charges - either via a Springer Nature fully open access agreement or an EMBO initiative. Check your eligibility: https://link.springer.com/journal/44318/how-to-publish-with-us

Should you be planning a Press Release on your article, please notify the production team via this form in order to coordinate publication and release dates: https://forms.cloud.microsoft/e/eH4A8JVP6g

If you have any questions, please do not hesitate to contact the Editorial Office. Thank you for your contribution to The EMBO Journal.

Best wishes,

William

William Teale, PhD
Editor
The EMBO Journal
w.teale@embojournal.org

*****
Please note that it is The EMBO Journal policy for the transcript of the editorial process (containing referee reports and your response letters) to be published as an online supplement to each paper. If you should prefer removal of any referee-only figures included in the point-by-point response(s), e.g. because they may still be used for future publication or because they have been reproduced from published work by others, please do let us know immediately via response email.
More information is available here: https://link.springer.com/partners/embo-press/editorial-policies#Peer%20review

** Click here to be directed to your login page: https://emboj.msubmit.net

EMBO Press Author Checklist

Corresponding Author Name: Xavier Prieur	
Journal Submitted to: The EMBO Journal	
Manuscript Number: EMBOJ-2025-123308R	

USEFUL LINKS FOR COMPLETING THIS FORM
The EMBO Journal - Author Guidelines
EMBO Reports - Author Guidelines
Molecular Systems Biology - Author Guidelines
EMBO Molecular Medicine - Author Guidelines

Reporting Checklist for Life Science Articles (updated January
This checklist is adapted from Materials Design Analysis Reporting (MDAR) Checklist for Authors. MDAR establishes a minimum set of requirements in transparent reporting in the life sciences (see Statement of Task: 10.31222/osf.io/9sm4x). Please follow the journal's guidelines in preparing your
Please note that a copy of this checklist will be published alongside your article.

Abridged guidelines for figures
1. Data
The data shown in figures should satisfy the following conditions:
- the data were obtained and processed according to the field's best practice and are presented to reflect the results of the experiments in an accurate and unbiased manner.
- ideally, figure panels should include only measurements that are directly comparable to each other and obtained with the same assay.
- plots include clearly labeled error bars for independent experiments and sample sizes. Unless justified, error bars should not be shown for technical
- if n<5, the individual data points from each experiment should be plotted. Any statistical test employed should be justified.
- Source Data should be included to report the data underlying figures according to the guidelines set out in the authorship guidelines on Data

2. Captions
Each figure caption should contain the following information, for each panel where they are relevant:
- a specification of the experimental system investigated (eg cell line, species name).
- the assay(s) and method(s) used to carry out the reported observations and measurements.
- an explicit mention of the biological and chemical entity(ies) that are being measured.
- an explicit mention of the biological and chemical entity(ies) that are altered/varied/perturbed in a controlled manner.
- the exact sample size (n) for each experimental group/condition, given as a number, not a range;
- a description of the sample collection allowing the reader to understand whether the samples represent technical or biological replicates (including how many animals, litters, cultures, etc.).
- a statement of how many times the experiment shown was independently replicated in the laboratory.
- definitions of statistical methods and measures:
  - common tests, such as t-test (please specify whether paired vs. unpaired), simple χ2 tests, Wilcoxon and Mann-Whitney tests, can be unambiguously identified by name only, but more complex techniques should be described in the methods section;
  - are tests one-sided or two-sided?
  - are there adjustments for multiple comparisons?
  - exact statistical test results, e.g., P values = x but not P values < x;
  - definition of 'center values' as median or average;
  - definition of error bars as s.d. or s.e.m.

Please complete ALL of the questions below.
Select "Not Applicable" only when the requested information is not relevant for your study.

Materials

Newly Created Materials	Information included in the manuscript?	In which section is the information available? (Reagents and Tools Table, Materials and Methods, Figures, Data Availability Section)
New materials and reagents need to be available; do any restrictions apply?	Yes	Materials and methods

Antibodies	Information included in the manuscript?	In which section is the information available? (Reagents and Tools Table, Materials and Methods, Figures, Data Availability Section)
For **antibodies** provide the following information:<br>- Commercial antibodies: RRID (if possible) or supplier name, catalogue number and or/clone number<br>- Non-commercial: RRID or citation	Yes	Reagents and tools tables

DNA and RNA sequences	Information included in the manuscript?	In which section is the information available? (Reagents and Tools Table, Materials and Methods, Figures, Data Availability Section)
**Short novel DNA or RNA including primers, probes**: provide the sequences.	Yes	Materials and methods

Cell materials	Information included in the manuscript?	In which section is the information available? (Reagents and Tools Table, Materials and Methods, Figures, Data Availability Section)
**Cell lines:** Provide species information, strain. Provide accession number in repository **OR** supplier name, catalog number, clone number, and/**OR** RRID.	Yes	Materials and methods
**Primary cultures:** Provide species, strain, sex of origin, genetic modification status.	Not Applicable	
Report if the cell lines were recently **authenticated** (e.g., by STR profiling) and tested for mycoplasma contamination.	Yes	Materials and methods

Experimental animals	Information included in the manuscript?	In which section is the information available? (Reagents and Tools Table, Materials and Methods, Figures, Data Availability Section)
**Laboratory animals or Model organisms:** Provide species, strain, sex, age, genetic modification status. Provide accession number in repository **OR** supplier name, catalog number, clone number, **OR** RRID.	Yes	Materials and methods
**Animal observed in or captured from the field:** Provide species, sex, and age where possible.	Not Applicable	
Please detail **housing and husbandry conditions**.	Yes	Materials and methods

Plants and microbes	Information included in the manuscript?	In which section is the information available? (Reagents and Tools Table, Materials and Methods, Figures, Data Availability Section)
**Plants:** provide species and strain, ecotype and cultivar where relevant, unique accession number if available, and source (including location for collected wild specimens).	Not Applicable	
**Microbes:** provide species and strain, unique accession number if available, and source.	Not Applicable	

Human research participants	Information included in the manuscript?	In which section is the information available? (Reagents and Tools Table, Materials and Methods, Figures, Data Availability Section)
If collected and within the bounds of privacy constraints report on age, sex and gender or ethnicity for all study participants.	Not Applicable	

Core facilities	Information included in the manuscript?	In which section is the information available? (Reagents and Tools Table, Materials and Methods, Figures, Data Availability Section)
If your work benefited from core facilities, was their service mentioned in the acknowledgments section?	Yes	Acknowledgements

Design

Study protocol	Information included in the manuscript?	In which section is the information available? (Reagents and Tools Table, Materials and Methods, Figures, Data Availability Section)
If study protocol has been **pre-registered, provide DOI in the manuscript**. For clinical trials, provide the trial registration number **OR** cite DOI.	Not Applicable	
Report the **clinical trial registration number** (at ClinicalTrials.gov or equivalent), where applicable.	Not Applicable	

Laboratory protocol	Information included in the manuscript?	In which section is the information available? (Reagents and Tools Table, Materials and Methods, Figures, Data Availability Section)
Provide DOI OR other citation details if **external detailed step-by-step protocols** are available.	Not Applicable	

Experimental study design and statistics	Information included in the manuscript?	In which section is the information available? (Reagents and Tools Table, Materials and Methods, Figures, Data Availability Section)
Include a statement about **sample size** estimate even if no statistical methods were used.	Yes	In the figure legend
Were any steps taken to minimize the effects of subjective bias when allocating animals/samples to treatment (e.g. **randomization procedure**)? If yes, have they been described?	Yes	Materials and methods
Include a statement about **blinding** even if no blinding was done.	Yes	Materials and methods
Describe **inclusion/exclusion criteria** if samples or animals were excluded from the analysis. Were the criteria pre-established?<br><br>If sample or data points were omitted from analysis, report if this was due to attrition or intentional exclusion and provide justification.	Not Applicable	No exclusion was applied.
For every figure, are **statistical tests** justified as appropriate? Do the data meet the assumptions of the tests (e.g., normal distribution)? Describe any methods used to assess it. Is there an estimate of variation within each group of data? Is the variance similar between the groups that are being statistically compared?	Yes	Figure Legends

Sample definition and in-laboratory replication	Information included in the manuscript?	In which section is the information available? (Reagents and Tools Table, Materials and Methods, Figures, Data Availability Section)
In the figure legends: state number of times the experiment was **replicated** in laboratory.	Yes	Figure Legends
In the figure legends: define whether data describe **technical or biological replicates**.	Yes	Figure Legends

Ethics

Ethics	Information included in the manuscript?	In which section is the information available? (Reagents and Tools Table, Materials and Methods, Figures, Data Availability Section)
Studies involving **human participants**: State details of **authority granting ethics approval** (IRB or equivalent committee(s), provide reference number for approval.	Not Applicable	
Studies involving **human participants**: Include a statement confirming that **informed consent** was obtained from all subjects and that the experiments conformed to the principles set out in the WMA Declaration of Helsinki and the Department of Health and Human Services Belmont Report.	Not Applicable	
Studies involving **human participants:** For publication of **patient photos**, include a statement confirming that consent to publish was obtained.	Not Applicable	
Studies involving experimental **animals**: State details of **authority granting ethics approval** (IRB or equivalent committee(s), provide reference number for approval. Include a statement of compliance with ethical regulations.	Yes	Materials and methods
Studies involving **specimen and field samples:** State if relevant **permits** obtained, provide details of authority approving study; if none were required, explain why.	Not Applicable	

Dual Use Research of Concern (DURC)	Information included in the manuscript?	In which section is the information available? (Reagents and Tools Table, Materials and Methods, Figures, Data Availability Section)
Could your study fall under dual use research restrictions? Please check biosecurity documents and list of **select agents and toxins** (CDC): https://www.selectagents.gov/sat/list.htm	Not Applicable	
If you used a select agent, is the security level of the lab appropriate and reported in the manuscript?	Not Applicable	
If a study is subject to dual use research of concern regulations, is the name of the **authority granting approval and reference number** for the regulatory approval provided in the manuscript?	Not Applicable	

Reporting

The MDAR framework recommends adoption of discipline-specific guidelines, established and endorsed through community initiatives. Journals have their own policy about requiring specific guidelines and recommendations to complement MDAR.

Adherence to community standards	Information included in the manuscript?	In which section is the information available? (Reagents and Tools Table, Materials and Methods, Figures, Data Availability Section)
State if relevant guidelines or checklists (e.g., **ICMJE, MIBBI, ARRIVE, PRISMA**) have been followed or provided.	Yes	Materials and methods
For **tumor marker prognostic studies,** we recommend that you follow the **REMARK** reporting guidelines (see link list at top right). See author guidelines, under 'Reporting Guidelines'. Please confirm you have followed these guidelines.	Not Applicable	
For **phase II and III randomized controlled trials**, please refer to the **CONSORT** flow diagram (see link list at top right) and submit the CONSORT checklist (see link list at top right) with your submission. See author guidelines, under 'Reporting Guidelines'. Please confirm you have submitted this list.	Not Applicable	

Data Availability

Data availability	Information included in the manuscript?	In which section is the information available? (Reagents and Tools Table, Materials and Methods, Figures, Data Availability Section)
Have **primary datasets** been deposited according to the journal's guidelines (see 'Data Deposition' section) and the respective accession numbers provided in the Data Availability Section?	Yes	**Data availability**
Were **human clinical and genomic datasets** deposited in a public access-controlled repository in accordance to ethical obligations to the patients and to the applicable consent agreement?	Not Applicable	
Are **computational models** that are central and integral to a study available without restrictions in a machine-readable form? Were the relevant accession numbers or links provided?	Yes	**Data availability**
If publicly available data were reused, provide the respective **data citations in the reference list**.	Yes	References
