# Supplementary material for: Tripartite ER-mitochondria-lipid droplets contact sites control adipocyte metabolic flexibility
Source: EMBO J. 2026 Jul 22;45(17):6021–53. doi: 10.1038/s44318-026-00876-z (PMC13534470; doi:10.1038/s44318-026-00876-z)
Supplement: Supplementary file 8 [file 44318_2026_876_MOESM3_ESM.zip › Movie_EV1.rtf]

Movie EV1 shows 3D reconstructions from FIB-SEM acquisitions of adipose tissue sections from control mice. The animation combines aligned raw image stacks with organelle segmentation, enabling visualization of the spatial organization of lipid droplets and mitochondria within the tissue.
