# Supplementary material for: Tripartite ER-mitochondria-lipid droplets contact sites control adipocyte metabolic flexibility
Source: EMBO J. 2026 Jul 22;45(17):6021–53. doi: 10.1038/s44318-026-00876-z (PMC13534470; doi:10.1038/s44318-026-00876-z)
Supplement: Supplementary file 10 [file 44318_2026_876_MOESM5_ESM.zip › Movie_EV3.rtf]

Movie EV4 shows 3D reconstructions from FIB-SEM acquisitions of 3T3-L1 adipocytes treated with NTC control siRNA. The animation combines aligned raw image stacks with organelle segmentation, enabling visualization of the spatial organization of lipid droplets and mitochondria within the adipocytes
